# Supplementary material for: Structural variation in 1,019 diverse humans based on long-read sequencing
Source: Nature. 2025 Jul 23;644(8076):442–52. doi: 10.1038/s41586-025-09290-7 (PMC12350158; doi:10.1038/s41586-025-09290-7)
Supplement: Supplementary file 1 — This file contains Supplementary Notes 1–11 and Figs. 1–72 and their legends. [file 41586_2025_9290_MOESM1_ESM.pdf]

nature portfolio

https://doi.org/10.1038/s41586-025-09290-7

Supplementary information

Structural variation in 1,019 diverse humans based on long-read sequencing

In the format provided by the authors and unedited

Supplementary Notes

Supplementary Note 1

Phasing Quality and Haplotype tagging of ONT reads using a high-coverage short-read 1kGP reference. We used WhatsHap[1] to phase the raw genotypes of the NYGC dataset using the LRS data of our resource and compare it against the statistical phasing performed in the same study to assess the quality of our LRS resource. We observed an excellent agreement of the long-read based phasing to the NYGC statistical phasing[2] with an average switch error rate of 1.24% (**Fig. S5a**). The trio-based phasing and the long-read trio based phasing for the four trios also showed good agreement. The trio phasing had an average switch error rate of 0.84% and 0.21% for the parents and the children respectively. The long-read trio phasing had an average switch error rate of 0.93% and 0.35% for the parents and children respectively (**Fig. S5b**). We observe a phased block length with a median NG50 of 1,578,731 bp, as well as an excellent agreement with previous population based phasing[2], indicating that the phased panels from NYGC are a good resource to tag the reads and provide additional information to the SV discovery and SV genotyping tools.

We used the haplotagging command in WhatsHap[1], which utilises information from a given phased panel to tag reads as either coming from haplotype 1 or haplotype 2. The ONT reads were haplotype-tagged (haplotagged) using the NYGC statistical phased VCF using WhatsHap's haplotag function. 69.9% of the ONT reads were tagged on average (**Fig. S53**) and utilised downstream by SVarp and Giggles to improve SV calling and genotyping.

Supplementary Note 2

Counts of added SV alleles during graph augmentation. SAGA introduces newly discovered SVs into the graph by using pseudo-haplotypes and the minigraph toolkit. This process does not yield a one-to-one mapping of SVs that were fed into the graph and the SVs that are retrieved after genotyping and phasing. To assess how many alleles are incorporated, we proceeded as follows: We compared alignments of pseudo-haplotypes and HPRC assemblies to the resulting graph and in this way identified SVs exclusive to the pseudo-haplotypes. Of the 189,142 pseudo-haplotype-exclusive SV alleles given to Giggles as input, we successfully genotyped and phased (using SHAPEIT5) 83,965 alleles, which corresponds to a 44.4% recovery of novel SV alleles. The drop in SV alleles can be attributed to two factors: (1) The genotyping algorithm relies on sequence-to-graph alignments which we pursue using minigraph. As the graph becomes more complex due to added pseudo-haplotypes, the minimizer-based approach used by minigraph struggles with alignments spanning complete bubbles. (2) Newly discovered SV alleles can contribute to the complexity of already existing bubbles, which makes genotyping more challenging as Giggles now may need to distinguish between more alleles, for instance at multi-allelic VNTR sites. Therefore, an increased number of SV alleles do not pass the final filtering criteria after genotyping, which is especially pronounced for multi-allelic, as well as at putatively complex sites (i.e., sites that are neither represented as clearcut insertions nor as deletions). In summary, the SAGA pipeline is comparatively stringent when adding new alleles to the graph and only retains those that can be reliably genotyped. We believe that this is indeed currently the correct approach in order to provide a high-quality resource to the community. With improved tools (specifically for sequence-to-graph alignment), we anticipate that in the future it may be possible to recover an increased number of SV alleles from our dataset.

VNTR sites in our SAGA-based resource. Out of the insertions classified as VNTRs, 28,781 (84.6%) are simple VNTRs with just one motif, whereas 5,225 (15.4%) are complex VNTRs involving two or more repeat motifs. Overall, the VNTRs in our SAGA-based resource comprise 22,829 distinct motifs, with motif length ranging from 1 bp to 481 bp (median 18 bp) and the length of the respective expansion/contraction ranging from 9 bp to 19.6 kb (median 95 bp). To allow us to more systematically capture VNTR complexity, we additionally genotyped VNTR sites across our resource using vamos (**Note S5**).

Supplementary Note 3

Copy-number accuracy estimation using intensity rank sum (IRS) testing. We also used the IRS test[3] to generate an additional estimate for the FDR for copy-number unbalanced SVs. This test leverages differences in microarray[4,5] probe-level intensities between samples expected to have different copy number states, and as such its detection power is limited to regions represented with Affymetrix SNP6 array probes generated for 1kGP samples[3]. From our final SV call set with 908 samples, microarray probe-level intensities were available for 901 samples. To use the IRS test, we first lifted all array probes to the CHM13 assembly using liftOver[6] with the GRCh38 to CHM13 chain file. We then extracted all deletions and duplications and applied the IRS test from the SVAnnotator tool of Genome STRiP[7] with the input VCF files and the previously used array intensity file[3] lifted to CHM13. We further subdivided the CHM13 genome into "difficult" regions and "high-confident" regions using BED files provided by the Genome in a Bottle (GiaB) project[8], namely the CHM13_notinalldifficultregions.bed.gz **(Fig. S54)**. Using the microarrays[4,5] we estimate an FDR of 8.06% for biallelic deletions, which improves to 6.97% in the GiaB high-confidence genomic regions, consistent with a high quality for our SAGA-based SV resource **(Fig. S54)**.

Haplotype QV estimation and insertion accuracy estimation. To facilitate efficient estimation of the average quality value (QV) of reconstructed haplotypes and the accuracy of novel insertions, we utilised the NYGC deep-coverage short-read 1kGP data[2] to build compacted de Bruijn graphs from error-corrected reads using lighter[9] and bcalm2[10]. We first applied lighter[9] to the short-read FASTQ files using a k-mer length of 23 and the options "-trim -discard" to allow trimming of reads and discarding reads that cannot be corrected. All successfully corrected reads were provided as input to bcalm2[10] to construct a compacted de Bruijn graph from the sequencing data using a k-mer length of 61 and a required minimum k-mer abundance of 3.

To estimate QV values for reconstructed haplotypes, we first implanted for each sample all phased variants (SNPs, InDels and SVs) into the CHM13 reference using bcftools consensus[11] for haplotype 1 (option "-H 1"), called sample.h1.fa, and haplotype 2 (option "-H 2"), called sample.h2.fa. We then used yak count (https://github.com/lh3/yak) on the matched compacted de Bruijn graph to build a short-read based k-mer hash table, called sample.dbg.yak, using "-K 1.5g -k 31". With the reconstructed haplotype and the k-mer hash table, we then calculated a haplotype QV estimate using yak qv with the options "-p -K 1.5g -l 100k sample.dbg.yak sample.h[1|2].fa" for haplotype 1 and 2, respectively. We estimate an average quality value (QV) score of 37.6 for the haplotype blocks constructed with SHAPEIT5 when compared to sample-matched compacted de Bruijn graphs **(Fig. S55)**. These scores are on par with QV scores previously derived from Pacific Biosciences (PacBio) continuous long read assemblies[12], consistent with high data quality.

To estimate the insertion accuracy, we augmented the CHM13 reference with all new insertions and then mapped all short-read derived compacted de Bruijn graph sequences against the extended reference using bwa mem[13]. For a singleton SV, occurring in only 1 sample at allele count 1, the expected coverage is 1× and we therefore computed for all insertions the average depth using samtools depth[11] with the option "-aa". For FDR estimation, an insertion with average coverage less than 1x was counted as a false positive. This

procedure yielded a genome-wide FDR estimate for insertions of 11.40% (8.43% in GiaB high-confidence regions) **(Fig. S56)**.

Call set comparison to multi-platform whole-genome assemblies. We used SV calls recently generated by the HGSVC[14] as ground truth to derive false negative (FN), false positive (FP) and true positive (TP) estimates for insertions and deletions called by the SAGA framework in the 16 overlapping samples (**Fig. S7**). From the HGSVC3 release directory[14] on the IGSR FTP site with the URL https://ftp.1000genomes.ebi.ac.uk/vol1/ftp/data_collections/HGSVC3/release/ we downloaded the CHM13 variant calls variants_T2T-CHM13_sv_insdel_alt_HGSVC2024v1.0.vcf.gz.

We then subsetted the autosomal HGSVC3 variant calls to the 16 overlapping samples, requiring a minimum allele count of 1, i.e., at least one SV carrier among the 16 samples. We carried out the same subsetting to autosomal variants in the 16 overlapping samples for the SAGA based SV callset. Afterwards we compared VCFs using Truvari[15] with the options `--pick multi -p 0.5 -P 0.25` to account for representation differences of assembly-based SV calls in comparison to graph-based SV calls (see below). For each SV call we also computed its size and MAF using `bcftools +fill-tags` to stratify the comparison by these SV parameters. For both, the Giggles genotyped callset **(Fig. ED2)** and the more stringently filtered final callset **(Fig. ED2)**, we observed no prominent peaks of FN SV calls with respect to SV size **(Fig. ED2),** but note an enrichment of false negative SV sites near telomeres **(Fig. S57)**. Since our graph augmentation pipeline uses GRCh38 as the underlying sequence for pseudo-haplotype integration (see Methods), one would indeed expect such an enrichment of FN SVs in the telomeres as these sequences are typically unresolved in the GRCh38 reference genome and thus, excluded from pseudo-haplotype integration. In terms of MAF **(Fig. ED2)**, we observe an increased false negative rate for rare SVs, which is likely a result of our study design that uses only intermediate ONT sequencing coverage. The higher ONT error rate and shorter N50 read length may also lead to a slight increase in FNs for rare insertions compared to deletions where local assembly is required for graph integration. The genome-wide true positive rate (TPR) is 64.36% for deletions and 67.33% for insertions after genotyping with Giggles. In the strictly filtered final call set with a decrease in FPs but increased FNs, the true positive rate (TPR) is reduced to 47.40% for deletions and 51.76% for insertions, respectively. In line with our intermediate coverage study design, these TPR estimates improve for common SVs (MAF $\geq$ 0.1) to a TPR of 79.59% for deletions and 83.24% for insertions after genotyping (60.07% and 66.81%, respectively, in the final call set).

In terms of false positives, we observe an enrichment of FP SV calls for shorter SVs compared to larger SVs. To gain further insight into these false positive SVs, we first annotated all SVs for tandem repeats using the TRF[16] CHM13 annotation track and labelled SVs as tandem repeat related if their start position overlapped a TRF site by $\geq$ 50bp. We observed that smaller SVs were greatly enriched for tandem repeats (62.02% for SVs < 250bp compared to 16.88% for SVs $\geq$ 250bp). This prompted us to investigate tandem repeat loci in more detail and as shown in **Fig. S12** we observed large discordances in SV representation for pangenome graph-derived SV calls in comparison to classical assembly-based variant calling approaches. This leads to large differences in reported SV size, type and location which are only incompletely taken into account by our Truvari approach, where we could harmonize only a subset of representation differences using the new Truvari refinement option and by using relatively lenient options for SV size and sequence similarity. Another noticeable limitation of the minigraph-based graph augmentation strategy is a general under-representation of the full allelic spectrum present at tandem repeat loci, which is due to the fact that the minigraph tool[17] currently only incorporates new alleles if they differ by at least 50 bp. Although this strategy prevents severe graph fragmentation, the effect this may have on representing VNTRs has been noted previously[17]. Consequently, we observe for many VNTR loci less alleles compared to assembly-based approaches, i.e., for the locus in **Fig. S12** HGSVC reports 9 SV calls (7 in the 16 overlapping samples) from 63 high-quality assemblies compared to 6 SV calls reported by SAGA in 967 samples. This reduced tandem

repeat allele spectrum results in further differences in reported SV size and type that might inflate our FP estimates compared to assembly-based calls. We conclude that variations in short tandem repeats (STRs) and VNTRs pose challenges for the generation and comparison of population SV callsets as large discordances in representation are to be expected between pangenome-based SV calling approaches compared to classical assembly-based approaches.

These observations prompted us to provide a separate vamos-based resource of VNTR genotypes from our data resource, which does not utilise the graph and is thus not affected by the aforementioned limitations in SV representation (**Note S5**). Additionally, these findings prompted us to assess other non-tandem repeat related classes of SVs, particularly MEIs, in detail. For the three main transposon categories (LINE/L1, SINE/*Alu* and SVA) called by SAGA and annotated using SVAN we could compare MEI SV calls to the HGSVC3 MEI call set[14] (Mobile_Elements/1.0/MEI_Callset_T2T-CHM13.ALL.20240918.csv.gz in the HGSVC3 release directory) for the 16 overlapping samples. Overall, for insertions greater than 50 bp our SAGA framework identified 23,204 *Alu* elements, 4,850 L1 elements and 3,188 SVA elements in the 967 samples of the augmented graph. A considerable fraction of these MEIs were classified as non-canonical by SVAN, namely 1,781 *Alu* elements (8.31%), 498 L1s (11.44%) and 796 SVAs (33.28%), where we expected annotation and classification differences among SAGA and the HGSVC3 MEI call set.

For each sample in the subset of 16 common samples, we compared each transposable element class separately for all autosomes by selecting the MEI category of interest, added 100 bp to either side of the insertion point and then assessed the number of shared and unique MEI calls using bedtools intersect. For FDR estimation, we assumed that the HGSVC3 call set is complete and hence, any unmatched ONT MEI call was counted as a false positive (as previously using Truvari). This approach revealed an estimated FDR of 3.94% for canonical *Alu* insertions, 6.75% for canonical L1 insertions, and 0.85% for canonical SVA element insertions in our SAGA based callset (**Fig. S13**). We note however that when assessing all MEIs including non-canonical events the estimated FDR is higher, namely 13.66% for *Alu* (canonical + non-canonical), 24.63% for L1 (canonical + non-canonical), and 45.31% for SVA (canonical + non-canonical) – which is likely attributed to differences in the annotation of non-canonical MEIs between studies and calling algorithms, including unidentified non-canonical MEI calls in the Logsdon *et al.* study[14] (**Fig. S14**). To estimate sensitivity, we again assumed that the HGSVC3 call set[14] is error free and evaluated the number of shared MEI calls compared to the total number of MEI calls present in the HGSVC3 call set. This approach yielded sensitivity estimates of 90.59% for *Alu*s, 85.22% for L1 insertions and 84.29% for SVA insertions using canonical and non-canonical MEIs, in our SAGA-based callset. We did not observe a consistent trend in MEI calling sensitivity estimates with respect to coverage or N50 read length in these 16 overlapping samples (**Fig. S15**).

For all classes of autosomal insertions that occur outside of a tandem-repeat context (n=37,834; 43.72% of all insertions), namely interspersed duplications, NUMTs, processed pseudogenes, HERVK insertions, MEIs and unclassified insertions, the estimated FDR is 10.36% using all insertions called from whole-genome assemblies generated by the HGSVC3 as the ground truth.

For biallelic deletions of transposable elements, we observed on average 1,068 *Alu* deletions, 141 L1 deletions and 51 SVA deletions per autosomal genome in the 16 samples overlapping samples with HGSVC3. The estimated FDR is 1.14% for *Alu* deletions, 3.88% for L1 deletions and 7.15% for SVA deletions. The overall FDR for autosomal mobile element deletions, including orphaned deletions, with 1,334 SVs per genome is 1.94% compared to SVs from whole-genome assemblies[14] generated by the HGSVC.

Comparison to gnomAD-SV v4. In order to compare our SAGA-based SV call set to gnomAD-SV version 4, we first needed to lift our CHM13-based SV calls to GRCh38. While the liftOver tool works well for

single-nucleotide variants (point events), it has shortcomings for SVs and tandem repeat variations because different genome assemblies may show distinct reference alleles. Therefore, SV records are more frequently deleted or erroneously lifted using liftOver; a classic example are insertions of mobile elements with respect to CHM13 that are part of the GRCh38 reference, where liftOver tends to frequently introduce false insertions into GRCh38.

To circumvent these problems, we devised a workflow that takes advantage of our phased SAGA-based SV call set available for 908 samples. We first implanted all our SVs into the CHM13 reference using bcftools[11] consensus with the option '-H 1' and '-H 2' to create the respective two haplotypes. We then aligned the CHM13-based haplotypes to GRCh38 using minimap2[18] and called SVs with respect to GRCh38 using svim-asm[19]. Finally, we subsetted all variants to insertions or deletions greater or equal to 50 bp and left-aligned all SVs using bcftools norm. We merged all samples into a multi-sample SV VCF file using bcftools merge and removed all variants with an allele count of 1,816, as these variants are present in all haplotypes of the 908 samples and accordingly, these SVs reflect reference differences between CHM13 and GRCh38.

Using this derived GRCh38-based SV call set, we compared all insertions with gnomAD based on their integration site (+/-100 bp), but regardless of their length, since, for example, short-read based mobile element prediction methods show differences in the insertion length distribution compared to long-reads (**Fig. S21**). Using SVAN annotations for the GRCh38 call set, we carried out a comparison stratified by mobile element class (*Alu*, Line1 and SVA) and for all insertions. This analysis revealed that a large proportion (50.86%) of the insertions in our resource are not currently reported in gnomAD, which includes 8,077 *Alu* (34.75%), 2,586 L1 (52.89%), and 1,269 SVA elements (47.12%). For deletions, we required a 25% reciprocal overlap or that the deletion start and end coordinates differ by less than 500 bp while preserving a size ratio of smaller to larger deletion of at least 0.25. We carried out a comparison for all deletions, where 14.46% are not currently present in gnomAD, and for deletions greater 250 bp in size, where 7.84% are not currently present in gnomAD.

Supplementary Note 4

Quality assessments of the Giggles genotypes. We performed quality assessments of the genotypes produced by Giggles after the callset was filtered by dividing the samples into bins corresponding to median sequencing coverage ranges and read N50 ranges. We selected ranges with more than 30 samples to ensure confidence in the results. **Table S27** shows the number of samples in each range. The value for the median coverage was obtained from the alignment of the reads to the CHM13 reference. We performed quality assessment using Hardy-Weinberg equilibrium plots and compared the allele frequency of variants in the input panel against the genotyped callset. **Figures S58** and **S59** show that the genotyping was robust for various coverage ranges and read N50 ranges.

SV genotyping vs. SV discovery in single samples. We also compared the number of SVs discovered by DELLY – both from single sample calls and population calls – against the number of SVs genotyped by Giggles through the SAGA framework. We divided the samples into the different bins as mentioned in the preceding section to investigate the effect of coverage and read N50 on the discovery and genotyping parts of the framework. **Figures S60** and **S61** show the per-sample count of SVs (sum of heterozygous sites and twice of homozygous SV sites) along with stratification of samples with AFR and non-AFR ancestry. In **Figure S60**, we observe that the count of SVs genotyped by Giggles and discovered by DELLY population calls does not change considerably, while the single sample DELLY calls are more noticeably affected by lower coverage. Furthermore, as shown in **Figure S61**, we identify no marked effect of read N50 on SV genotyping or discovery.

SVs in a typical human genome based on SAGA. As another means of assessing our SAGA-based resource we performed an analysis of the number of SVs per class seen in a typical genome, thereby distinguishing between donors with ancestries from AFR and elsewhere (non-AFR). Utilising all classified SVs identified using SAGA we find a median of 11,023 deletions in the AFR (non-AFR: 8,989), 12,178 insertions (non-AFR: 9,658), and 728 putatively complex sites that cannot be resolved into either deletions or insertions (non-AFR: 626). Further resolving SVs by class using SVAN, AFR samples exhibit a median of 5,785 deletions affecting VNTR sequence (non-AFR: 4,793), 6,728 VNTR expansions (non-AFR: 5,363), 2,026 tandem (non-AFR: 1,693), 149 interspersed (non-AFR: 117) and 4 complex duplications (non-AFR: 3), 1,737 MEIs (non-AFR: 1,248), 371 non-canonical MEIs (non-AFR: 296), 754 deletions of mobile elements (non-AFR: 605), 7 insertions (non-AFR: 5) and 6 deletions (non-AFR: 5) of processed pseudogenes, 9 insertions of LTRs or LTR flanked HERVK elements (non-AFR: 6), and 6 NUMTs (non-AFR: 4) (**Table S17**).

Supplementary Note 5

Genotyping VNTRs using vamos. We performed a separate analysis of VNTRs on the ONT data from this study using vamos (version 2.1.3)[20]. Using a publicly available VNTR motif dataset[21], we called VNTRs on the ONT data on the set of positions labelled as 'VNTR' in the sites list. Since the data is not haplotype-resolved, we ran vamos in the 'read' mode, creating unphased VNTR calls. We provide the VNTR calls as a single multisample VCF in our release.

To assess the quality of these VNTR calls, we additionally ran vamos on a recently generated set of multi-platform whole genome assemblies[14] (HGSVC3) for the overlapping sample set of 16 samples (**Fig. S7**). We used the same version of vamos and sites lists for this comparison, and ran vamos in the 'assembly' mode creating phased VNTR calls. To compare the unphased calls from the ONT data to the phased calls from the HGSVC3 assemblies, we paired the ONT alleles to the HGSVC alleles which had the smallest difference in repeat unit counts. For the comparison, we removed the VNTR alleles where the alleles from our resource were close in length to alleles genotyped from the HGSVC assemblies. We defined the two lengths to be close if the length of the VNTR allele from our resource was between 90% to 110% of the length of the allele from the HGSVC assemblies. For the remaining sites, we calculated the Pearson's Correlation Coefficient (PCC) for all 16 samples to investigate concordance. We also calculated the PCC by taking data from mismatching samples (ONT VNTRs from one sample compared against the HGSVC3 assemblies of another sample) to establish a baseline of matching random VNTR calls. **Figure S30** shows the PCC of same-sample matches is more than the PCC for a mismatched sample comparison (median PCC of 0.8996 for same-sample and median PCC of 0.5727 for sample mismatch).

Analysis of the VNTR diversity based on vamos. We compared the diversity of the VNTRs called by vamos on the ONT data of this study (for 1019 samples) against calls on the HGSVC3 assemblies[14] (for 64 samples – we excluded HG00514, since the authors reported issues with the respective assemblies[14]). From the input list of 370,468 sites, vamos genotyped 369,685 sites with three or more VNTR alleles, 660 sites with two alleles, and 79 sites with one VNTR allele from the ONT data of this study. From the HGSVC3 assemblies, vamos identified 58,407 sites with three or more alleles, 65,618 sites with two alleles and 246,364 sites with only one VNTR allele genotyped. In particular, we investigated the VNTR diversity at the sites provided by considering the count of repeat units at different percentile ranges. For each VNTR site, we considered three ranges in terms of repeat unit counts: the range of the 25th percentile to the 75th percentile, of the 5th percentile to the 95th percentile, and of the 1st percentile to the 99th percentile of repeat unit counts. We reasoned that these quantiles are relevant for users of our resource as they, for instance, enable an assessment of whether a repeat unit count observed in a patient genome is exceptionally high or low

compared to samples from a diversity of individuals from the general population. We determined these percentile repeat unit counts for both the HGSVC3 assemblies and the ONT data for each VNTR position. We compared the two datasets for VNTR diversity by taking the difference of the repeat unit count at the higher percentile and the smaller percentile. **Figure ED4** shows the density plot comparing different percentile ranges. We observe that as the percentile range is increased, the data shifts from the y = x line.

To quantify this, we calculated the number of data points above the line y = x + c (these represent the data where the ONT data from our resource shows a larger range) and below the line y = x - c (these represent the data where the HGSVC assemblies show a larger range). We computed the change as the percentage increase in the number of points where our ONT data resource shows a higher range as compared to the HGSVC3 data (**Table S31**). We observe that the change was low for the 25th-75th percentile (-7.25%) and 5th-95th percentile range (29.57%), indicating that the estimate of these lower quantiles are broadly consistent between both data sets. However, we observe significant change for the 1st-99th percentile range (333.51%) – with most (81.26%) of the extreme repeat unit counts coming from our resource in this percentile range. We attribute this to an improved ability to estimate more extreme quantiles in VNTR repeat unit counts in our resource, as a consequence of the larger number of human samples (*N*=1019) included.

Explorative analysis of rare disease-associated VNTR sites. To provide further reassurance of the quality of our data and of its potential utility, we analysed the *PLIN4* VNTR locus associated with late-onset myopathy[22] and the *ABCA7* VNTR locus associated with Alzheimer disease[23] from the vamos-based VNTR callset produced from our resource. We identified the *PLIN4* VNTR at chr19:4494323-4497243 and the *ABCA7* VNTR at chr19:1012105-1014401 in the sites list given to vamos in this study. For both loci, we investigated the VNTR allele diversity reported in this study (*N*=1019 samples) and compared it to a current multi-platform whole genome assembly effort (HGSVC3) pursued in a smaller sample set[14] (*N*=65 samples). The repeat unit and base pair length diversity matched well for the 25th percentile to 75th percentile, and the 5th percentile to 95th percentile range (**Table S16**) – repeat unit ranges thought to not result in a clinical phenotype. For the *PLIN4* locus, the two datasets also agree at the 1st percentile to 99th percentile, however the ONT data from our resource shows one clear outlier allele with 43 repeat units (**Fig. ED4**). Manual inspection of the site in the outlier sample NA20127 using the Integrative Genomics Viewer (IGV) reassuringly verifies the presence of several ONT long reads containing large insertions of a length equivalent to the reported 43 repeat units (**Fig. S62**), in support of the heterozygosity of the sample NA20127 for a late-onset disease-associated allele.

For the *ABCA7* locus associated with Alzheimer's disease, the ONT data from our resource demonstrate a wider range of VNTR repeat unit counts compared to the HGSVC3 dataset (**Table S16**, **Fig. ED4**). This includes several alleles longer than the previously reported 5,720 bp length cutoff thought to separate wildtype and disease-causing VNTR alleles[23]. In particular, our resource comprises 4 samples homozygous for the expanded allele and 40 heterozygous samples. Considering the late onset of the diseases associated with SVs at *PLIN4* and *ABCA7*[22,23], long-read based analyses may allow observation of disease-associated VNTRs before phenotypic effects are observed.

Supplementary Note 6

Comprehensive capture of inversions irrespective of their size. Our initial exploration revealed that inversions frequently escape automated detection in long reads owing to inaccuracies in read mapping particularly affecting inversions smaller than 1 kb, which appear as clustered mismatches in minimap2 alignments (**Fig. S32**). To allow capturing these inversions, we examined strategies for inversion discovery by simulating inversions of varied sizes. We generated simulated ONT reads from these augmented genomes, using SURVIVOR[24], mimicking the sequencing coverage in our resource (median coverage of

17X with reads averaging 20 kb in length). We simulated inversions of varied sizes, ranging from 50 to 1,000 bp. These reads were then aligned to a specified 250 kb segment of chromosome 1 from the GRCh38 reference genome using the minimap2 and NGMLR aligners, and as a next step, inversion detection was performed with Sniffles and DELLY. **Figure S33** shows that minimap2 exhibits a very high mismatch rate in regions containing inversions smaller than 1 kb, whereas NGMLR alignments do not suffer from this drawback in small inversion regions. This superior performance of NGMLR, particularly in small and complex genomic regions, is likely attributed to its sophisticated scoring mechanism, tailored to accommodate the complexities of long reads[25]. Concurrently, **Figure S63** illustrates that DELLY is more effective than Sniffles in detecting inversions up to 1 kb in length, suggesting it represents a more suitable methodology for identifying small inversions.

Our revised inversion detection workflow identified 733 inversions, comprising 134 SVs identified through our custom workflow, 317 detected with DELLY based on minimap2 alignments, 40 found via Sniffles, and 311 events primarily defined as insertions but later reclassified as inverted duplications through SVAN. Among these 733 inversions, we find 257 balanced (or "simple") inversions with a median length of 1,565 bp, which are further broken down into 45 inversions bordered by various repeat classes in inverted orientation based on alignment dotplot analysis, implying formation through homology-directed repair (HDR) processes or non-allelic homologous recombination (NAHR). These flanking inverted repeats include: Alu (*N*=10 inverted element pairs), L1 (8 pairs), LTR (4 pairs) and low-complexity repeats (4 pairs), as well as SDs up to 75.5 kb in length in inverted orientation (19 pairs). We do not find inversions flanked by hundreds of kb of SD sequence, in spite of their commonness in the genome, consistent with these SVs requiring long-read lengths in excess of the SD length for their detection[26]. Genome assemblies incorporating ultra-long reads[27,28] or Strand-seq[26,29] will be required to resolve these SVs at the population-scale in the future.

Inversion genotyping with GeONTIpe. Inversion genotyping was performed using the GeONTIpe pipeline (https://github.com/RMoreiraP/GeONTIpe). 78% of inversions were successfully genotyped, after excluding inverted duplications which were not reliably genotyped due to pipeline limitations. The majority of inversions are rare, predominantly exhibiting low allele count (AC) values (<10 AC) (**Fig. ED3**). Only a small subset of inversions is observed more frequently across populations, with 37 inversions exceeding an AC of 1000. This pattern is similarly reflected in the AF distribution: while inversions span a broad range of frequencies (>1% to 50%), 70% of inversions have an AF <0.01, whereas only 19% exhibit an AF ≥ 0.1. In terms of heterozygosity, AFR samples display the highest inversion heterozygosity, consistent with the expected trend observed for other variant classes (**Fig. S64**); conversely, EAS samples show the lowest inversion heterozygosity, mirroring the heterozygosity distribution seen for other variants.

Supplementary Note 7

Polarisation to the Chimp Genome. We sought to identify SV alleles present in a recently generated *Pan troglodytes* (chimpanzee) whole genome reference assembly[30]. We processed the reference sequence into overlapping synthetic reads of sizes ranging from 4 kb to 500 kb and aligned the synthetic reads using minigraph[17] to the HPRC_mg_44+966 augmented pangenome. The alignments show the paths in the bubbles of the graph that the chimpanzee reference spans, which is considered the ancestral allele for the bubble. Out of the 220,174 bubbles present in HPRC_mg_44+966, 73.08% of the bubbles show the ancestral allele to be the human reference sequence. 17.38% of the bubbles show an alternate path as the ancestral allele, while the aligning failed in 9.54% of the bubbles, indicating significant divergence of the ancestral allele from the graph sequences.

Since the SVs of the final callset of the SAGA framework are obtained through a bubble decomposition step

(see **Methods**) and underwent filtering steps, we attempted to match the ancestral allele with the SVs of the final callset through the nodes of the graph that constitute the ancestral allele and the SV allele. Our findings show that, for 71.16% of the evaluated sites, the reference allele corresponds to the ancestral allele, while 16.37% of SVs represent the ancestral allele. Additionally, 2.96% of SV sites had an unknown ancestral allele due to the inability of the aligner to map into the respective region, and 9.52% exhibited an ancestral allele that did not match either the SV or reference allele, indicating potential multiallelic variation at these loci (**Fig. S65**).

Effects of polarisation on SV classes. We used the ancestral alleles of our SV calls to polarise the SVs relative to the chimpanzee genome. This polarisation of our callset by ancestral alleles converted 14,816 deletions into insertions and 7,189 insertions into deletions. Additionally, 4,945 deletions and 6,781 insertions had ancestral alleles distinct from both the reference and alternate alleles of our callset. These SVs are classified as 'putatively complex' with respect to the chimpanzee genome (**Table S24**).

By leveraging the SV classifications from SVAN and our breakpoint homology analysis, we obtained an SV class-specific perspective on polarisation (**Table S25**). The changes in SV class distribution resulting from polarisation are visualised in **Figure ED5**, which shows the distribution of SV length, homology, and class before (**Fig. S66a**, same as **Fig. 5b**) and after polarisation (**Fig. S66b**).

Notably, we find that the number of mobile element deletions decreased by 55.3%, while the number of mobile element insertions increased by 5.6% during polarisation. Among *Alu* elements, the majority (88.05%) of the younger *AluY* family members were reclassified from deletions to insertions, whereas most members of the older *AluS* family (88.5%) retained their original SV type. As a result, the proportion of older *AluS* elements in deletions after polarisation increased to 35.1%, compared to 4.9% before polarisation (**Fig. S67**). This likely reflects the occurrence of deletions over time accumulating at older mobile element insertion event sites (and potentially also, but likely to much a lesser extent, polymorphisms shared between humans and chimpanzees).

Additionally, a substantial fraction of SVs classified as VNTRs (7,981 SVs, 19.7% of all VNTRs with an ancestral allele), and to a lesser extent those classified as tandem duplications (1,273 SVs, 9.77% of all DUPs with an ancestral allele), could not be definitively assigned to either the reference or alternate allele. For VNTRs this is likely due to the complexities of defining ancestral states in regions with multiallelic structural variation.

Notably, we also observed differences in reclassification rates between insertions and deletions in the case of *Alu*-mediated, homology-driven SVs. Specifically, the majority (63 out of 80 SVs, 87.5%) of such events initially called as insertions were reclassified as deletions, while only a small proportion (123 out of 3260 SVs, 3.8%) of *Alu*-mediated, homology-driven SVs originally called as deletions were reclassified as insertions. This is consistent with homology-mediated SV formation involving flanking *Alu* elements (TEMR) resulting largely in deletions, and only much more rarely in other SV classes[31].

The group of *Alu*-mediated SVs classified as insertions after polarisation was further analysed, revealing a high sequence content derived from mobile elements (**Fig. S68**). Specifically, 45.5% of *Alu*-mediated insertions and only 24.6% of *Alu*-mediated deletions contained more than 90% of bases derived from mobile elements (SINE; LINE; LTR). This suggests that members of the *Alu*-mediated insertions group after polarisation may often represent misclassified complex and nested mobile element insertion events, rather than *Alu*-mediated SVs.

Supplementary Note 8

Filtering SVs in rare disease patients. The prioritisation of potentially clinically relevant SVs from a genome-wide variant callset is a well-known problem in identifying causative variants for rare-disease patients. Population databases that remove common variants are a fundamental resource for such prioritisation[32], but such resources are not yet widely established for long-read SV datasets. Therefore, we assessed whether variation represented in the augmented pangenome graph could help prioritising potentially pathogenic SVs. To assess the performance on SV filtering in a rare disease context, we compared SV callsets from four deeply long-read sequenced patient genomes, A, B, C and D (average coverage of, respectively, 21x, 33x, 39x and 39x) – all with an undisclosed rare disease phenotype – for which the causal variant is unknown. Two of the samples, C and D, are from two siblings. We used parental data to generate phased assemblies for all four samples, which were then used to call genomic variants against the CHM13 linear reference genome with PAV[33] (as described further below), an assembly-based variant caller that calls SVs as well as smaller polymorphisms. Additionally, we also called variants against CHM13 using Sniffles[34], an alignment-based SV caller. For both callsets, in each sample, we removed the SVs found in the CenSat v2.1 regions[35]. We compared the two SV callsets, obtained with different approaches, with both the HGSVC multi-platform long-read assembly based SV callset[14] as well as our 1kGP ONT SV resource callset before genotyping, produced by SAGA. Two variants were considered 'the same' where we found a reciprocal overlap of their coordinates of at least 50% or where there was a maximum distance of 200 bp between them, with their size difference being at most 25%. The statistics for these comparisons are summarised in **Figure S46**.

The total number of SVs present in both the PAV and Sniffles callsets amounted to 15,501, 16,037, 16,888 and 16,941 for sample A, B, C and D, respectively. From those, there were 160, 159, 187 and 180 SVs, respectively, present in neither the HGSVC multi-platform genome assembly[14] dataset nor in our resource. These variants are candidate causal alleles. Overall, SV filtering enabled a reduction in the number of candidate SVs of 98.97%, 99.01%, 98.89% and 98.94% for samples A, B, C and D, respectively. Additionally, incorporating our SV resource enabled the reduction of candidate SVs by more than half (54.7%-56.4%) when compared to using the HGSVC multi-platform whole-genome assemblies[14] alone. This notable improvement results from the ability to filter out low allele frequency SV alleles using our resource (**Fig. S47, S48**).

At the same time, we observed 1,745, 2,025, 2,140 and 2,094 SVs, respectively, that intersect with the multi-platform genome assembly callset, but not with our SAGA-based resource. This effect could be explained by variants inaccessible by our ONT sequencing protocol, which we characterize as regions covered by an average of less than 5x (see **Fig. S69**), as well as multiallelic sequence contexts exhibiting different variant representations between genome assembly and graph structures, which complicates the comparison of such SV sites across variant callsets[14]. To evaluate potential effects of graph structures and variant representation, we realigned all non-intersecting SV alleles to the graph, using GraphAligner[36]. For the four patient genomes, between 70.96% and 72.10% of these SVs clearly aligned to the augmented graph (**Table S26**), showing that they are present in the SAGA callset. These analyses highlight the need to develop better sequence-to-graph alignment tools to improve SV callset intersections in long-read SV studies in the future.

Further methodological details with respect to these patient genome analyses are provided below:

Sample and DNA extraction to allow for patient genome sequencing. Three families of rare disease patients were enrolled and were recruited following approval from the Ethics Committee, Medical Faculty,

Heinrich Heine University, Düsseldorf, Germany (Ethical vote 2018-268). Informed consent was obtained from all participants prior to inclusion in the study. The four affected individuals, including one sibling pair, were diagnosed with the same unresolved genetic syndrome (phenotype undisclosed, and not relevant, to this study). They were sequenced in a trio/quartet design together with their parents.

High-molecular-weight (HMW) DNA was isolated using the Monarch HMW DNA extraction kit for cells & blood (CAT# T3050L, New England Biolabs) according to the manufacturer's instructions of protocol type "High Molecular Weight DNA Extraction from blood" with an input amount of 500 µl frozen EDTA-stabilized blood and a lysis speed of 1400 rpm. Until further processing, the samples were stored at 4°C. DNA purity and concentration were determined using a NanoDrop spectrophotometer (CAT# ND-1000, Thermo Fisher Scientific).

HiFi sequencing. Library preparation for whole genome HiFi (high fidelity) single molecule, real time (SMRT) sequencing was performed with the protocol "Preparing HiFi SMRTbell® Libraries using the SMRTbell Express Template Prep Kit 2.0" (PN101-853-100 Version 04; April 2021; Pacific Biosciences) for sample A and with the protocol "Preparing whole genome and metagenome libraries using SMRTbell prep kit 3.0" (PN 102-166-600; Apr 2022) for samples B, C and D.

For sample A, 6 µg of HMW DNA of sample A were sheared with the Megaruptor 2 (Diagenode, MA, USA), targeting a mean fragment size between 15-20 kb. Subsequently, the sample was processed according to the manufacturer's instructions. In short, single-strand overhangs of the sheared DNA were digested, damaged DNA repaired and a single adenine nucleotide added to the 3'-end of DNA fragments. Afterwards, a SMRTbell overhang adapter was ligated to each end of the fragments and the library purified using AMPure® PB beads. Incomplete ligated or damaged DNA fragments were digested with a nuclease treatment and purified.

For samples B, C and D, between 5-6 µg of HMW DNA were used for shearing with hydropores (Megaruptor 3 DNAFluid+ and the Megaruptor 3 shearing-kit (Diagenode, MA, USA)) to ~20 kb. The samples were then processed according to the protocol and all required reagents were included in the SMRTbell® prep kit 3.0 (PacBio, CA, USA). The library preparation workflow follows the previously mentioned workflow, except that barcoded adapters were used during the adapter ligation step.

To reduce the amount of smaller fragments, size selection was performed using the BluePippin System and a 0.75% agarose cassette (SageScience, MA, USA) with a 10 or 12 kb lower cut-off. SMRT sequencing was performed on the Sequel II/e systems (PacBio, CA, USA). The complex binding of each library was performed according to the manufacturer's protocol, using either the sequencing primer v5 (sample A) or v3.2 (samples B, C, D, respectively). Each complex-bounded library was sequenced on three SMRT Cells 8 M (PacBio, CA, USA), using adaptive loading and a 30 hrs movie, 2 hrs immobilization and 2 hrs pre-extension time with a targeted on plate loading concentration (OPLC) of 75 pM (sample A) or 85 pM (samples B, C, D, respectively). Circular consensus sequencing (CCS) reads were generated and (if required) barcodes trimmed using the default settings in SMRT Link v10 (sample A) or v11 (samples B, C, D, respectively), with a minimum of three passes and minimum read quality of 0.99.

Reference genome alignments. Reads from these four patient genomes were aligned to the CHM13 linear reference genome using pbmm2's (version 1.9.0) "align" subcommand to map the HiFi reads using the option "--sort" to generate sorted BAM files.

Individual assembly generation. Individual phased assemblies of these four patient genomes were generated using hifiasm[37] (version 0.19.8-r603) trio binning mode. In order to do so, k-mer counts for parental reads were required, and for that we used the yak[37] (version 0.1-r56) count subcommand with the

“-b37” option, generating both a maternal and paternal k-mer count yak file for each sample. We then used these yak files as input parameters for hifiasm using the “-1” and “-2” options to generate a phased assembly, with one GFA file per haplotype.

SV calling in four patient genomes using linear reference genomes. We used Sniffles[34] version 2.0.7 and Phased Assembly Variant Caller (PAV)[33] version 2.3.4 to discover SVs in the four patient genomes using the CHM13 linear reference genome. For Sniffles, an alignment-based caller, we used BAM files to compute candidate SVs for each sample in a VCF file. For PAV, an assembly-based caller, we converted the GFA phased assemblies to FASTA, and used them as input to PAV, together with the CHM13 linear reference genome, in order to generate a VCF with genomic variants. Furthermore, we filtered PAV results to include only variants bigger than 50 bp in size, creating a PAV SV set.

Filtering SVs in four patient genomes using our SAGA-based SV resource. Filtered SV sets were obtained by excluding all variants overlapping the centromere/satellite repeat annotation (Cen/Sat) version 2.1 for the CHM13 linear reference genome. We used the bedtools’[38] (version 2.30.0) subtract subcommand with the VCF files and the BED file with the Cen/Sat positions, creating a new VCF file without SVs overlapping with those regions.

We utilised a callset comparison pipeline to compare different VCF files. This comparison considers two variants as ‘matching’ if their coordinates reciprocally overlap by at least 50% or there is a maximum distance of 200 bp between them and they differ in size by at most 25%. With this, we obtained VCF files of the intersections between different input VCF files, together with summary files for these intersections.

For the comparison across SV resources, we utilised a VCF file with all SVs from a recent multi-platform whole genome assembly study (HGSVC3)[14]. To obtain that file, we used bcftools’[11] (version 1.21) fill-tags plugin using “-t 'INFO/END:1=int(POS+INFO/SVLEN)'” option to add the END tag to the inversions VCF file. Subsequently, we used the bcftools concat subcommand to concatenate that VCF with the VCF with the SV insertions and deletions, using the “-a” option to allow for overlaps. We compared the VCF files using always the same order, as the order dictates which variant representation is used as the base for comparison. First, we read the PAV callset, then we compared the Sniffles callset with it, then the SAGA callset and, lastly, the HGSVC3 callset. Allele frequency distribution plots for subsets of the intersections are shown in **Figures S47** and **S48.**

Variant allele alignment to the graph in four patient genomes. We extracted variant allele sequences from VCF files, generating a FASTA file with one record per variant, with the ALT sequence from the VCF plus an anchor sequence of 1,000 bp on each side, extracted from the CHM13 linear reference genome. We then aligned these FASTA files to the augmented graph using GraphAligner[36] version 1.0.19, using the options “--multimap-score-fraction 1 --seeds-mxm-windowsize 5000 --seeds-mxm-length 30 --seeds-mem-count 10000 -C -1 --bandwidth 15 --discard-cigar”, creating GAF files with the alignments. Distribution plots for the sequence identity of the whole length alignments were generated(**Fig. S70**).

Supplementary Note 9

Variant filtering of validated pathogenic SVs from 31 published rare disease genomes. We also analysed the rate by which validated disease-causing SV alleles are filtered out based on our resource. To achieve this, we utilised a recently published dataset of 39 rare disease samples (previously sequenced to ~30X HiFi long reads on a PacBio *Revio* platform[39]) containing 40 validated pathogenic SVs potentially callable by long-read based split-read detection (**Table S29**). We focused our analysis on the subset of 31 rare disease genomes in which validated causal variants were identified by DELLY split read analysis, giving the same representation as the previously validated SV. In these genomes, DELLY called 35 validated

causal SVs (for comparison: pbsv[40] called 37 causal SVs in these genomes; see **Table S29**). We simulated a basic variant prioritisation workflow by calling SVs in these 31 samples with DELLY run in pangenome filtering mode (delly-pg, https://github.com/dellytools/delly).

In its pangenome mode, DELLY discovers and genotypes SVs in a primary long-read alignment (in this case GRCh38) in light of multiple alternative alignments. In this experiment, we used both CHM13 alignments and pangenome graph alignments to our augmented graph as alternative alignments. DELLY requires that all split-reads present in the primary alignment are also aligned in split-read manner in all alternative alignments, thereby removing candidate split-reads on GRCh38 with full-length alignments on CHM13 or the augmented graph. Using the DELLY pangenome mode, we assessed for each callset whether the relevant pathogenic variant was retained, and measured the total number of calls in the resulting call set. Notably, delly-pg retained 33/35 (94%) of the validated disease-causal SVs, while call sets were on average 83% smaller than in DELLY or 84% smaller than in pbsv in these samples (**Fig. S71a,b, Table S30**), implying potential of pangenome-based approaches for variant filtering to reduce the search space of potentially pathogenic SVs. The two SVs missed by delly-pg were (1) a ~150 bp expansion of the CGG short tandem repeat (STR) repeat in *FMR1* (**Fig. S71c**) and (2) a 49 kb deletion of a conserved non-coding elements (CNEs) regulating the *SHOX* gene (**Fig. S71d**). Importantly, variants of the *SHOX*-associated CNEs are recurrent and display incomplete penetrance[41,42]. These data suggest the presence of low-allele frequency variation at both of these SV sites in the general population; thereby, restricting our ability to filter them out based on using panels of normal individuals.

Details on the previously published 31 rare disease patient genome cohort. We utilised a previously published dataset of rare disease patient genomes sequenced to approximately 30X HiFi long reads on a PacBio Revio platform[39]. This dataset was specifically enriched for clinically relevant, hard-to-call pathogenic variants that are challenging to detect with traditional short-read whole genome sequencing approaches due to variant type or genomic context. Among all independently validated pathogenic SVs reported in these samples previously[39], 40 were considered likely to be split-read callable due to their breakpoints being outside of long segmental duplications, centromeres, acrocentric chromosome arms and the SV not being the result of unbalanced translocations (**Table S29**). We conservatively focused our analyses on these variants, to evaluate whether (and if so to what extent) DELLY would recommend filtering validated pathogenic SVs based on our SAGA SV resource. We repeated SV discovery in these previously published Hifi genomes using DELLY, identifying 35 of those SVs, which are spread across 31 rare disease samples. This shows that SVs in these Hifi genomes are indeed callable by DELLY, and we thus chose to use these 31 genomes to illustrate pangenome-based variant filtering of known disease-causing SVs.

Pangenome-based SV prioritisation in 31 patient genomes. For each sample, we created three SV callsets using (1) DELLY (v1.3.1) in pangenome mode, (2) DELLY in long read mode, and (3) pbsv (v2.9.0-4-g222d634). HiFi reads of all samples were aligned to the GRCh38 (accession GCA_000001405.15) and CHM13-T2T (v2.0) reference genomes using pbmm2 (v1.13.1.) and the extended pangenome graph using minigraph (v0.21-r606). These three alignments were used as input for DELLY (v1.3.1) in pangenome mode, while DELLY-default and pbsv used only hg38 aligned reads. We utilised a semi-automated process to test each of the 40 pathogenic SVs for re-call in the three callsets by (1) using bedtools intersect to search specifically for SVs in question in the expected regions, and (2) also verifying visually the calling status of each SV.

Supplementary Note 10

Targeted genotyping of challenging loci. We used Locityper[43] to genotype a set of highly polymorphic and medically relevant loci for all 1,019 ONT datasets. The 270 selected loci cover 15 Mb and overlap 463

protein coding genes, including 265 challenging medically relevant genes, as characterized by the Genome in a Bottle Consortium[44]; 20 polymorphic MUC genes[45] and lipoprotein(a)-encoding *LPA* gene[46]. For each locus and each ONT dataset, Locityper produced two local haplotypes based on the sequencing data and its database of locus alleles. Additionally, we reconstructed haplotypes from the phased NYGC call set; and compared two sets of haplotypes against the phased whole genome assemblies for 8 HGSVC and 1 HPRC samples. As a measure of genotyping accuracy, we evaluated sequence divergence between pairs of actual and predicted haplotypes.

At 191/270 (70.1%) polymorphic loci Locityper haplotypes were at least 0.1% more accurate than NYGC-callset based haplotypes (≥1% improvement at 96 loci). In contrast, the NYGC call set outperformed Locityper by 0.1% at only 7 loci (by 1% at 4 loci). Locityper improved genotyping accuracy by over 5% at 18 loci, covering 985 kb and completely encompassing 23 protein coding genes, including 8 mucin genes and tandem-repeat rich genes *GPI* and *LPA*. We conclude that our ONT-based dataset considerably outperforms the prior 1kGP dataset for making genotype assessments in these medically relevant regions.

Supplementary Note 11

DNA methylome analysis in a subset of ONT samples. Using Dorado (v0.7.2), we explored the analysis of DNA methylation from ONT signals across a representative selection of 66 samples drawn from our 1kGP ONT resource, yielding genome-wide 5mC modification likelihoods at single-base resolution. We made use of the haplotype calls from LociTyper to link individual reads to haplotypes at 294 clinically important genes. Across all CpG sites within these genes we observe a median of 80% methylation (min 73%; max 87%) across all samples (**Fig. S72A**). With the reads containing methylation calls linked to haplotypes we performed within sample haplotype specific differential methylation tests which has the benefit of controlling any sample level confounding factors by providing internal sample level control. To model haplotype specific differential methylation patterns (HDM) we applied a robust Mann Whitney test between the distribution of modification ratios for haplotype 1 and haplotype 2 at every gene for each of the samples independently. Every sample had at least one gene with significant HDM and most genes had at least one significant sample, suggesting that this test is sensitive towards local haplotype-specific imbalances in DNA methylation. We found 41 genes with significant HDM in 10 or more samples (**Fig. S72B**) including well known imprinted genes such as *HG19 and NLRP2*[47,48], which rank first and forth for the amount of HDM observed respectively. Encouragingly we also see high levels of HDM at the *HLA* locus and in particular at and nearby the *HLA-DRB5* locus (**Fig. S72C**). *HLA-DRB5* has been reported by several previous studies on HDM, with HDM at this locus suggested to contribute to the risk for conditions such as rheumatoid arthritis[49]. We note, however, that the gene content differs by HLA-DR haplogroup and only haplogroup DR2 carries the *HLA-DRB5* gene, warranting a haplogroup-aware analysis in future research.

Base modification calling of ONT reads. Raw fast5 files were converted to pod5 file format using ONT *pod5 convert fast5* v0.1.5. Then, pod5 files were used for simplex base and modification calling of 5mC and 5hmC using the super-accurate basecalling model "dna_r9.4.1_e8_sup@v3.3" with ONT *dorado basecaller* v0.7.2 (https://github.com/nanoporetech/dorado), and parameters "--emit-moves --device 'cuda:all' --no-trim --modified-bases '5mCG_5hmCG'". The resulting raw modbam files were input to *dorado trim* with default parameters for adapter trimming. Then, reads smaller than 50 bases or with mean quality of less than 7 were discarded with pysam v0.19.0 (https://github.com/pysam-developers/pysam).

Reads in modbam were transiently converted to fastq format and preserving all read tags with *samtools fastq* v1.17, using the parameter "-T '*'". The fastq reads were piped to Minimap2 v2.24-r1122 (Li, 2018) for alignment to GRCh38 using the parameters "-a -x map-ont --rmq=yes --MD --cs -L -y". After alignment, unmapped reads were discarded using *samtools view* v1.17. Read group tags with the sequencing flow cell id

was added to modbam files using *samtools addreplacerg*. Modbams were, then, pooled by sample name using *samtools merge* and sorted by coordinate and indexed with *samtools sort* and *samtools index* for downstream analyses.

Reads overlapping the 294 gene regions and with a matching read id from LociTyper were retained and assigned as haplotype 1 or haplotype2 in independent modbam files using *samtools view*. Modification ratios were computed using ONT *modkit pileup* v0.2.2 (https://nanoporetech.github.io/modkit/) over each haplotype with parameters "--sampling-frac 1 --seed 1234 ", and only restricting the pileup of modifications to CpG dinucleotides. To annotate the modification ratios of each CpG, bedmethyl files were overlapped with the 294 gene regions, using *bedtools intersect* v2.30.0[38].

Supplementary Figures

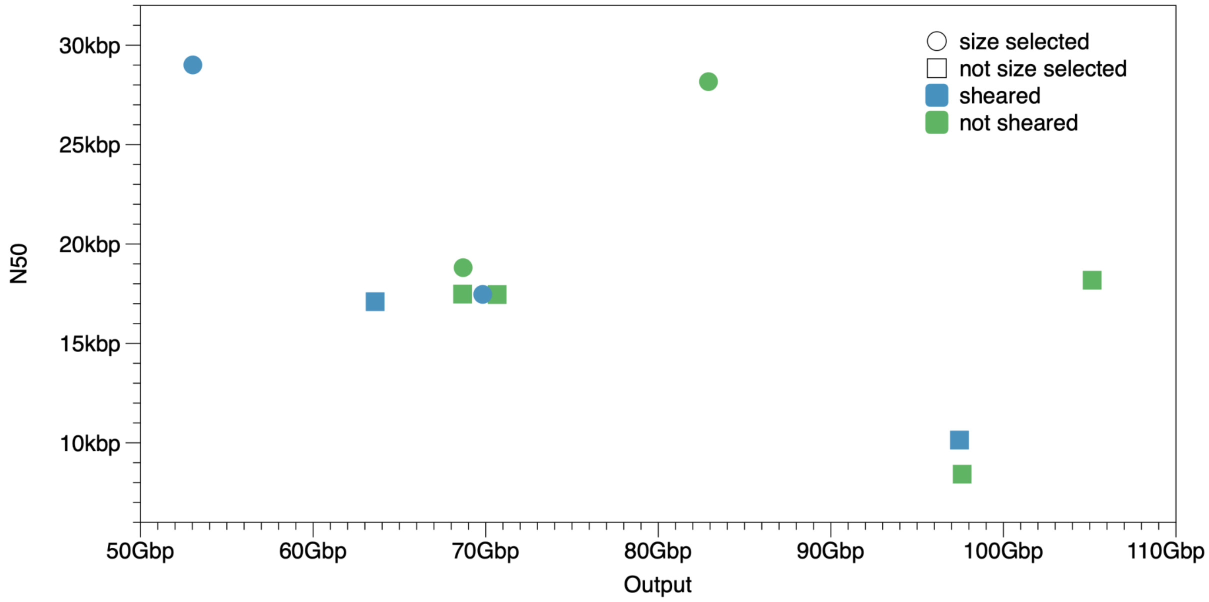

Supplementary Figure 1: Read-length N50 and output with and without size-selection for ≥25 kb fragments and needle shearing of DNA (sample HG00377 and HG02013).

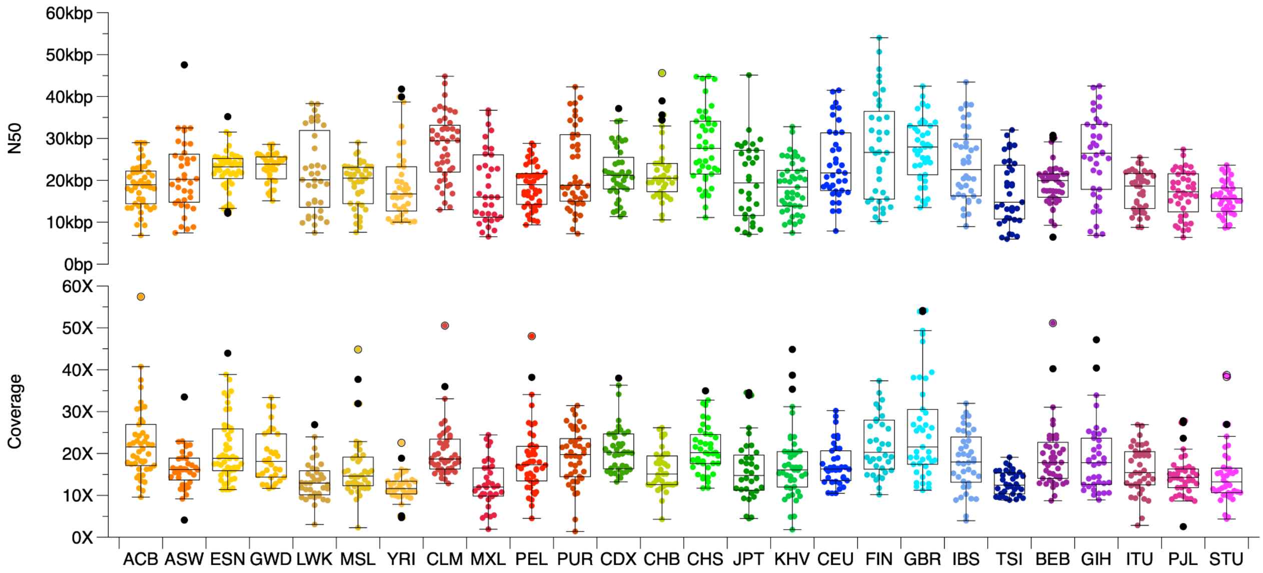

Supplementary Figure 2: Read-length N50 and fold-coverage for the 1,019 samples grouped by geographic location.

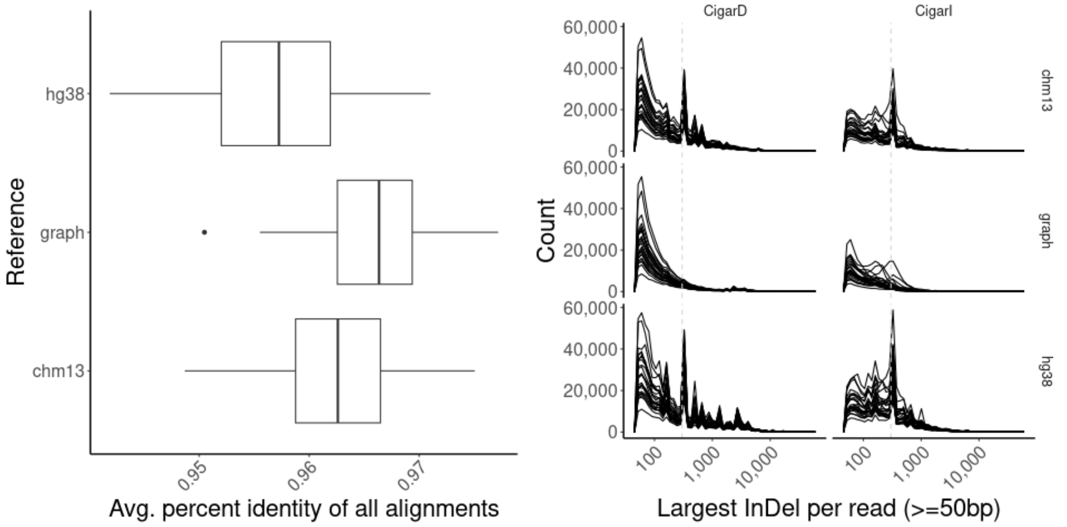

Supplementary Figure 3: Left: Average percent identity of all alignments (MAPQ > 0) of 25 random samples to the linear Genome Reference Consortium reference (hg38), the linear Telomere-to-Telomere consortium reference (chm13), and the pangenome minigraph reference from the HPRC (graph). **Right:** Distribution of the largest Cigar I (insertion) and Cigar D (deletion) operation for each read by sample. Vertical dashed line is at 300 bp to indicate the expected *Alu* peak for linear reference genomes.

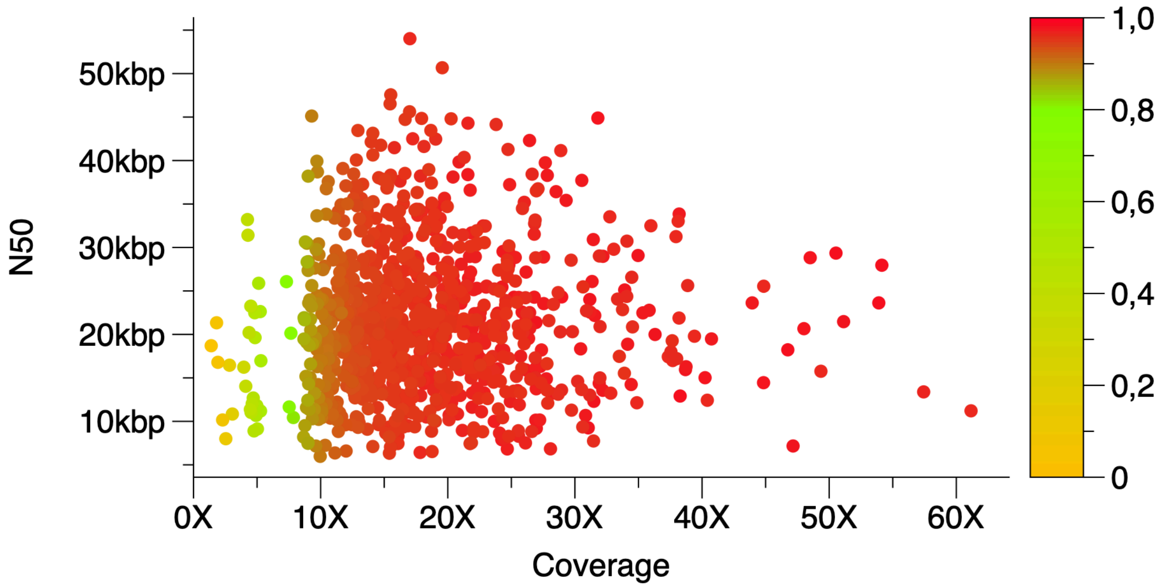

Supplementary Figure 4: Fold-coverage and read-length N50 for the 1,019 samples. Colors indicate the fraction of CHM13's bases covered at least five-fold.

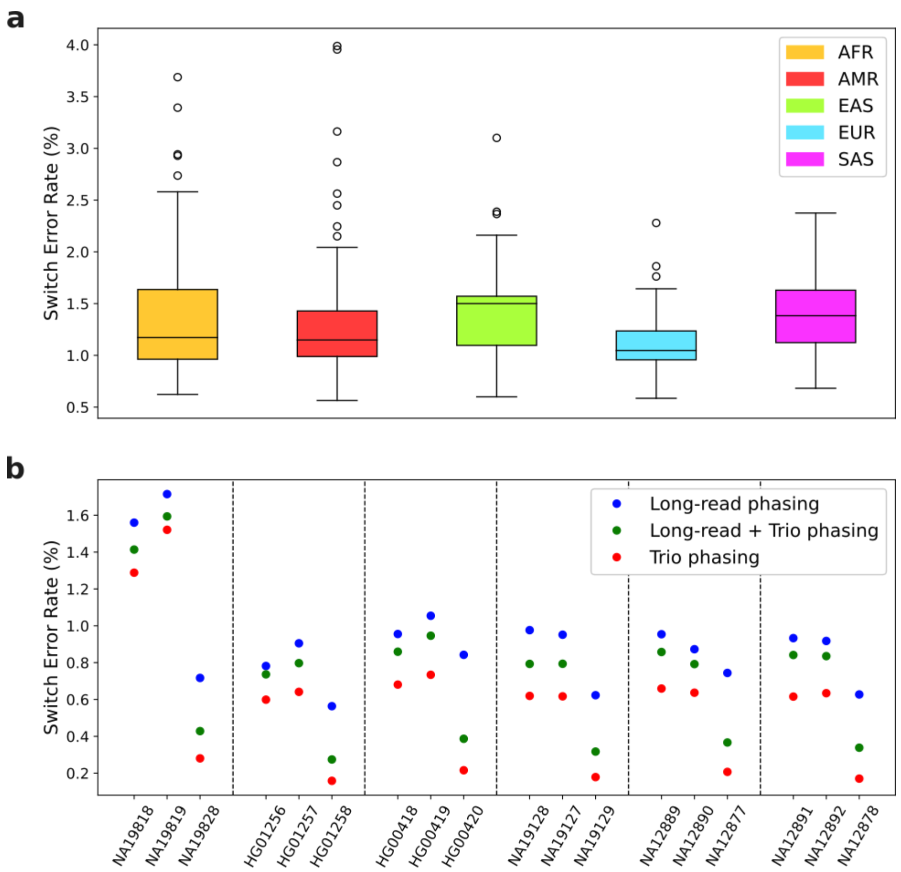

Supplementary Figure 5: Plotting the switch error rate (SER) between WhatsHap[1] phasing of the Byrska-Bishop *et al.*,[2] raw genotypes using the ONT reads from this study against the statistical phasing performed on the same genotypes. **a)** shows the SER for all the samples (grouped by population) for the comparison between long-read phasing and the statistical phasing. **b)** shows the SER for the samples in the 6 families for which ONT data is available. The SER is shown for the three different phasing strategies (long-read phasing, trio phasing and long-read trio phasing). The samples in each family are ordered as follows: father, mother and child.

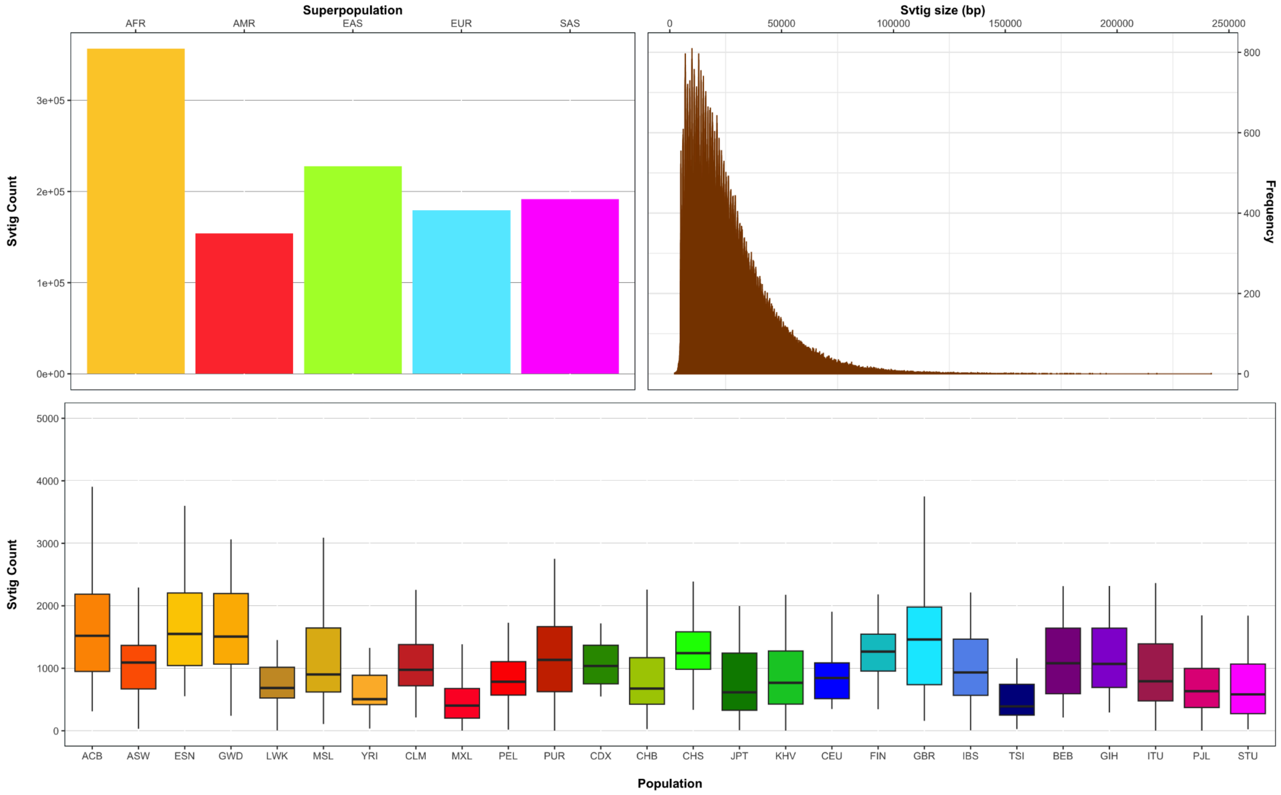

Supplementary Figure 6: Bar plot and the boxplot show svtig counts generated by SVarp per superpopulation and population respectively for 967 samples. The length frequency is also depicted by the histogram for the whole cohort. Note that numbers are based on the total number of svtigs in paternal and maternal haplotypes.

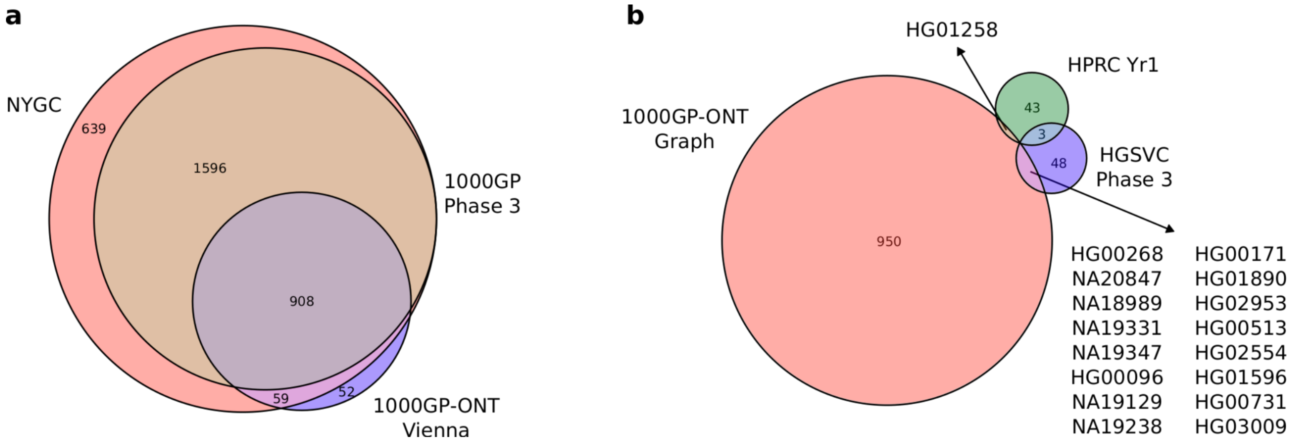

Supplementary Figure 7: a) Sample intersection of the 1,019 samples of this study with the 2504 sample list of the 1000 Genomes Project Phase 3[4] and the New York Genome Center (NYGC)[2] study with 3,202 samples (2,504 samples along with 698 trios). **b)** For the Graph Methods, Giggles and SVarp, we utilise the NYGC phased panel and hence these methods are restricted to the intersection of the 1000 Genomes ONT Vienna Project (this study) and the NYGC sample set. This figure shows the intersection of this sample set with the HPRC Yr1[50] and HGSVC Phase 3[14] sample sets.

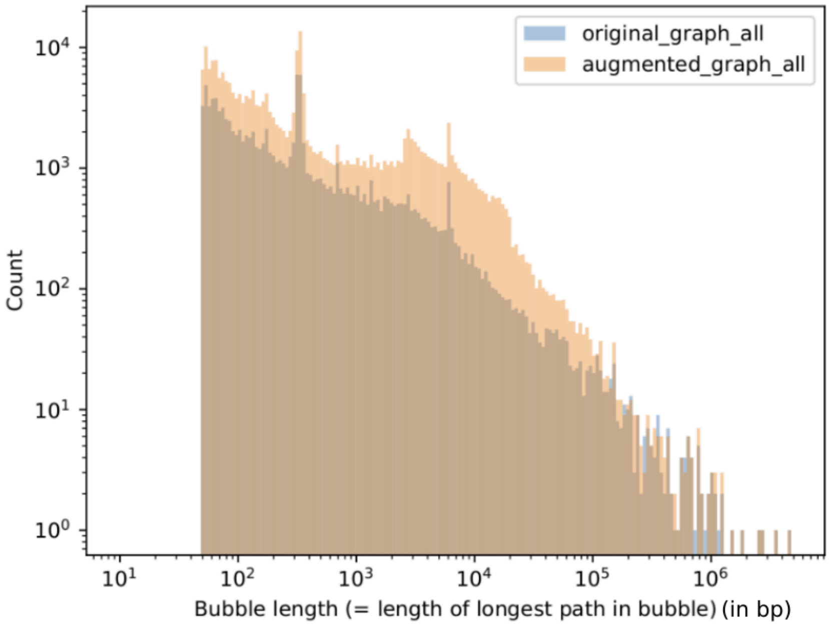

Supplementary Figure 8: Bubble-length histogram of the original and augmented graphs. The length of a bubble is defined as the length of the longest path through the bubble.

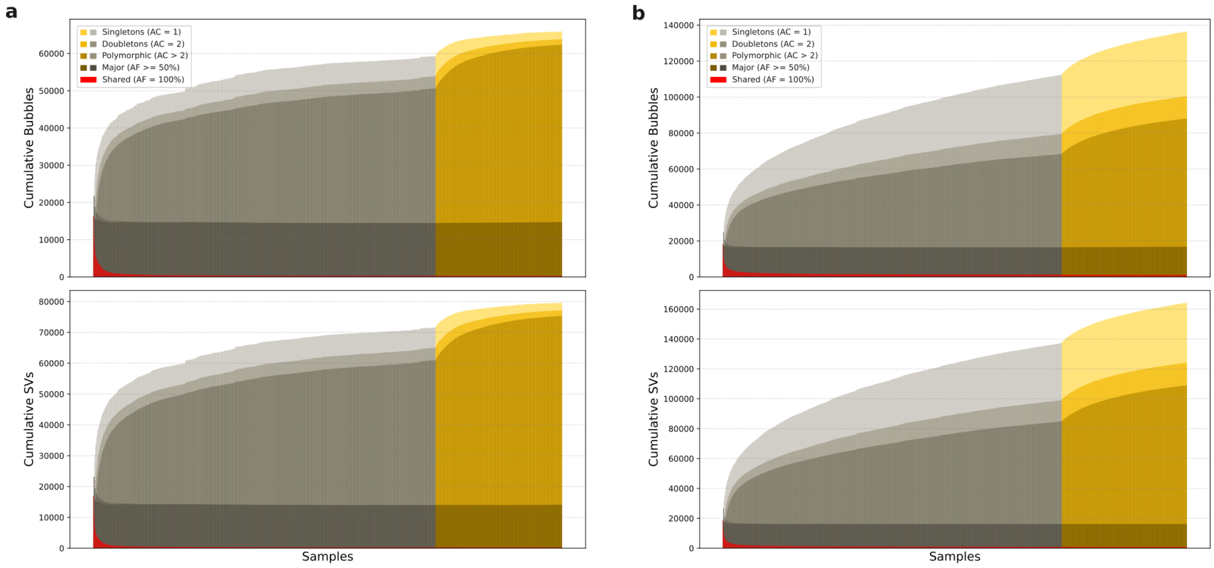

	Shared	Major	Polymorphic	Doubletons	Singletons
Bubbles	287	14,441	47,664	1,446	2,080
SVs	278	13,805	61,304	1,777	2,445

	Shared	Major	Polymorphic	Doubletons	Singletons
Bubbles	1,146	15,636	71,319	12,370	35,992
SVs	806	15,456	92,798	15,098	40,093

Supplementary Figure 9: Figure shows the cumulative growth curves for the SVs and the bubbles. **a)** shows the figures for the filtered genotypes of Giggles on the HPRC_mg and **b)** shows the figures for the final phased callset, which is the Giggles genotyped callset on the HPRC_mg_44+966 subsequently phased with SHAPEIT5[51]. The tables below show the count of each category after all the samples have been considered. The figure has been made using the 908 unrelated samples from our callset. The yellow part of the figure is used to denote the addition of AFR samples while the grey part is for non-AFR samples. The non-AFR samples have been randomly shuffled to reduce the effect of the non-AFR populations.

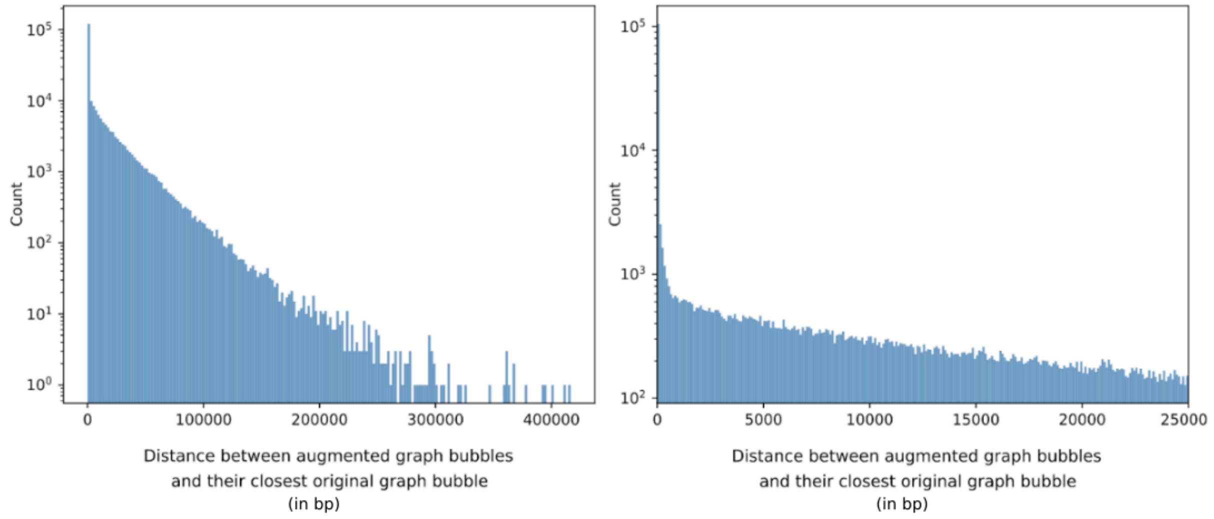

Supplementary Figure 10: Histogram showing the distances between bubbles in the augmented graph and their closest bubble in the original graph (in base pairs). The left panel shows the full histogram, the right panel shows only the part corresponding to distances up to 25,000 bp.

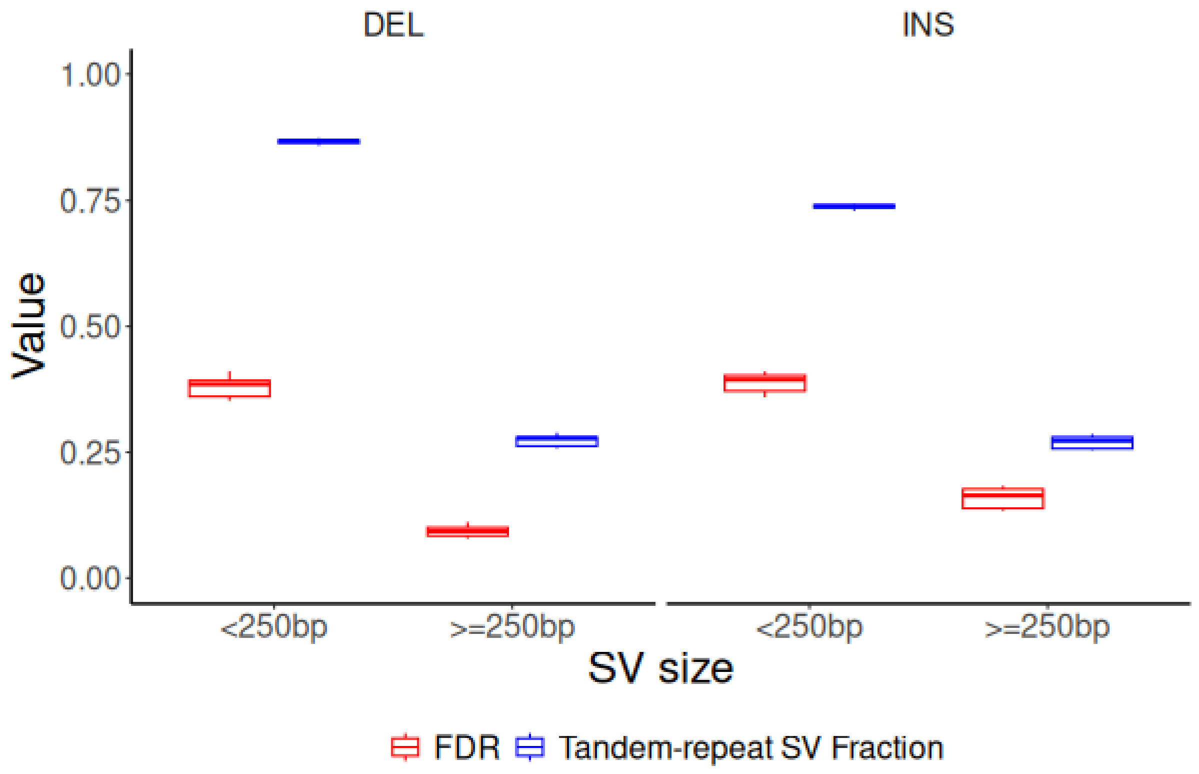

Supplementary Figure 11: Deletions (DEL) and insertions (INS) called by the SAGA framework compared to whole genome assemblies from HGSVC3 using one sample at a time for all the 16 overlapping samples (**Fig. S7**). The false discovery rate (FDR) for each sample is shown as a boxplot together with the fraction of SVs that overlap tandem repeats identified using TRF[16] stratified by SV size (<250 bp or >=250 bp).

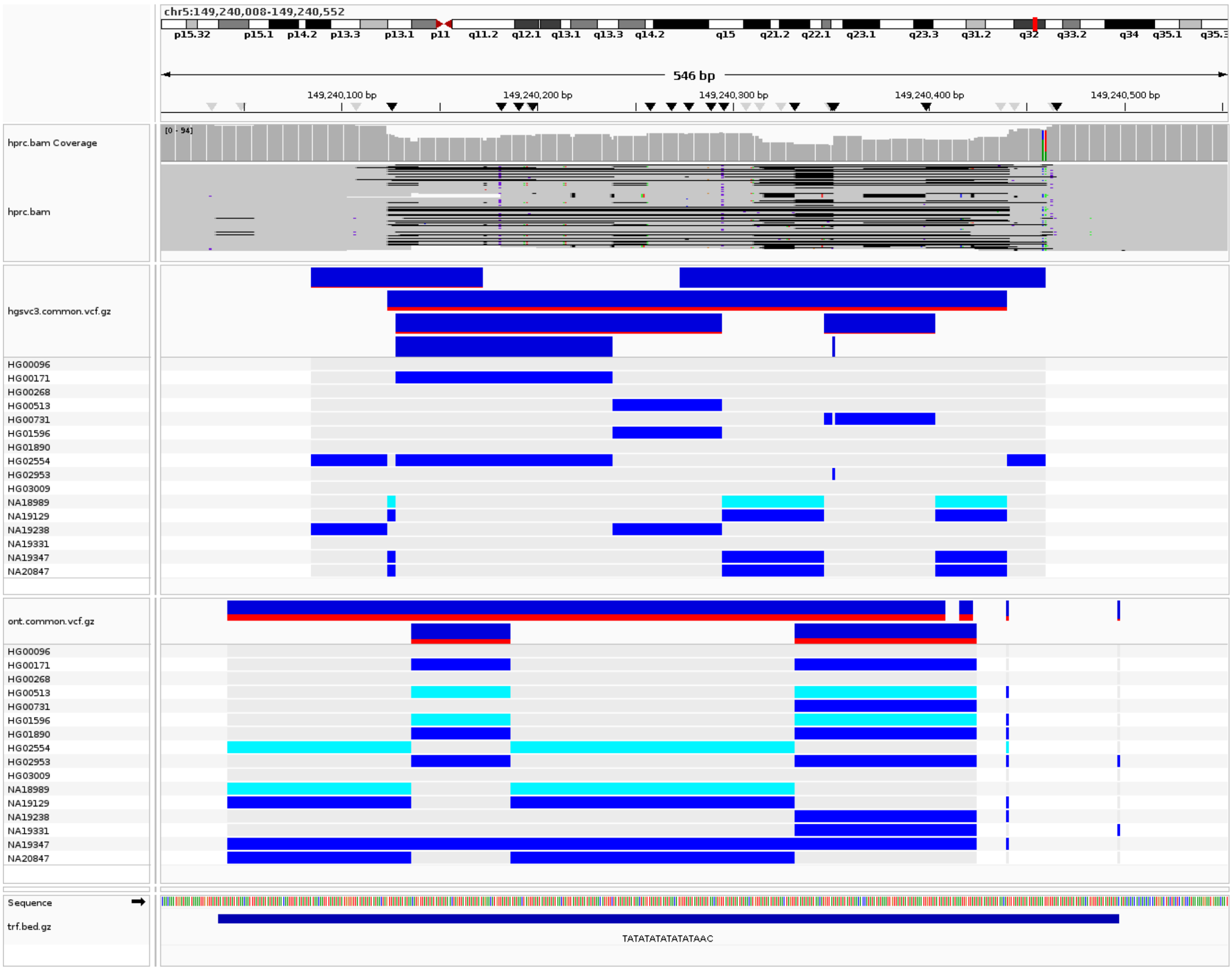

Supplementary Figure 12: Visualization of HPRC year-1 haplotype alignments (94 haplotypes from 47 samples) in IGV (squished view) for a tandem repeat locus identified by Tandem Repeat Finder (TRF) which is shown as a blue bar at the very bottom of the figure (panel trf.bed.gz). Each line in the upper panel represents an HPRC haplotype alignment and black lines show deletions whereas purple vertical lines show repeat insertions as point events. The coverage track at the top shows inconsistent alignment of deletions in HPRC haplotypes due to repeat microvariation and repeat unit insertions. Underneath in the middle panel are HGSVC3 SV calls with red representing the allele frequency (long bars - deletions, short bars - insertions as point events) followed by genotype calls (blue - heterozygous, turquoise - hom. alternative) in the 16 overlapping samples (**Fig. S7**). In the bottom panel are the SAGA SV calls followed by the genotype calls for the 16 overlapping samples. Overall, HGSVC3 reports 9 SV calls (7 in the 16 overlapping samples) from 63 high-quality assemblies compared to 6 SV calls reported by SAGA in 967 samples, highlighting the reduced allelic repeat variation present in the augmented graph at tandem repeat loci. Post genotyping this leads to tandem repeat variants represented as a mix of these graph repeat alleles, e.g., for HG00171, HGSVC3 reports a 112 bp deletion which is represented by SAGA as a 94 bp + 52 bp deletion and for NA18989, HGSVC3 reports a 318 bp deletion which is represented by SAGA as a 368 bp deletion followed by a 70 bp insertion. In a VCF-based SV comparison this may lead to large numbers of false negatives and false positives as SV size, type and location can differ.

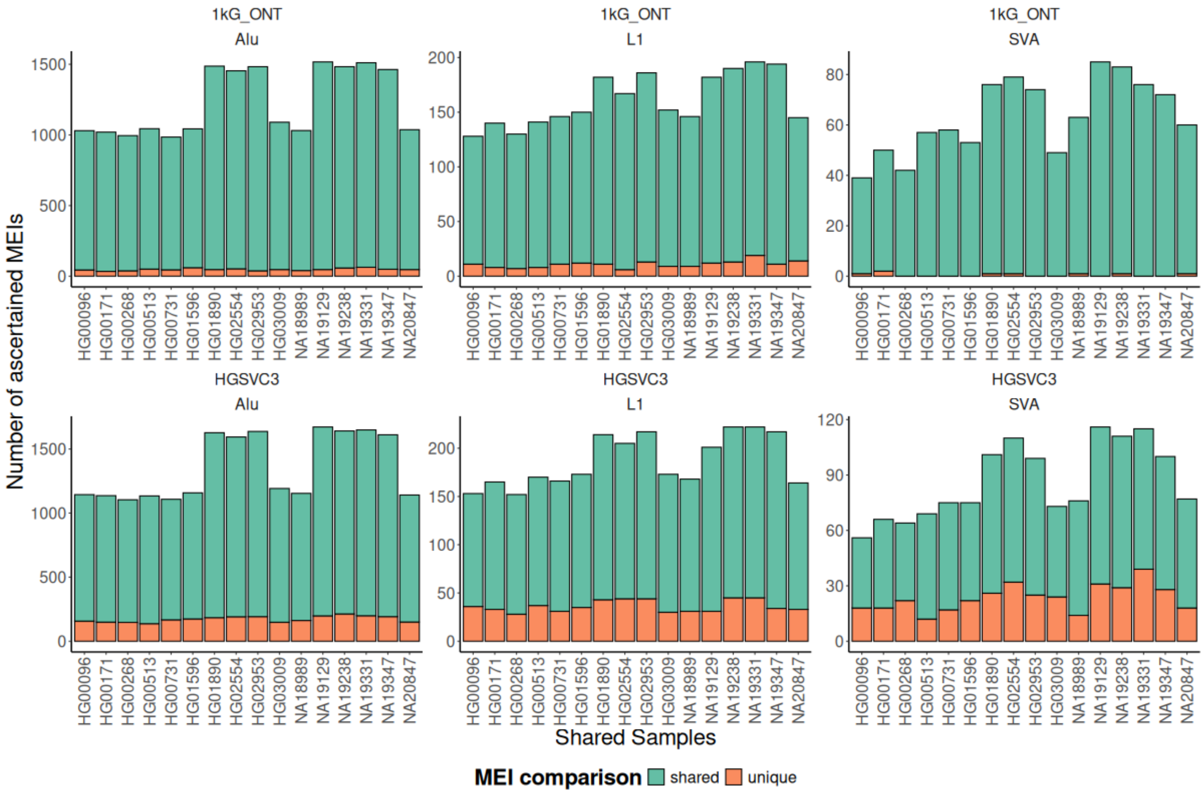

Supplementary Figure 13: Mobile element insertion calls of the SAGA framework compared to whole genome assemblies using canonical MEIs only. Using the MEI calls from multi-platform whole genome assemblies[14], we show the number of shared and unique MEIs by transposable element type for all canonical MEIs in the SAGA call set annotated with SVAN.

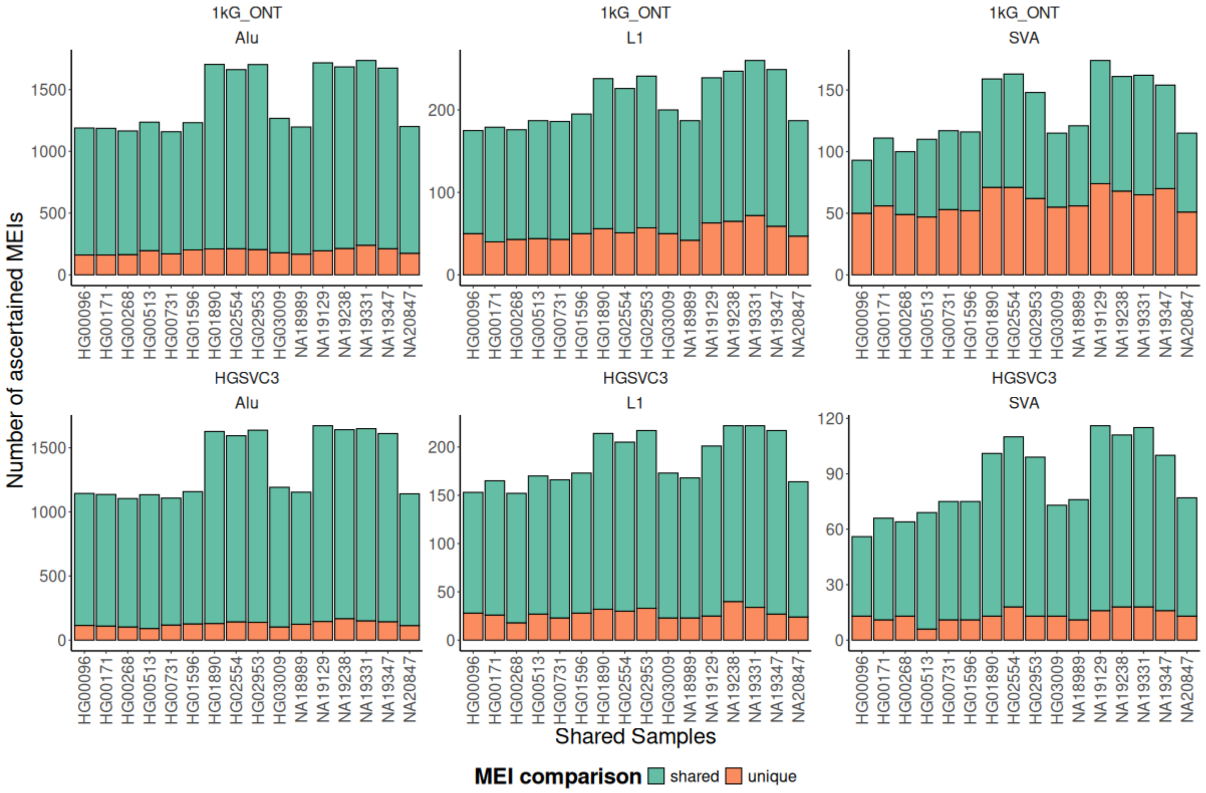

Supplementary Figure 14: Mobile element insertion calls of the SAGA framework compared to whole genome assemblies using non-canonical and canonical MEIs. Using the MEI calls from whole genome assemblies[14], we show the number of shared and unique MEIs by transposable element type for all non-canonical and canonical MEIs in the SAGA call set annotated with SVAN.

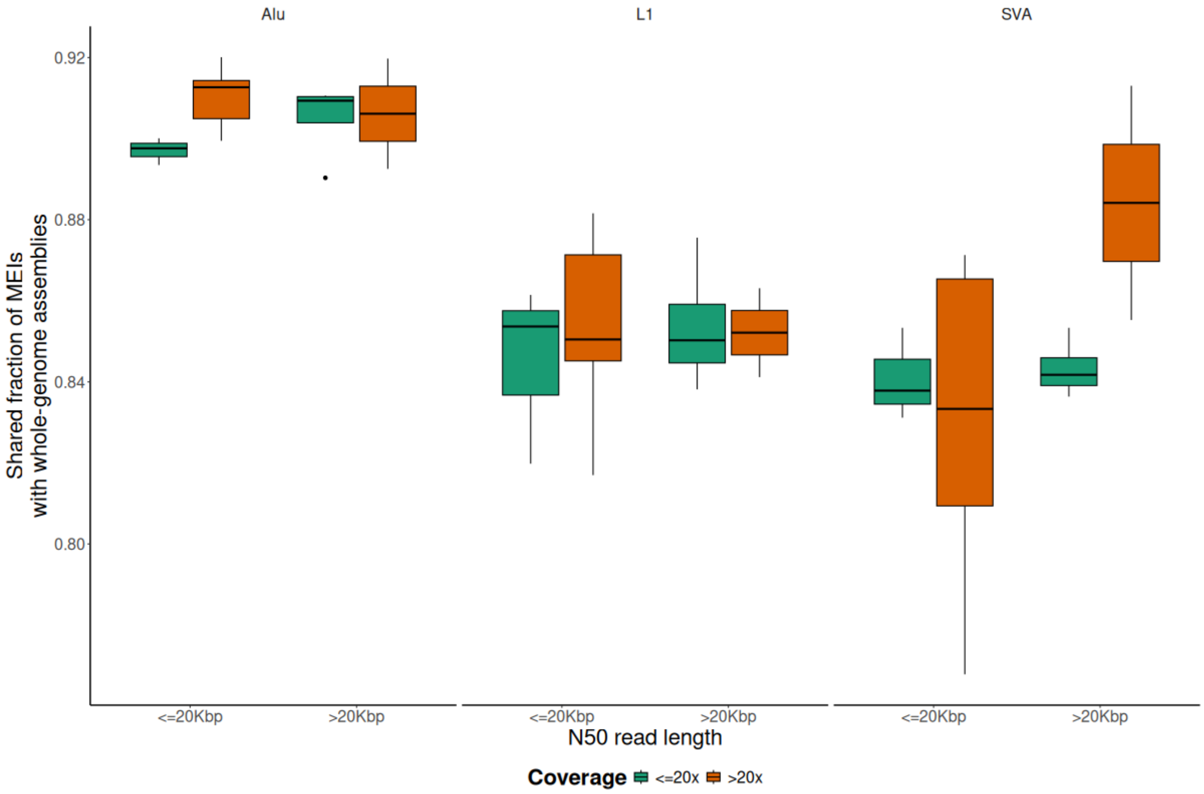

Supplementary Figure 15: Comparison of whole-genome based mobile element insertion calls stratified by N50 read length and coverage with respect to the calls made from our ONT 1kGP sample resource.

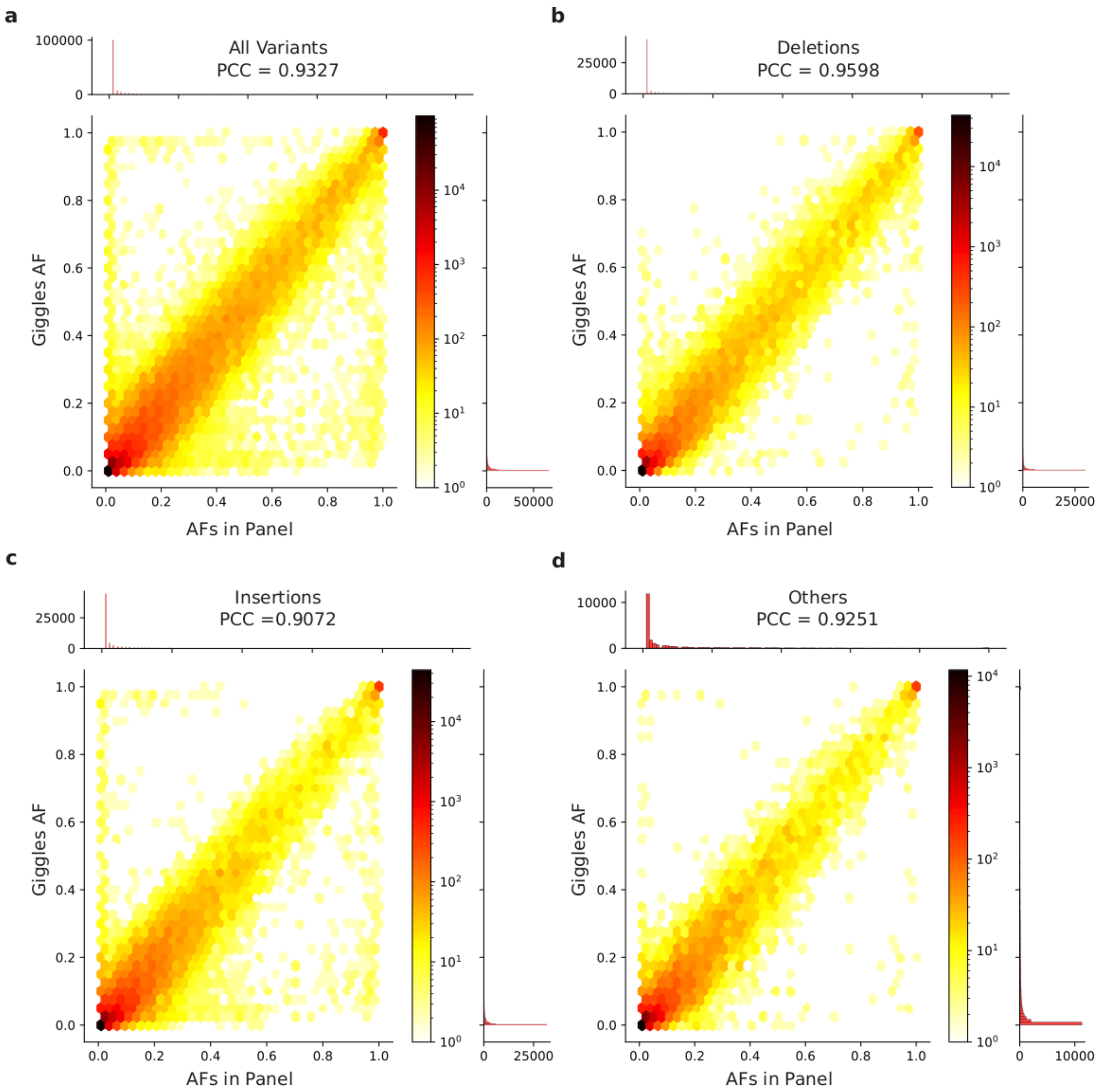

Supplementary Figure 16: The plot demonstrates the genotype quality of the genotypes by Giggles on the HPRC_mg_44+966 graph after filtering. Genotyping quality is shown here using a comparison of the allele frequency of an allele in the VCF panel (created using the HPRC[50] assemblies and the pseudo-haplotypes of the SAGA framework) with the allele frequency of the same allele genotyped by Giggles in the callset (using only the 908 unrelated samples from our callset). The plot has been broken into variant types: **a)** shows the plot for all variants, **b)** shows deletions, **c)** shows insertions and **d)** shows the rest of the variants which strictly do not fall into deletions or insertions. The Pearson's Correlation Coefficient (PCC) for each plot has been provided in the figure.

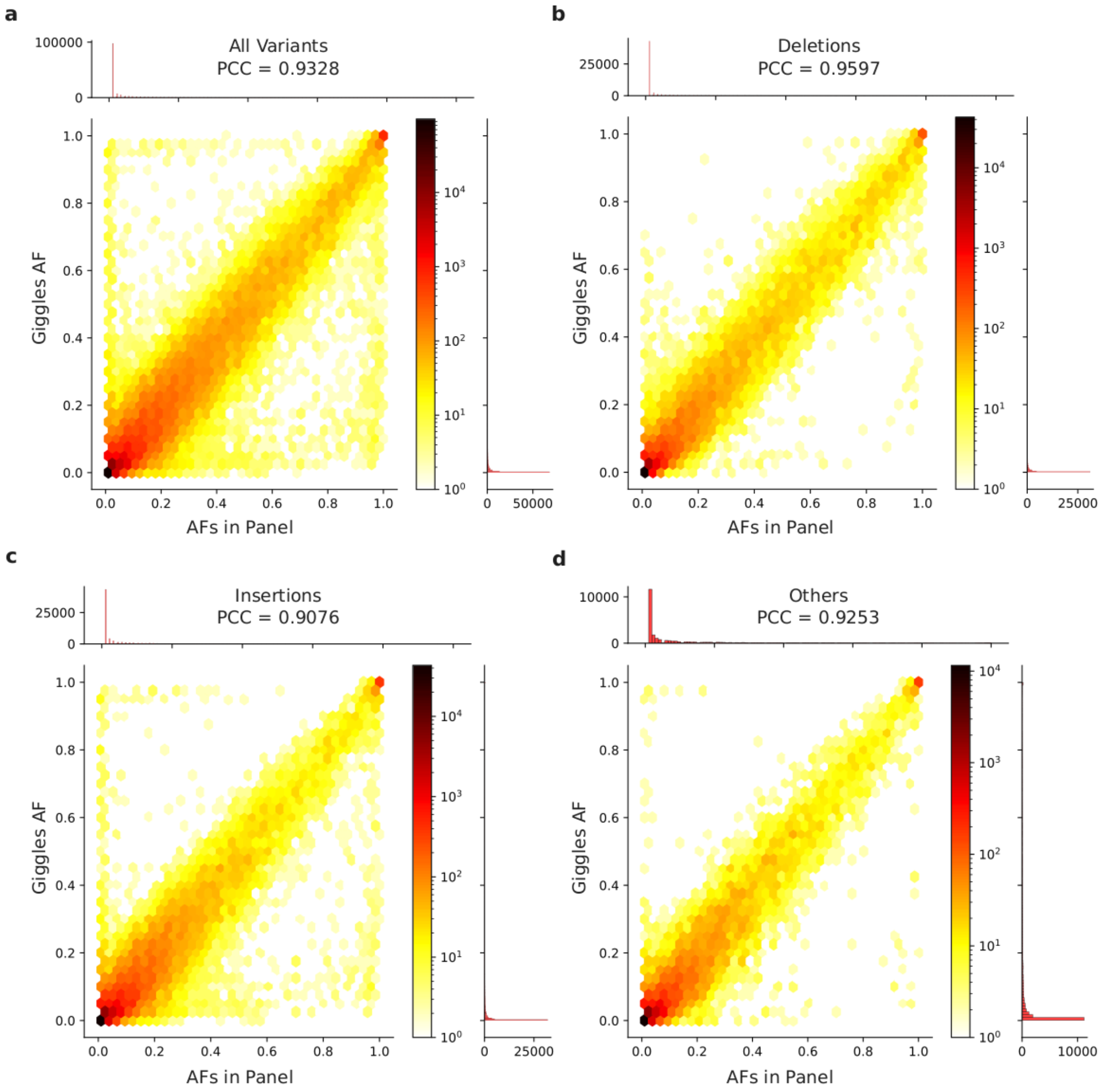

Supplementary Figure 17: The plot demonstrates the genotype quality of the genotypes by Giggles on the HPRC_mg_44+966 graph which have been phased using SHAPEIT5[51] and filtered. Genotyping quality is shown here using a comparison of the allele frequency of an allele in the VCF panel (created using the HPRC[50] assemblies and the pseudo-haplotypes of the SAGA framework) with the allele frequency of the same allele genotyped by Giggles in the callset (using only the 908 unrelated samples from our callset). The plot has been broken into variant types: **a)** shows the plot for all variants, **b)** shows deletions, **c)** shows insertions and **d)** shows the rest of the variants which strictly do not fall into deletions or insertions. The Pearson's Correlation Coefficient (PCC) for each plot has been provided in the figure.

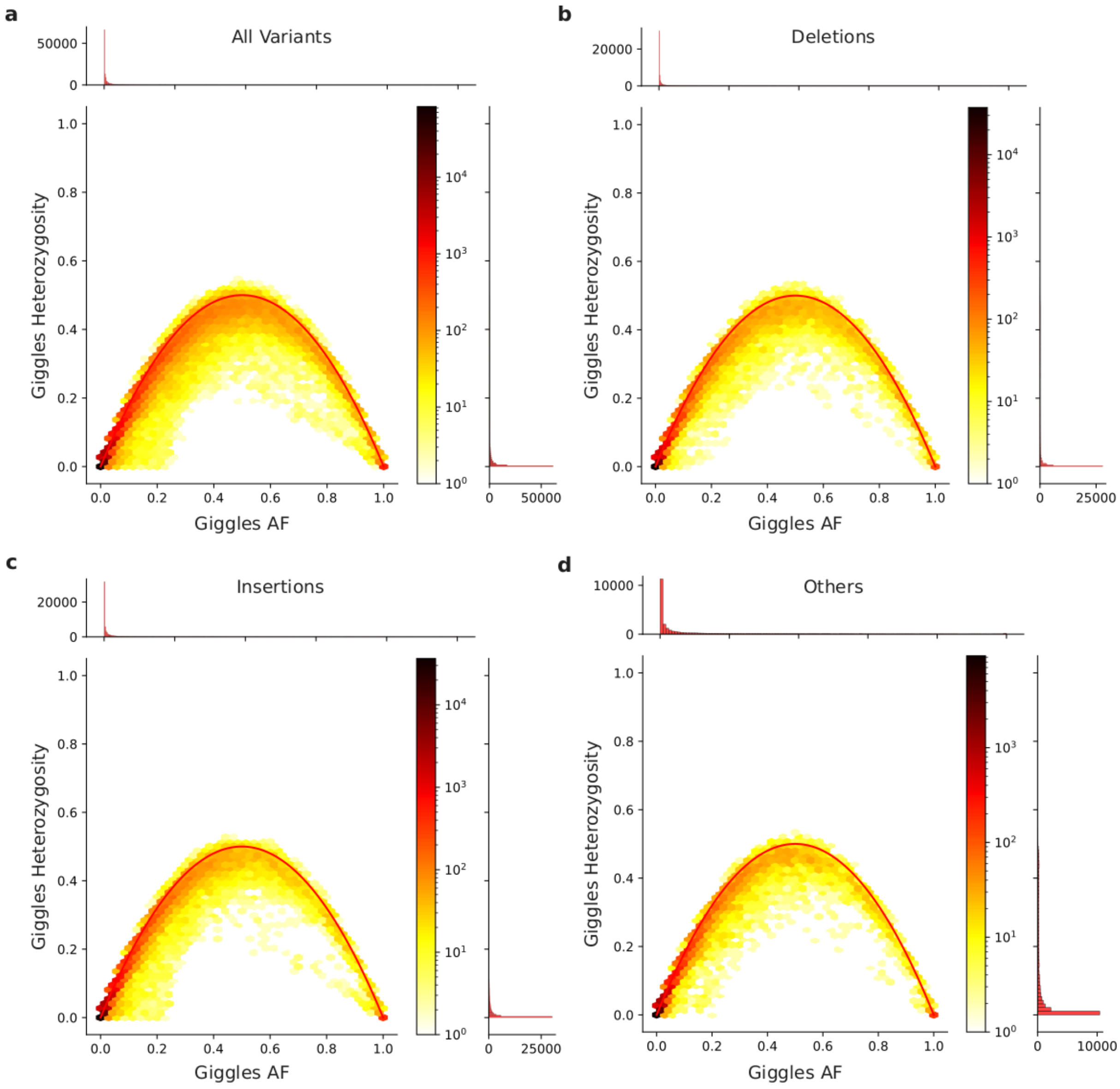

Supplementary Figure 18: The plot demonstrates the genotype quality of the genotypes by Giggles on the HPRC_mg_44+966 graph after filtering. Genotyping quality is shown here using a Hardy-Weinberg Equilibrium (HWE) plot given with the allele frequency of the genotyped allele and the percentage of samples heterozygous for that allele (using only the 908 unrelated samples from our callset). The plot has been broken into variant types: **a)** shows the HWE plot for all variants, **b)** shows deletions, **c)** shows insertions and **d)** shows the rest of the variants which strictly do not fall into deletions or insertions.

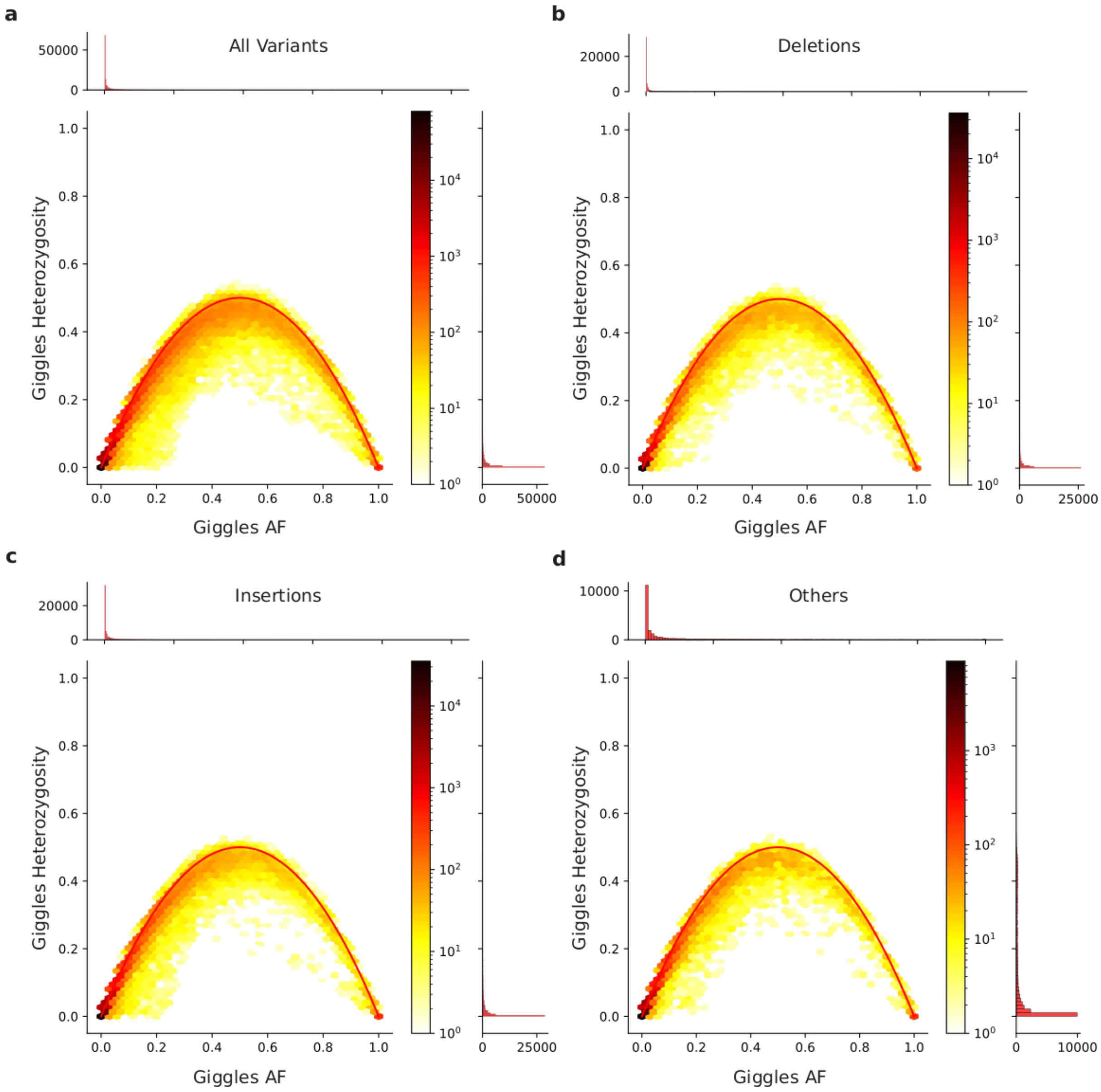

Supplementary Figure 19: The plot demonstrates the genotype quality of the genotypes by Giggles on the HPRC_mg_44+966 graph which have been phased using SHAPEIT5 and filtered. Genotyping quality is shown here using a Hardy-Weinberg Equilibrium (HWE) plot given with the allele frequency of the genotyped allele and the percentage of samples heterozygous for that allele (using only the 908 unrelated samples from our callset). The plot has been broken into variant types: **a)** shows the HWE plot for all variants, **b)** shows deletions, **c)** shows insertions and **d)** shows the rest of the variants which strictly do not fall into deletions or insertions.

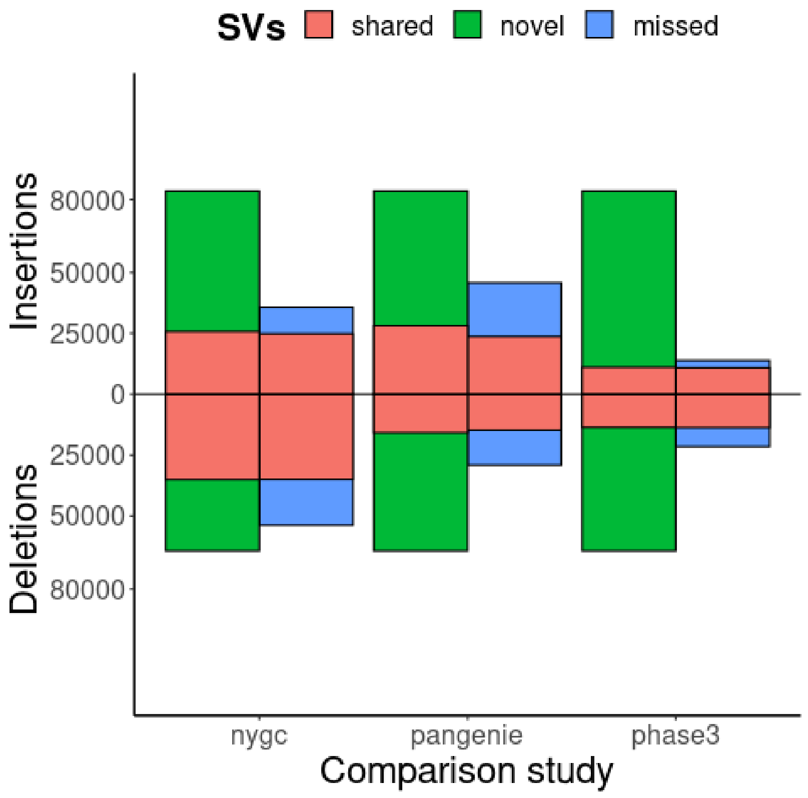

Supplementary Figure 20: The number of shared (red), novel (green) and missed (blue) SVs of our study (left bar) compared to prior SV studies (right bar) subsetted to samples present in our cohort. Comparison studies include deep-coverage short-read data generated by the New York Genome Center[2] (nygc), long-read data analyzed by the Human Genome Structural Variation Consortium project[33] and genotyped in the NYGC data using pangenie[52] (pangenie) and the 1kGP phase 3 SV callset[3] (phase3).

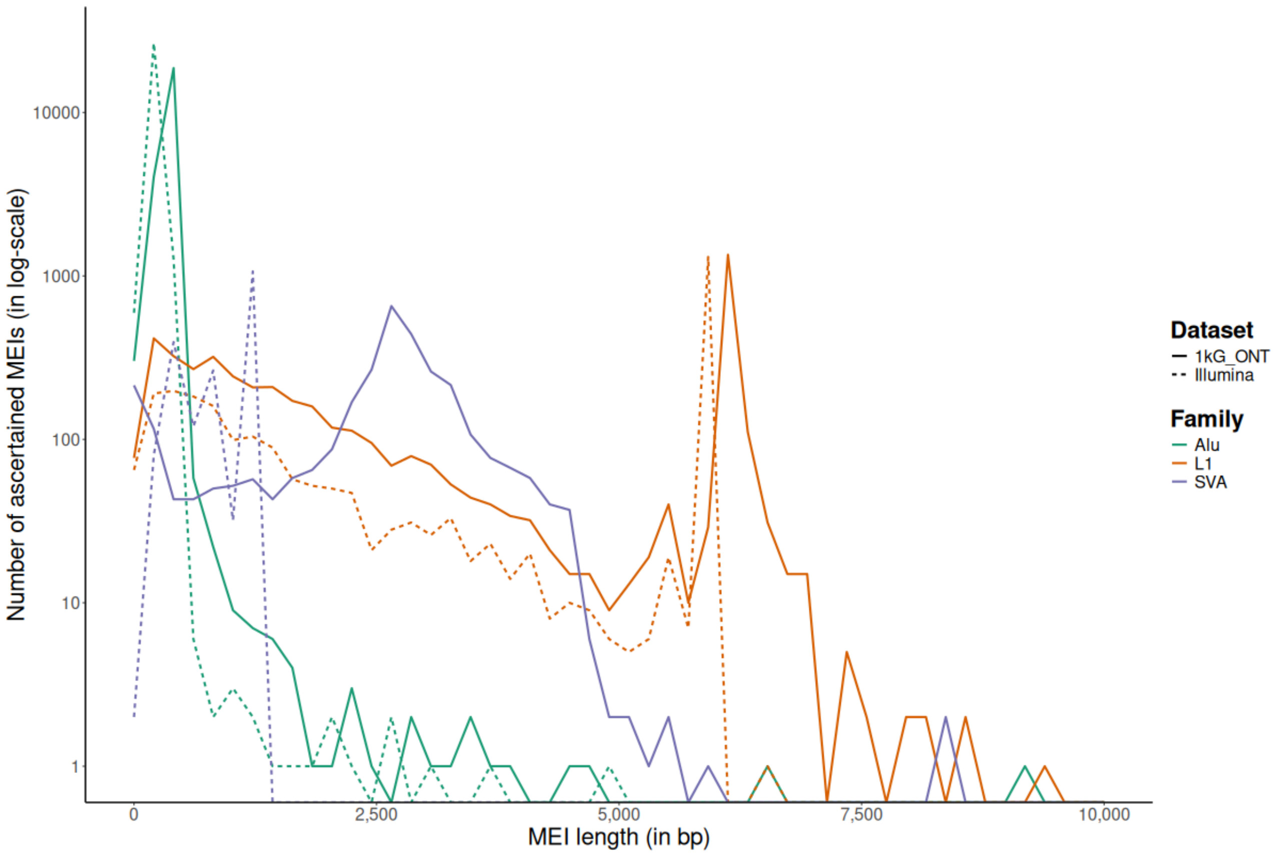

Supplementary Figure 21: Comparison of the SV length of short-read derived MEI predictions[2] and MEIs in the sequence-resolved SAGA call set.

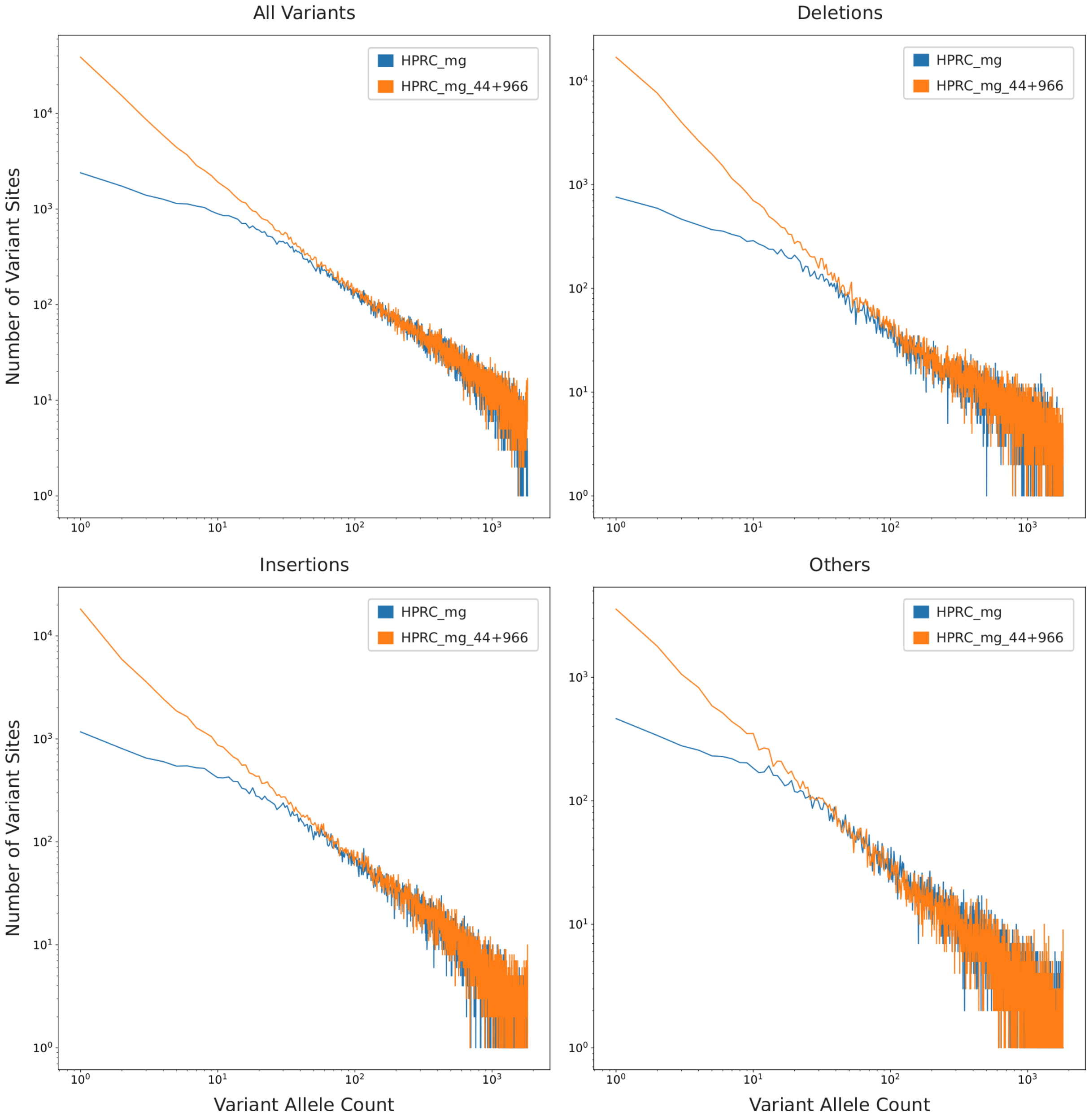

Supplementary Figure 22: The figure shows the relation between Variant Allele Count and the Number of Variant Sites with that allele count in the logarithmic space. In blue, the filtered genotypes of the Giggles callset on the HPRC_mg graph is shown and orange shows the final phased callset, which is the Giggles genotyped callset on the HPRC_mg_44+966 subsequently phased with SHAPEIT5[51]. The plot has been broken into variant types: **a)** shows the plot for all variants, **b)** shows deletions, **c)** shows insertions and **d)** shows the rest of the variants which strictly do not fall into deletions or insertions.

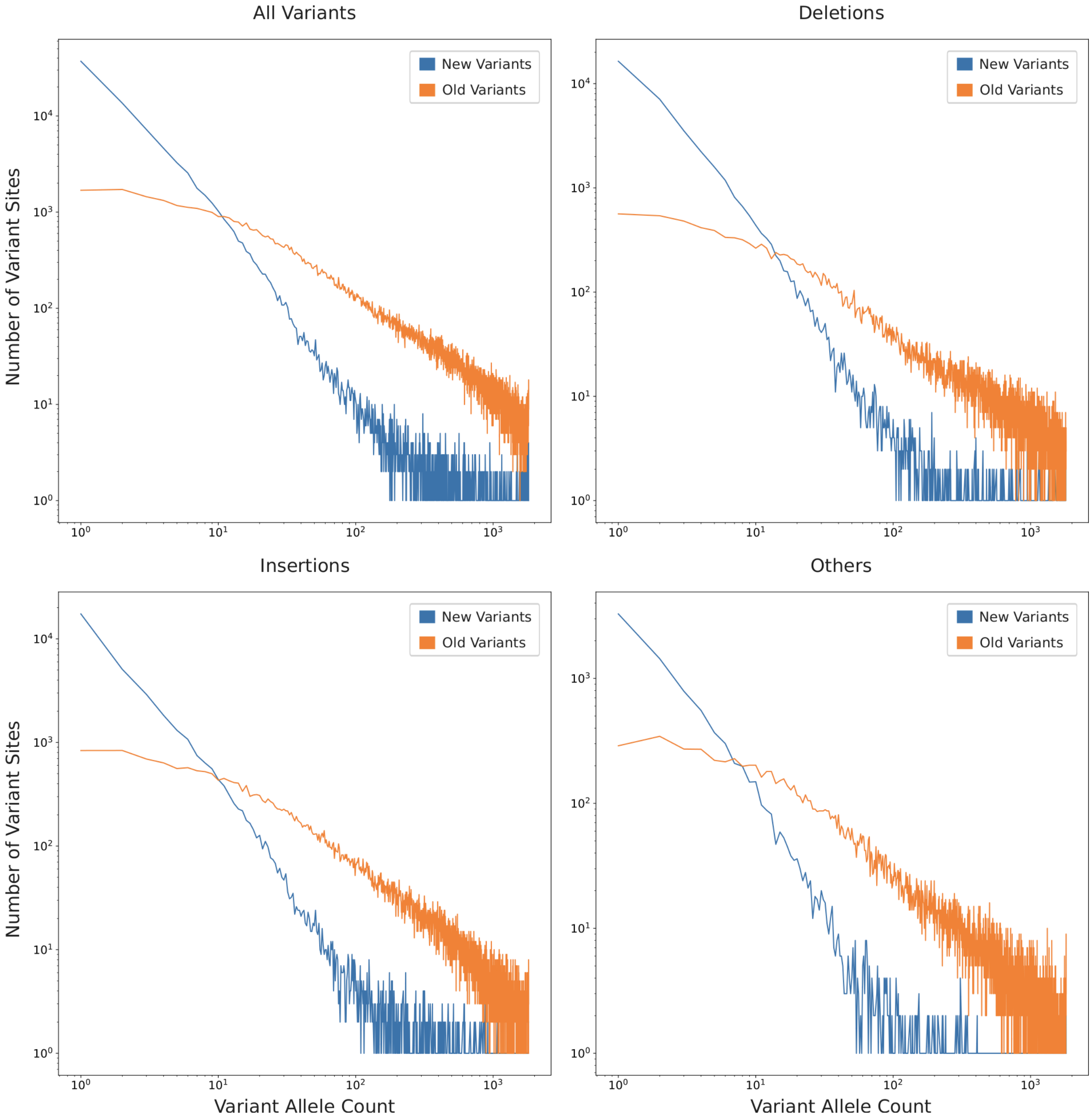

Supplementary Figure 23: The figure shows the relation between Variant Allele Count and the Number of Variant Sites with that allele count in the logarithmic space. The data shown here are the genotypes of the final phased callset, which is the Giggles genotyped callset on the HPRC_mg_44+966 subsequently phased with SHAPEIT5[51]. The blue line is the new variants, determined by alleles exclusively found in the pseudo-haplotypes and the orange line is the old variants, determined by alleles exclusively found in the HPRC[50] assemblies. The plot has been broken into variant types: **a)** shows the plot for all variants, **b)** shows deletions, **c)** shows insertions and **d)** shows the rest of the variants which strictly do not fall into deletions or insertions.

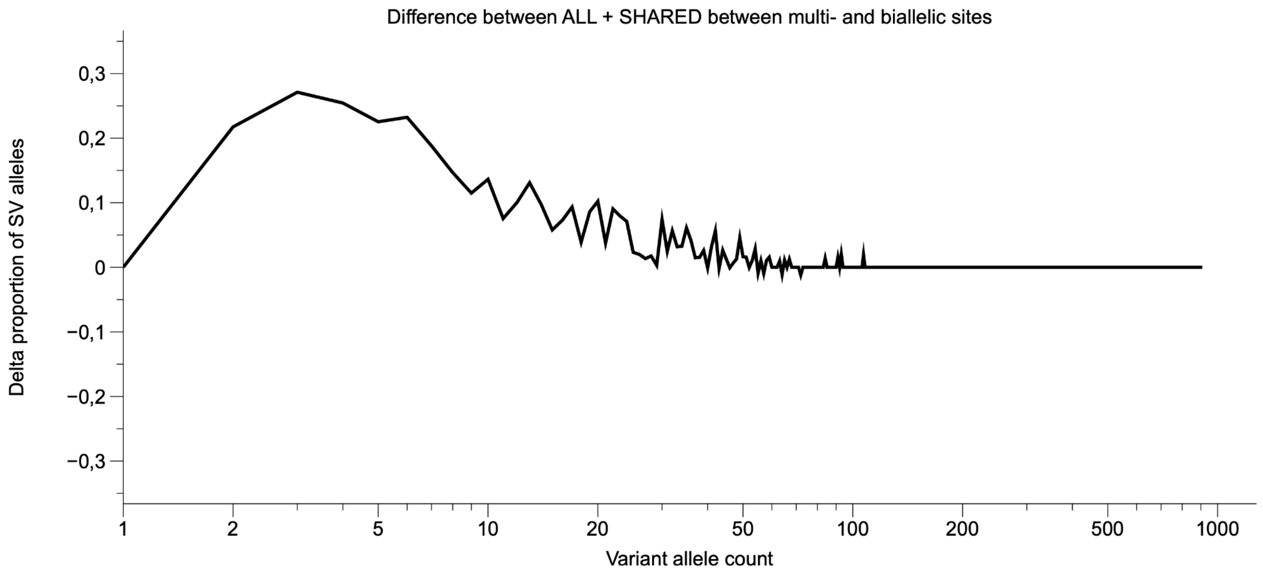

Supplementary Figure 24: Difference between the fraction of SVs shared across at least two continental populations (ALL = shared across all continental populations, SHARED = shared across at least two but not all continental populations) for multiallelic over biallelic SV sites. Multiallelic SVs have a higher propensity to be shared across continental ancestries than biallelic SVs, including for low frequency multi-allelic alleles.

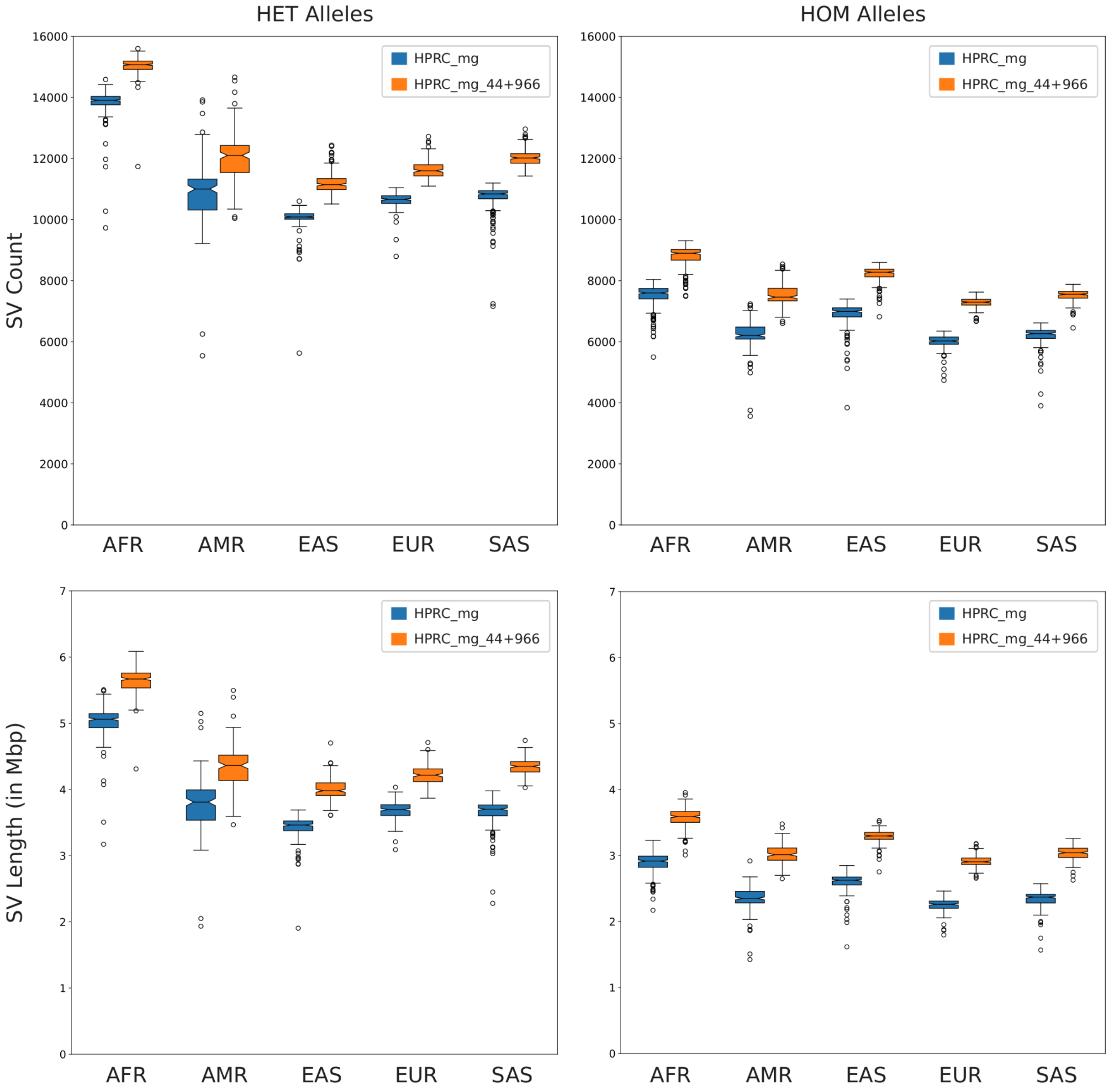

Supplementary Figure 25: The plot shows the distribution of SV counts for each sample (top row) and the cumulative length of the present SVs in the sample (bottom row). The variants have been divided into HETs and HOMs to denote heterozygous alleles (left column) and homozygous alternate alleles (right column). In blue, the filtered genotypes of the Giggles callset on the HPRC_mg graph is shown and orange shows the final phased callset, which is the Giggles genotyped callset on the HPRC_mg_44+966 subsequently phased with SHAPEIT5[51].

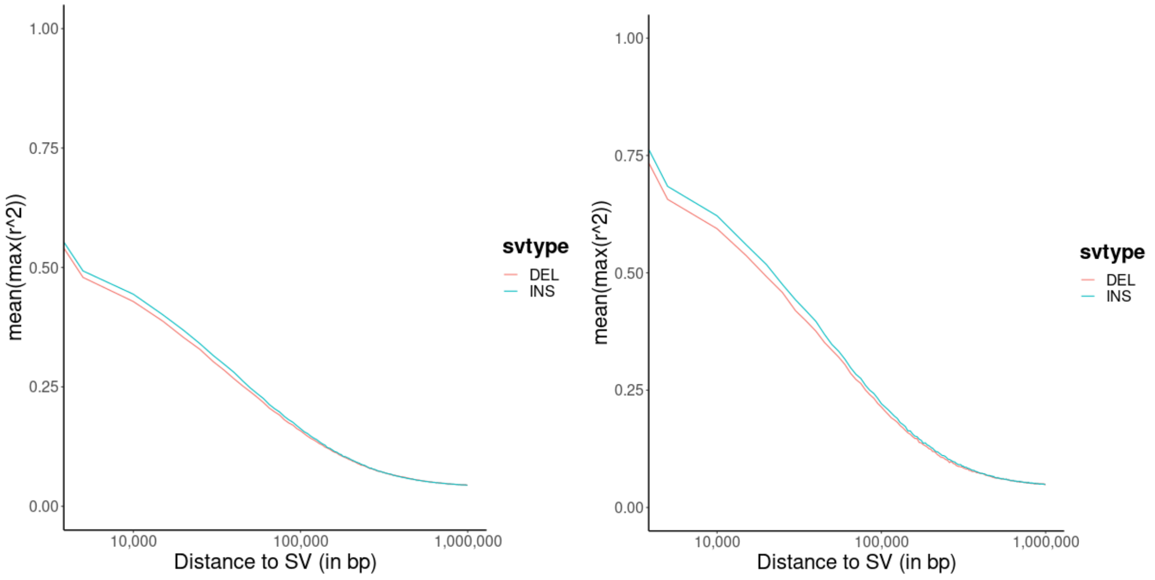

Supplementary Figure 26: Linkage disequilibrium (LD) of SVs (MAF >= 1%) with nearby single nucleotide polymorphisms (SNPs) as a function of the distance of the SV to the SNP. The left panel shows all SVs whereas the right panel shows the subset of SVs in Genome in a Bottle high-confident regions of the CHM13 genome (2.3Gbp, 74.2%).

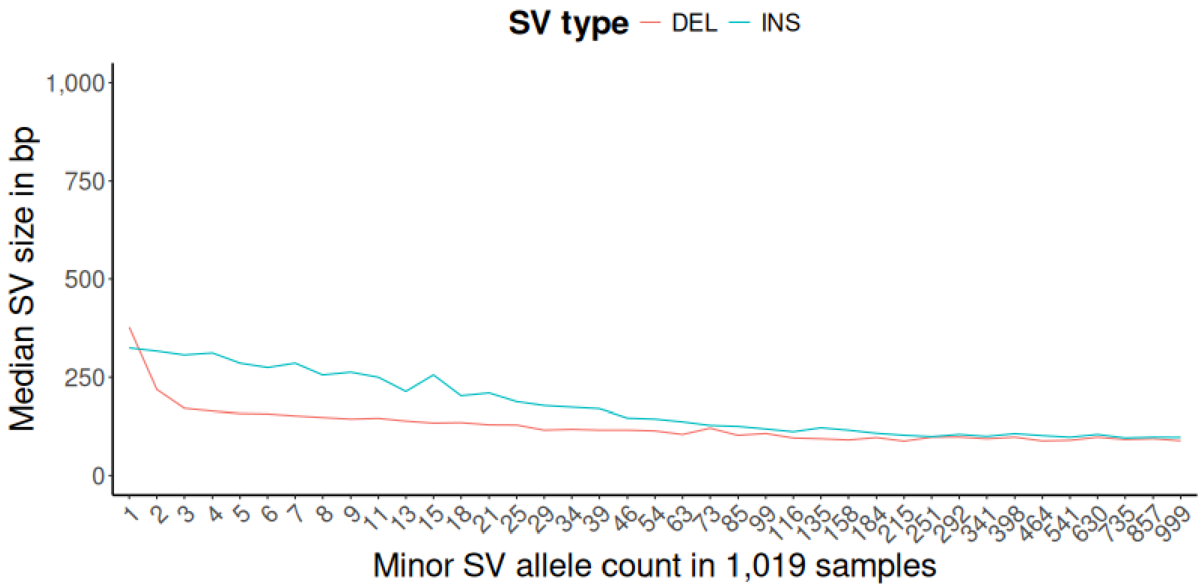

Supplementary Figure 27: Median SV size by minor SV allele count. The x-axis value indicates the upper bound of the minor allele count bin. The first bin represents all SVs of allele count 1, the second bin all SVs of allele count greater than 1 and smaller or equal to 2 and so on for increasing allele counts (SVs discovered as deletions are depicted in red, and insertions discovered as insertions are depicted in blue). We conclude from this figure that larger SVs, irrespective of being insertions or deletions, are significantly rarer in the population than smaller SVs, a trend previously detected only for deletions[3].

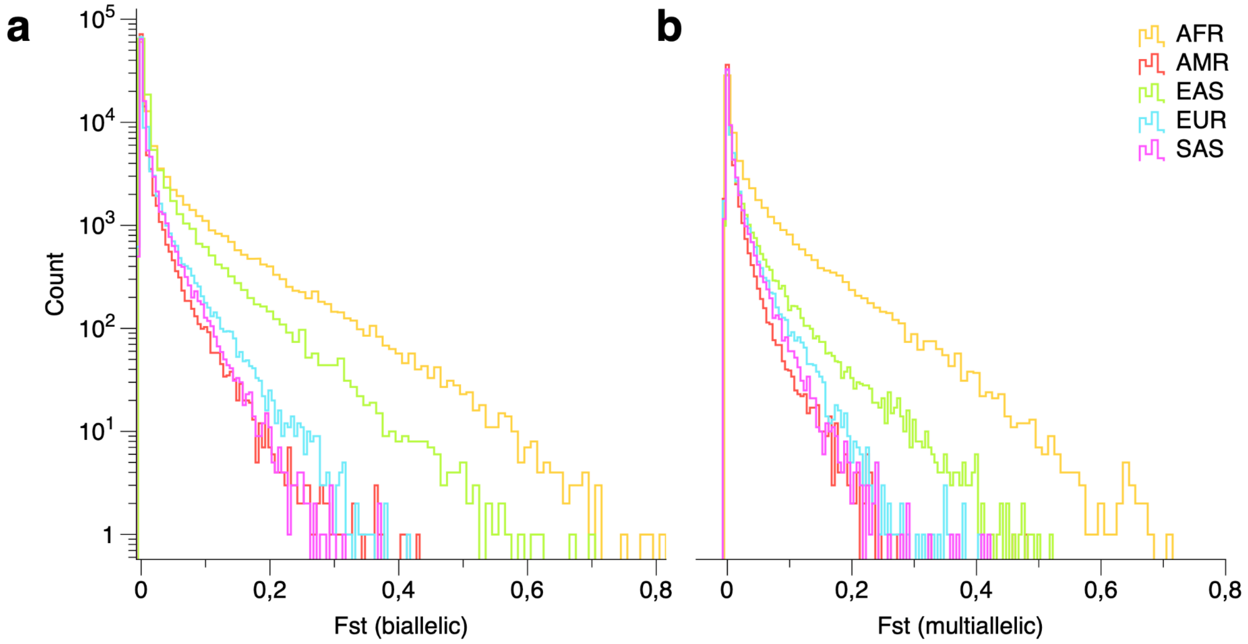

Supplementary Figure 28: Histogram of **a)** biallelic SV Fst values and **b)** multiallelic SV Fst values for all five continental populations.

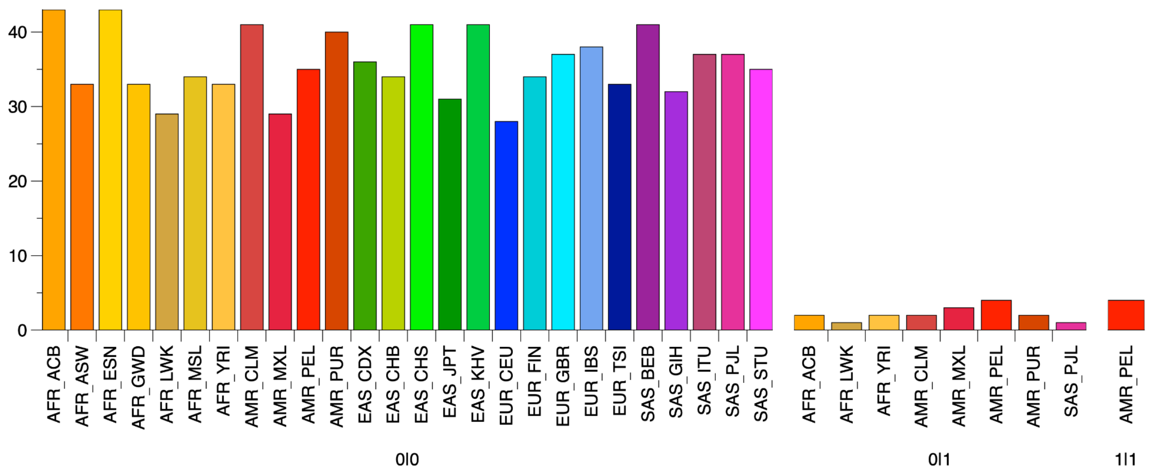

Supplementary Figure 29: Occurrences of SV *chr7-109295061-COMPLEX->s317119<s345452>s317121-246* (Fst 0.41) near *LAMB1* in the populations. The AMR populations in general show an enrichment of heterozygous copies. With homozygous copies occurring in PEL exclusively.

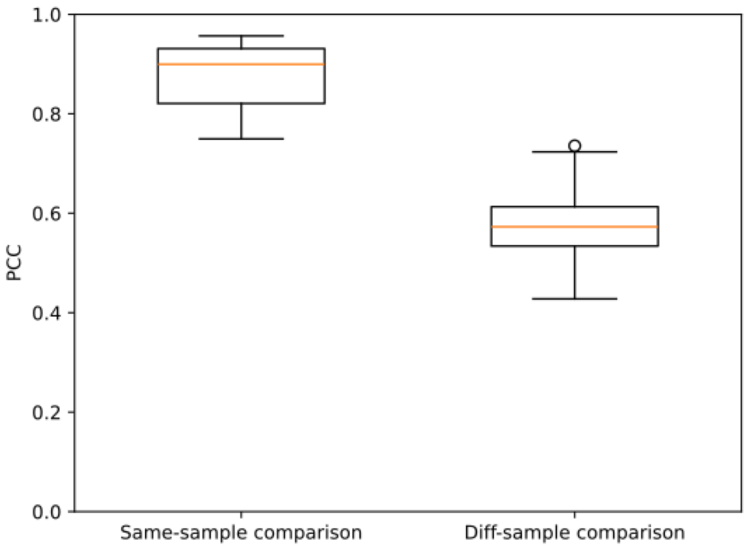

Supplementary Figure 30: The Pearson's Correlation Coefficient (PCC) of comparing vamos-based VNTR calls generated from our long-read 1kGP resource to VNTRs called from multi-platform whole genomes assemblies[14] (HGSVC3) using vamos (see **Note S5** 'Calling VNTRs using vamos'). We compared the count of repeat units in the VNTR alleles obtained from the 1kGP ONT reads to the alleles obtained from HGSVC3 assemblies. We filtered out the VNTR alleles where the HGSVC3 assemblies reported alleles close in length to the reference VNTR allele. We determined the alleles as close in length to the reference when the length of the HGSVC allele was within 90% to 110% of the reference allele.

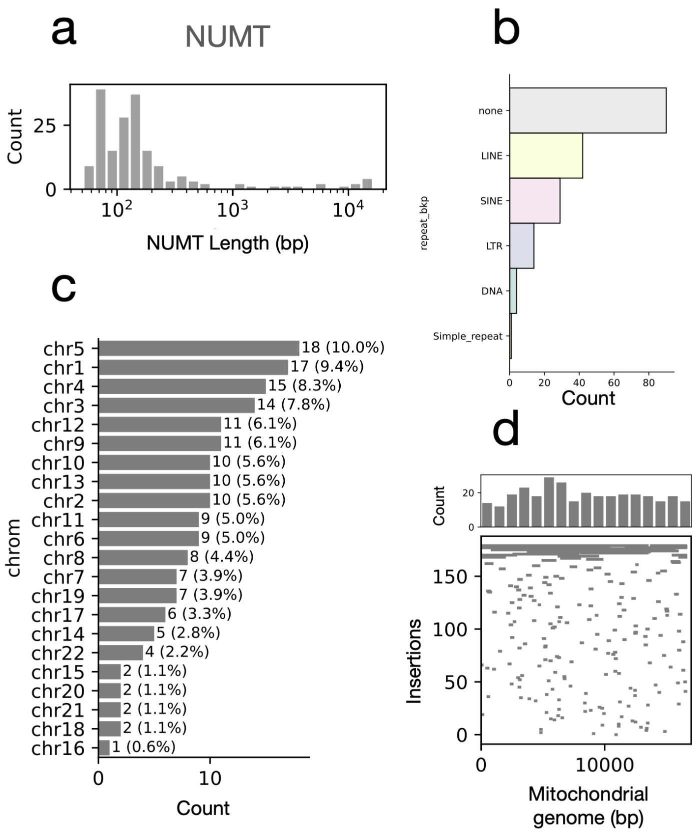

Supplementary Figure 31: a) Length distribution of Nuclear mitochondrial DNA (NUMT) elements. **b)** Number of NUMTs with insertion breakpoints that overlap each repeat family in the reference. **c)** Distribution of NUMTs per chromosome. **d)** Stacked dot-plots summarizing the alignments of each insertion/deletion against the mitochondrial genome (bottom) and histogram of alignment coverage (up).

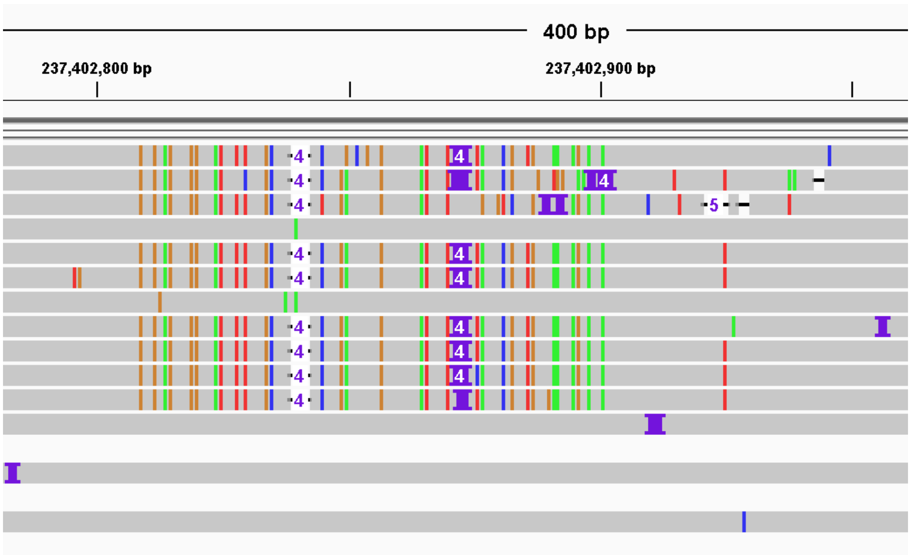

Supplementary Figure 32: Heterozygous inversion of length ~110 bp on chr1 in sample HG00107. Mapping with minimap2 to GRCh38 results in a forced high-mismatch alignment through the inversion.

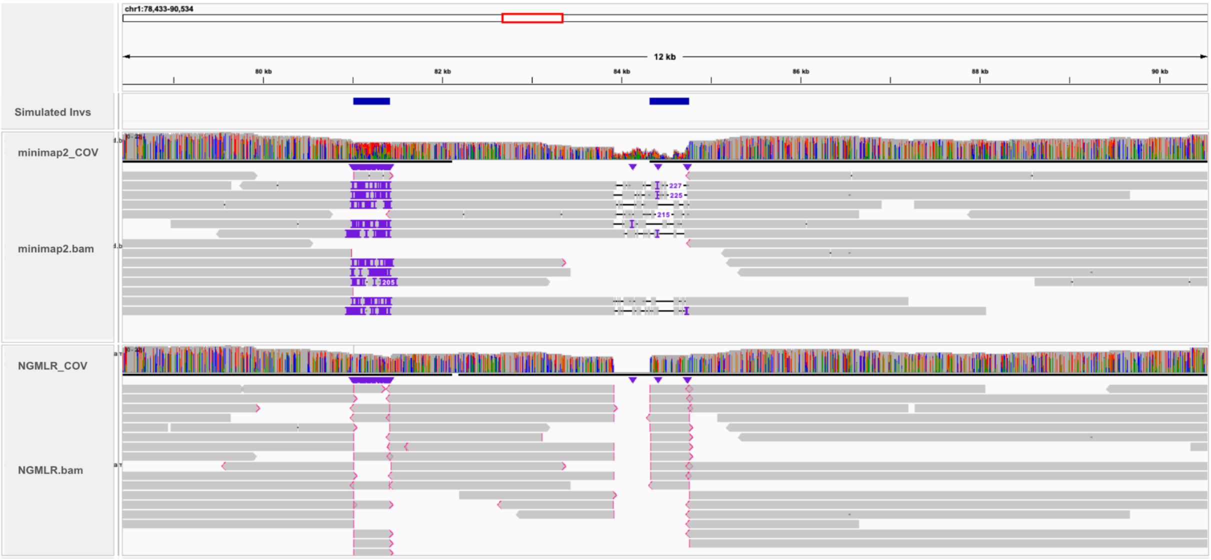

Supplementary Figure 33: Comparative Analysis of NGMLR and minimap2 in small inversion detection. Exemplary alignment outcomes by minimap2 and NGMLR within regions containing small inversions, approximately 500 base pairs each. It illustrates cases of a distinct inversion and an inversion adjacent to a small deletion. NGMLR demonstrates precise alignment in both instances, while minimap2 is characterized by the insertion of gaps and mismatches, thereby highlighting the superior performance of NGMLR in accurately aligning small inversions.

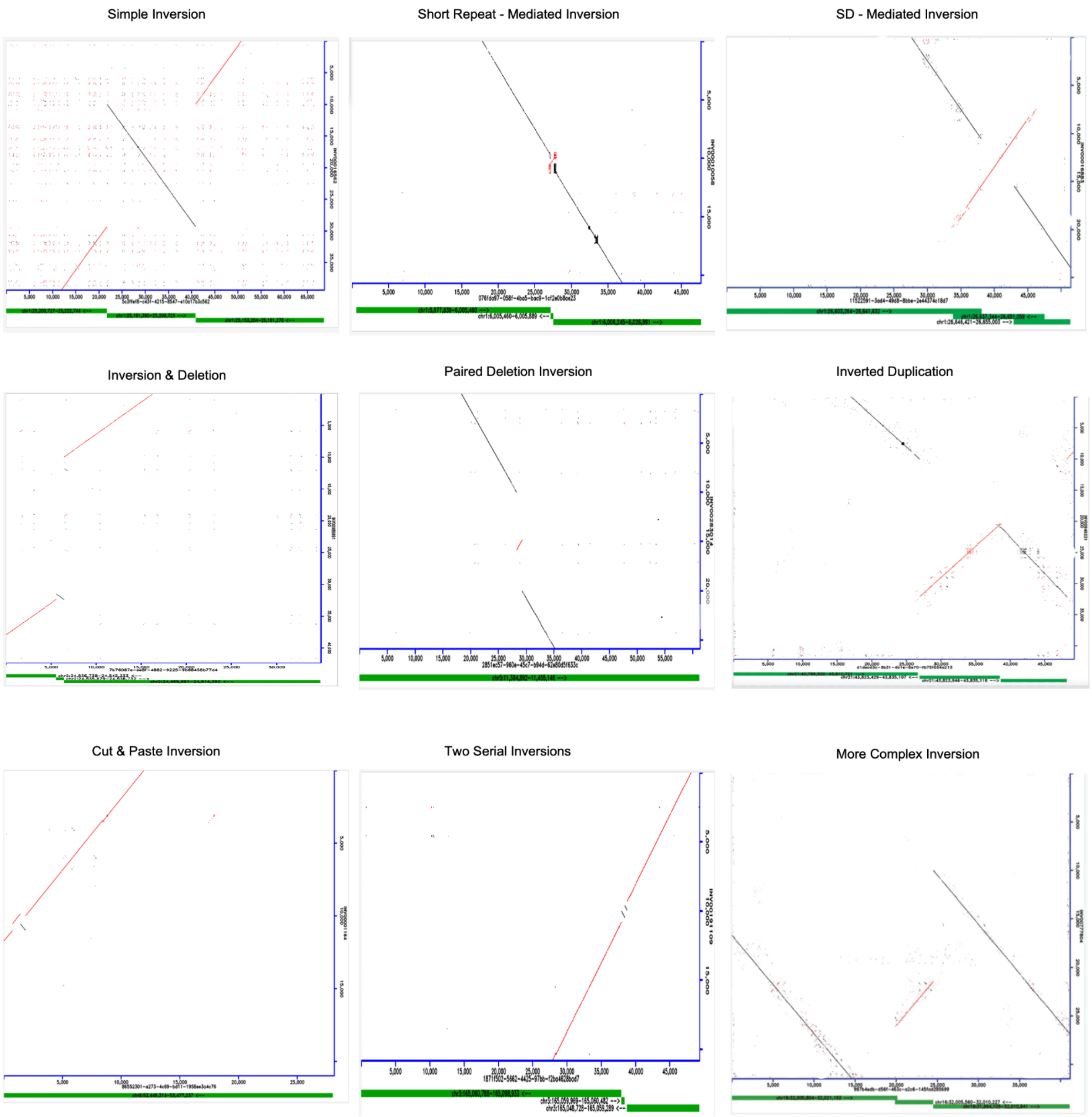

Supplementary Figure 34: Representative dot plots illustrating the categorization of inversions extracted from real sample data using wally[53]. The x-axis represents an ONT read in one of the samples in our resource for a specified genomic location, while the y-axis represents the corresponding region in the hg38 reference genome. Each candidate inversion locus was manually examined through dot plot analysis, facilitating the validation of candidate inversion locations and their classification into one of the eight distinct categories showcased in the graph.

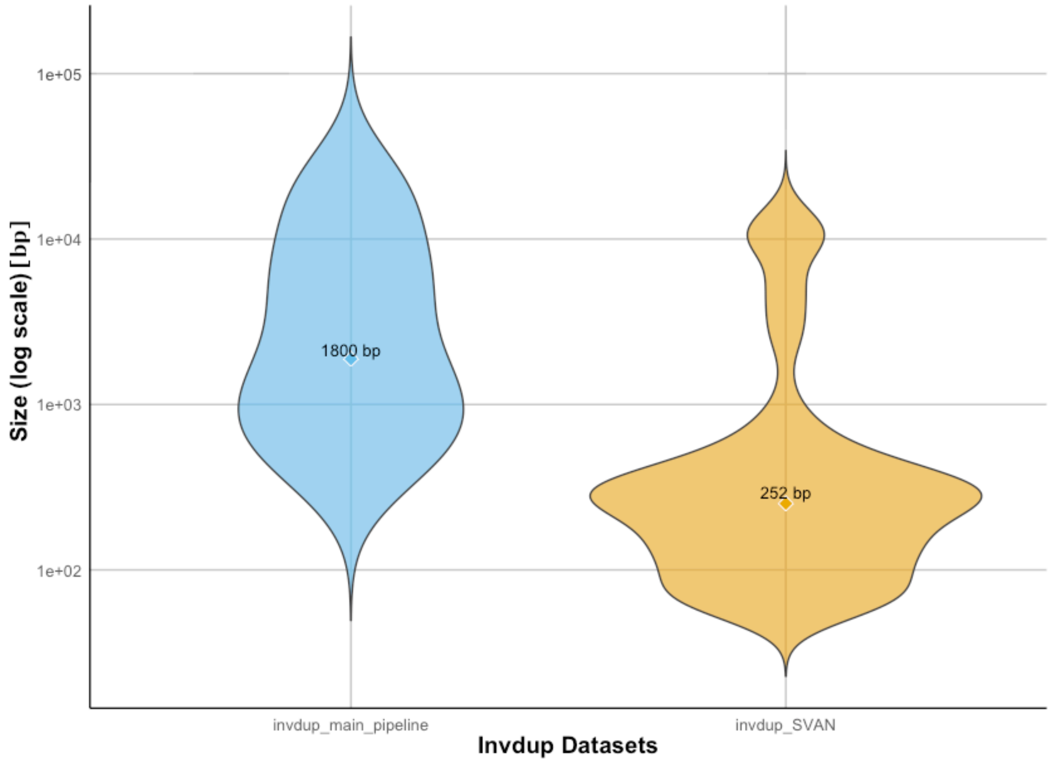

Supplementary Figure 35: Violin plots comparing the size distribution of inverted duplications detected through the main inversion detection pipeline versus SVAN. Calls are depicted on the x-axis, size of the detected inversions on the y-axis. The median size of inverted duplications from the main pipeline (1.8 kb), represented in blue, is higher that of those detected by SVAN (252 bp), illustrated in orange. It should be noted that inverted duplications identified through SVAN exhibit a 13% false positive rate in the current dataset based on manual inspection.

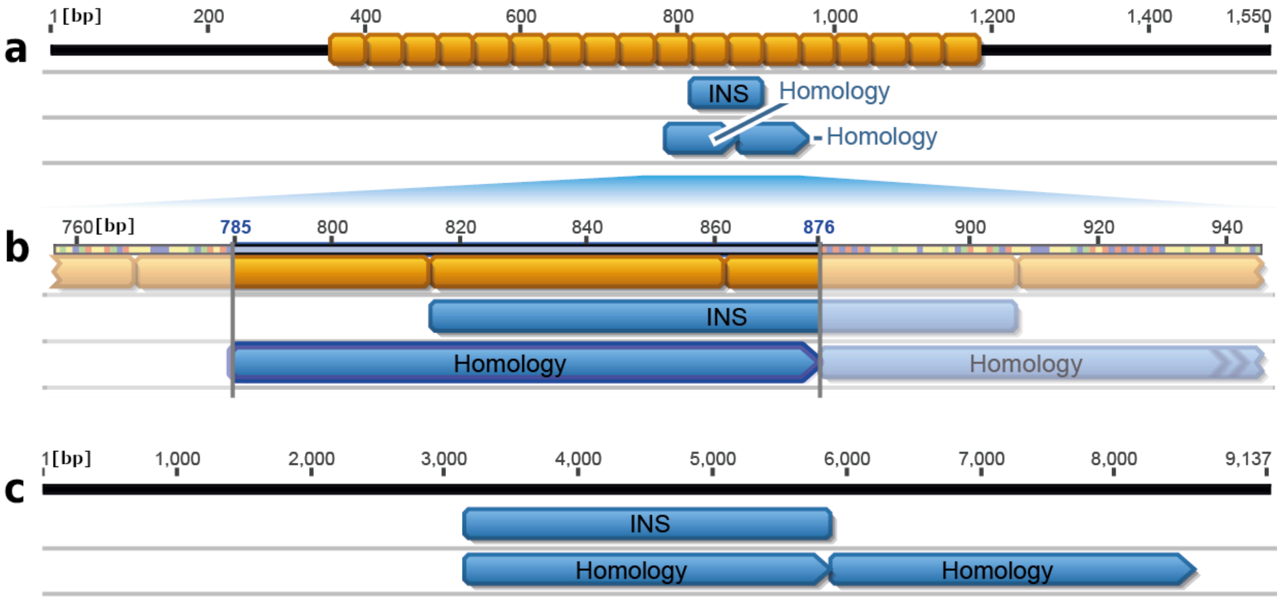

Supplementary Figure 36: 92 bp VNTR insertion and 2.7 kb tandem duplication with detected homologies. The top row in each panel shows a region of the CHM13 reference with the insertion sequence implanted. **a**) A 92 bp insertion (INS, turquoise) is inserted into a repeat (orange boxes represent one repeat unit). **b**) Detailed view of a): The insertion contains two repeat units and the detected homologous flanking sequences (Homology, turquoise) encompass a total of two repeat units as well. **c)** Tandem duplication of a 2.7 kb sequence results in the detection of 2.7 kb long homologous stretches spanning the insertion (left) and the duplicated segment (right).

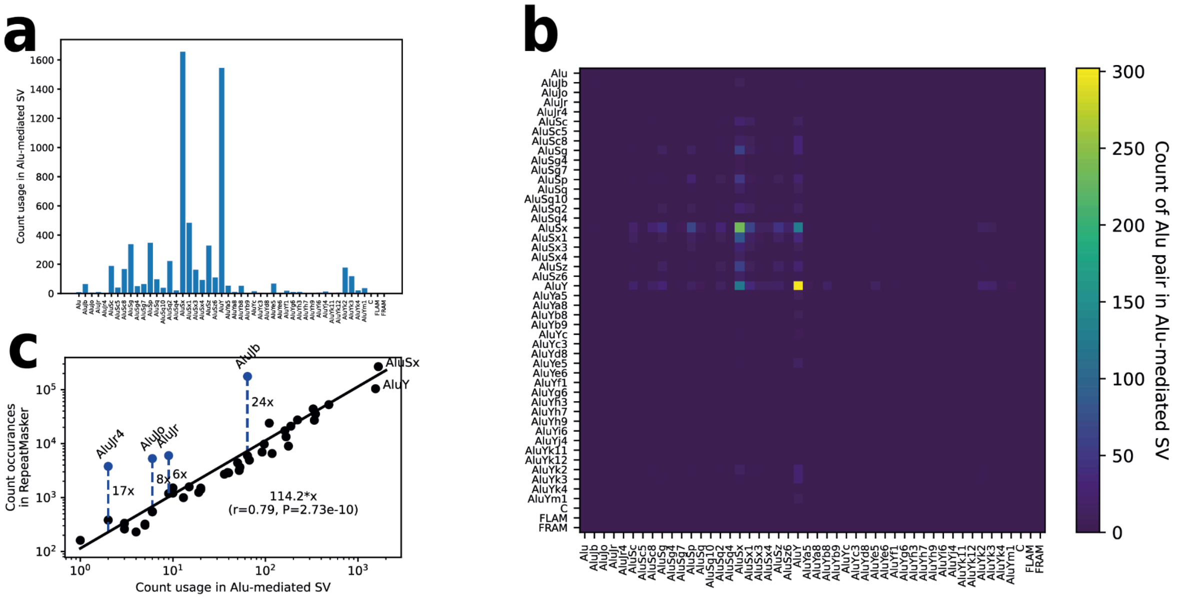

Supplementary Figure 37: Alu element usage in Alu-mediated SV. **a)** Counts of individual Alu-elements at breakpoint flanks. **b)** Heatmap showing the abundance of different Alu element pairs at the flanks of Alu-mediated SV. AluY–AluY pairs (9.0%) followed by AluSx-AluSx pairs (7.0%) represent the most common configurations. **c)** Scatter plot showing the relationship between the number of Alu elements at the flanks of Alu-mediated SV and inside the CHM13 RepeatMasker track. Members of the AluJ family are colored blue. Fold-change between AluJ occurrences in the CHM13 reference and their expected

contribution to Alu-mediated SV (as predicted by the linear regression) is shown in labeled, dashed blue lines.

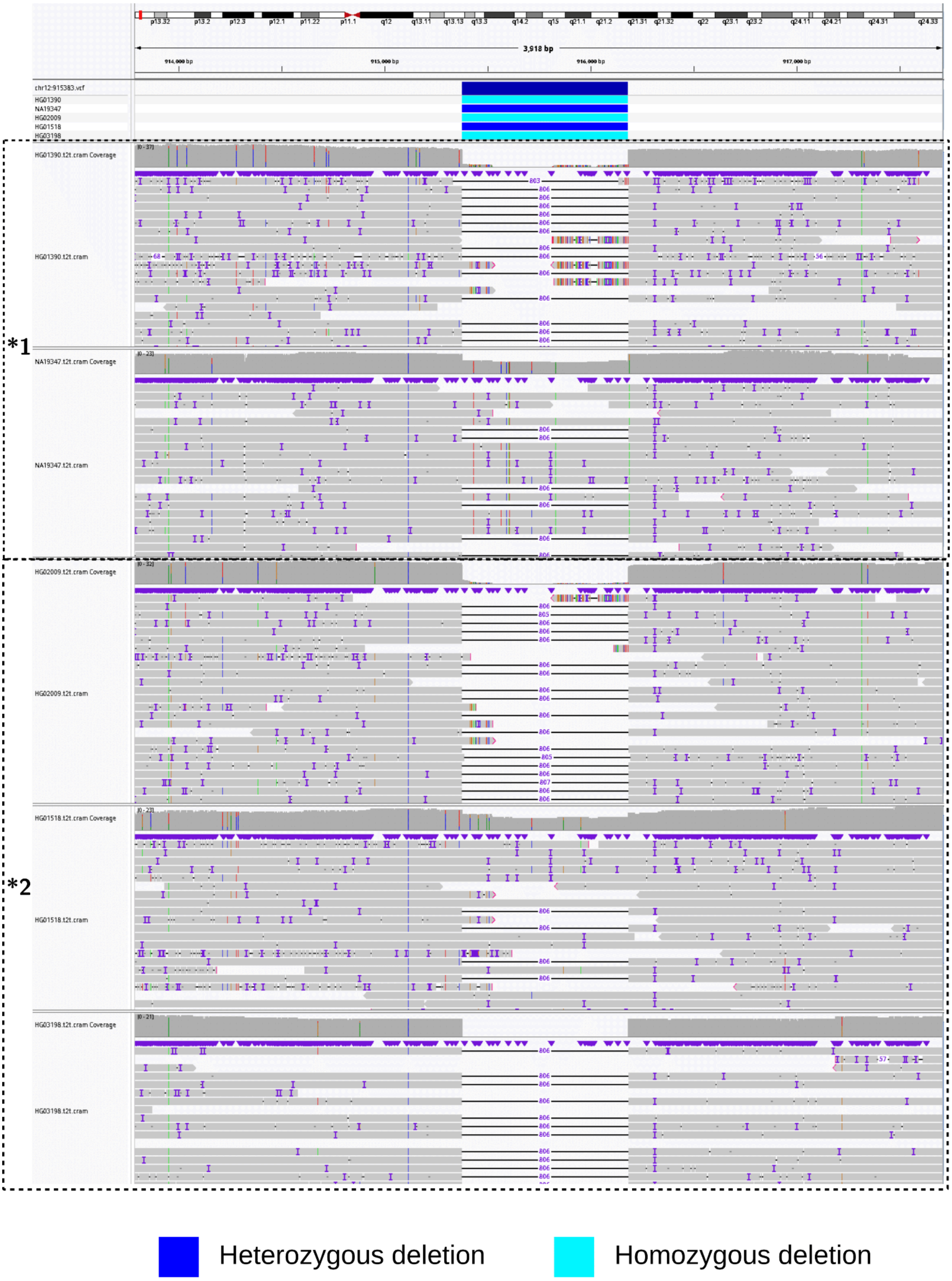

Supplementary Figure 38: TheManual inspection of the deletion genotypes of the samples involved in the two predicted independent occurrences of the deletion event, marked as *1 and *2 in Extended Data Figure 9, these events are validated by manual inspection of the using aligned sequencing reads., shown on the

right side.

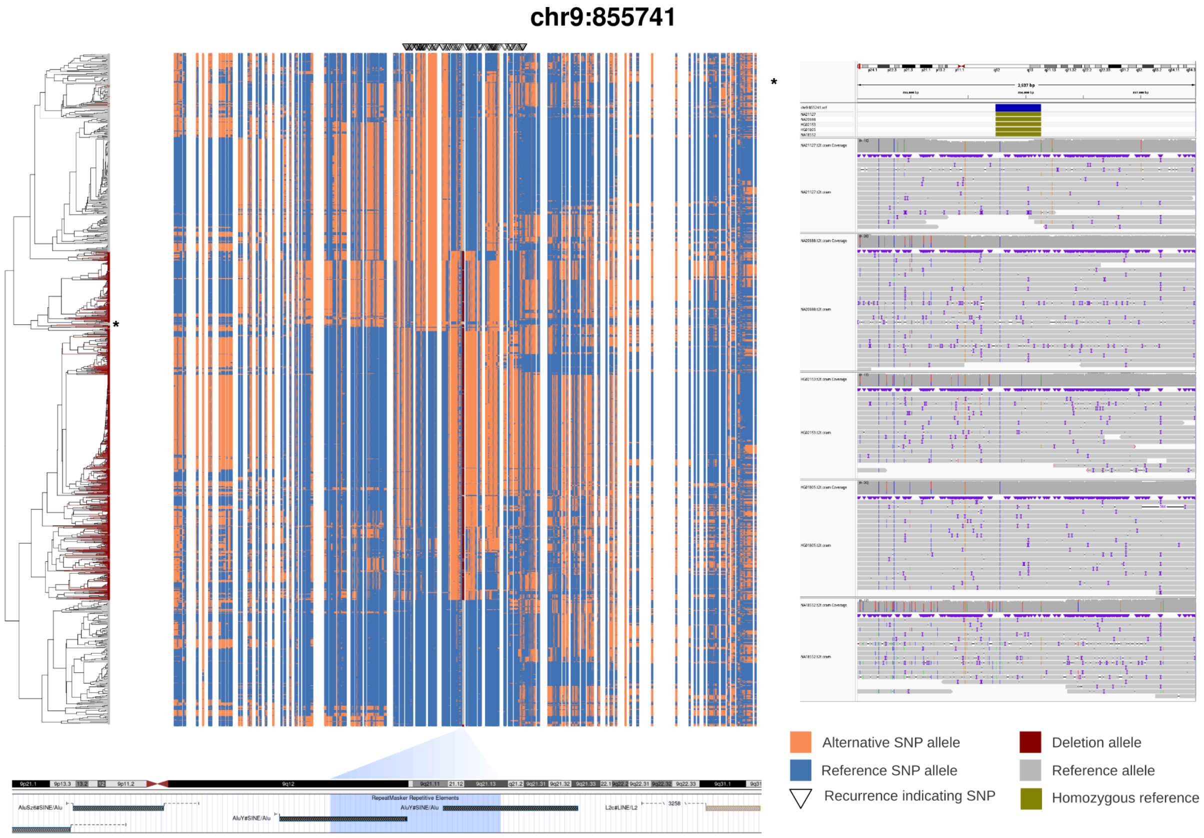

Supplementary Figure 39: A predicted recurrent deletion at 9p24.3 mediated by an AluY-AluY pair. The figure shows the variation of haplotypes in a 100 kb window centered around the deletion and the relationship between haplotypes with (red) and without the deletion (grey). Dendrograms of haplotypes are plotted using a centroid hierarchical clustering method. In each haplotype, reference and alternative alleles are shown in blue and orange, respectively. SNPs within 20 kb around the deletion and showing evidence of deletion recurrence are marked by triangles at the top. A group of haplotypes not carrying the deletion but appearing with the haplotypes carrying it, hence supporting the recurrence of the respective deletion event, are marked by *. The genotypes of these samples were verified by manual inspection of the aligned sequencing reads, shown on the right side.

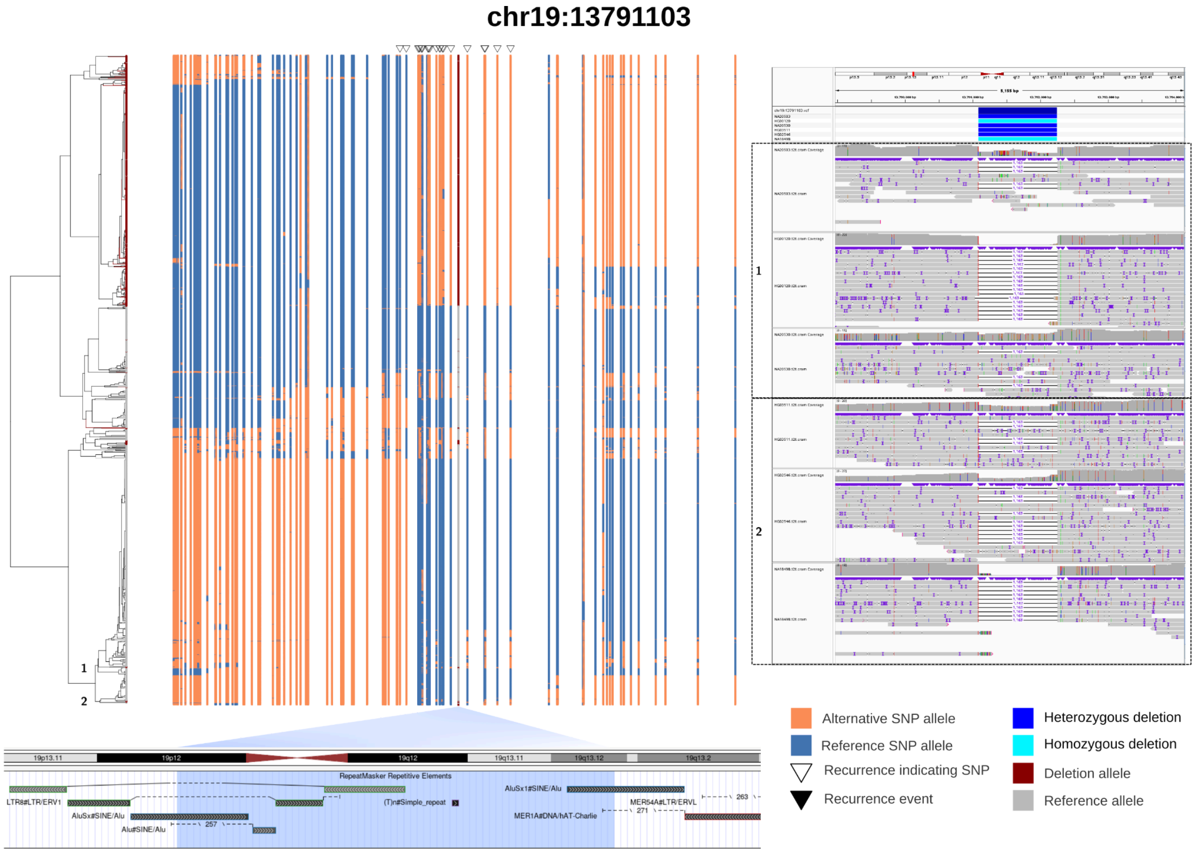

Supplementary Figure 40: A predicted recurrent deletion at 19p13.13 mediated by an AluSx-AluSx pair. The figure shows the variation of haplotypes in a 100 kb window centered around the deletion and the relationship between haplotypes with (red) and without the deletion (grey). Dendrograms of haplotypes are plotted using a centroid hierarchical clustering method. In each haplotype, reference and alternative alleles are shown in blue and orange, respectively. SNPs within 20 kb around the deletion showing evidence of deletion recurrence are marked by triangles at the top. Two predicted independent occurrences of the deletion event are marked as 1 and 2. The deletion genotypes of the samples involved in these events were verified by manual inspection of the aligned sequencing reads, shown on the right side.

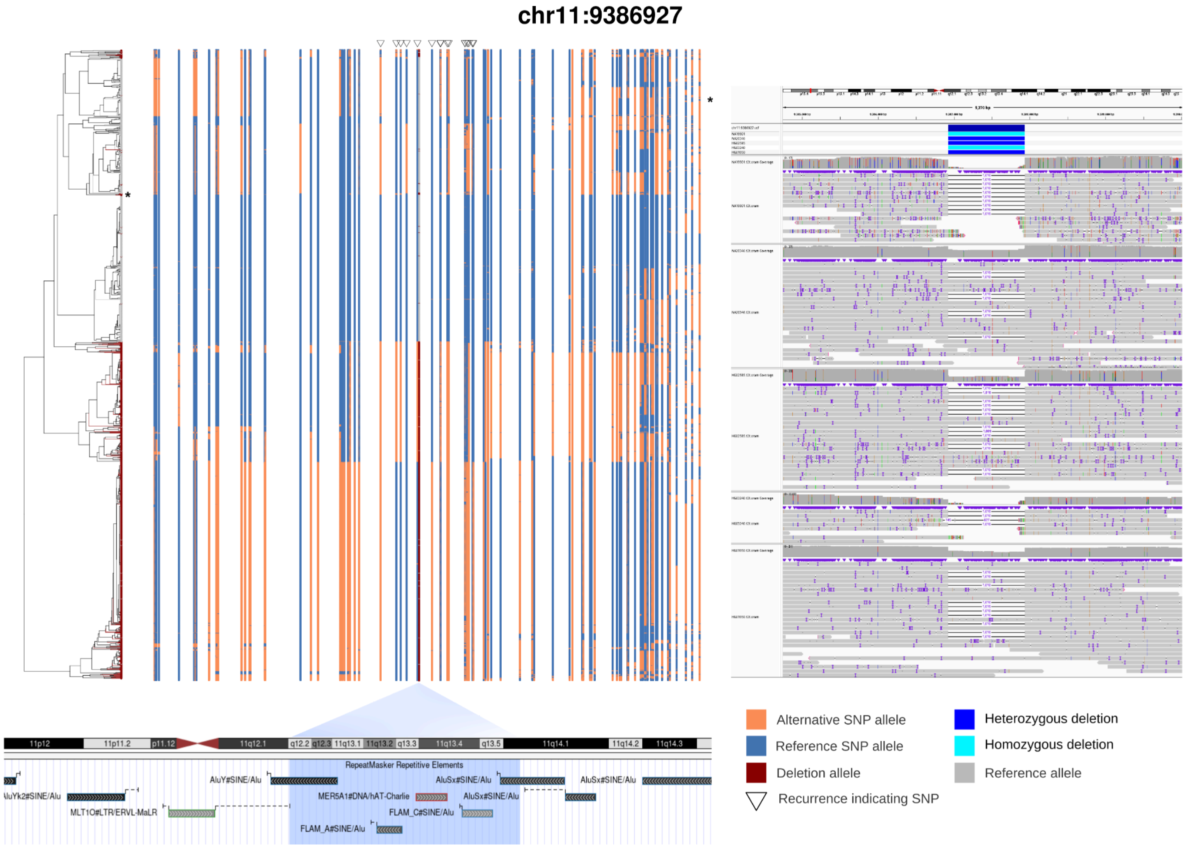

Supplementary Figure 41: A predicted recurrent deletion at 11p15.4 mediated by an AluY-AluSx pair. The figure shows the variation of haplotypes in a 100 kb window centered around the deletion and the relationship between haplotypes with (red) and without the deletion (grey). Dendrograms of haplotypes are plotted using a centroid hierarchical clustering method. In each haplotype, reference and alternative alleles are shown in blue and orange, respectively. SNPs within 20 kb around the deletion showing evidence of deletion recurrence are marked by triangles at the top. A predicted independent occurrence of the deletion event is marked by *. The deletion genotypes of the samples involved in this event were verified by manual inspection of the aligned sequencing reads, shown on the right side.

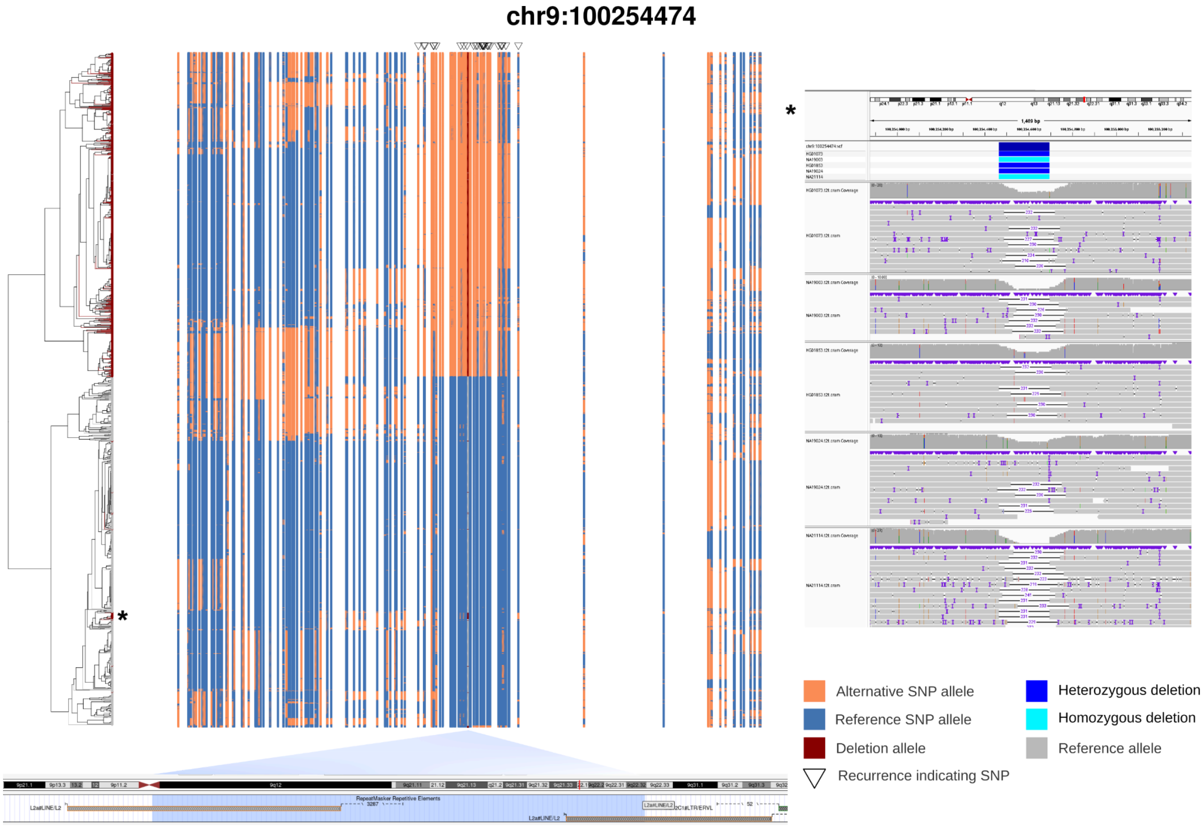

Supplementary Figure 42: A predicted recurrent deletion at 9q22.1 mediated by an L2-L2 pair. Figure shows the variation of haplotypes in a 100 kb window centered around the deletion and the relationship between haplotypes with (red) and without the deletion (grey). Dendrograms of haplotypes are plotted using a centroid hierarchical clustering method. In each haplotype, reference and alternative alleles are shown in blue and orange, respectively. SNPs within 20 kb around the deletion showing evidence of deletion recurrence are marked by triangles at the top. The predicted independent occurrence of the deletion event is marked by *. The deletion genotypes of the samples involved in this event were verified by manual inspection of the aligned sequencing reads, shown on the right side.

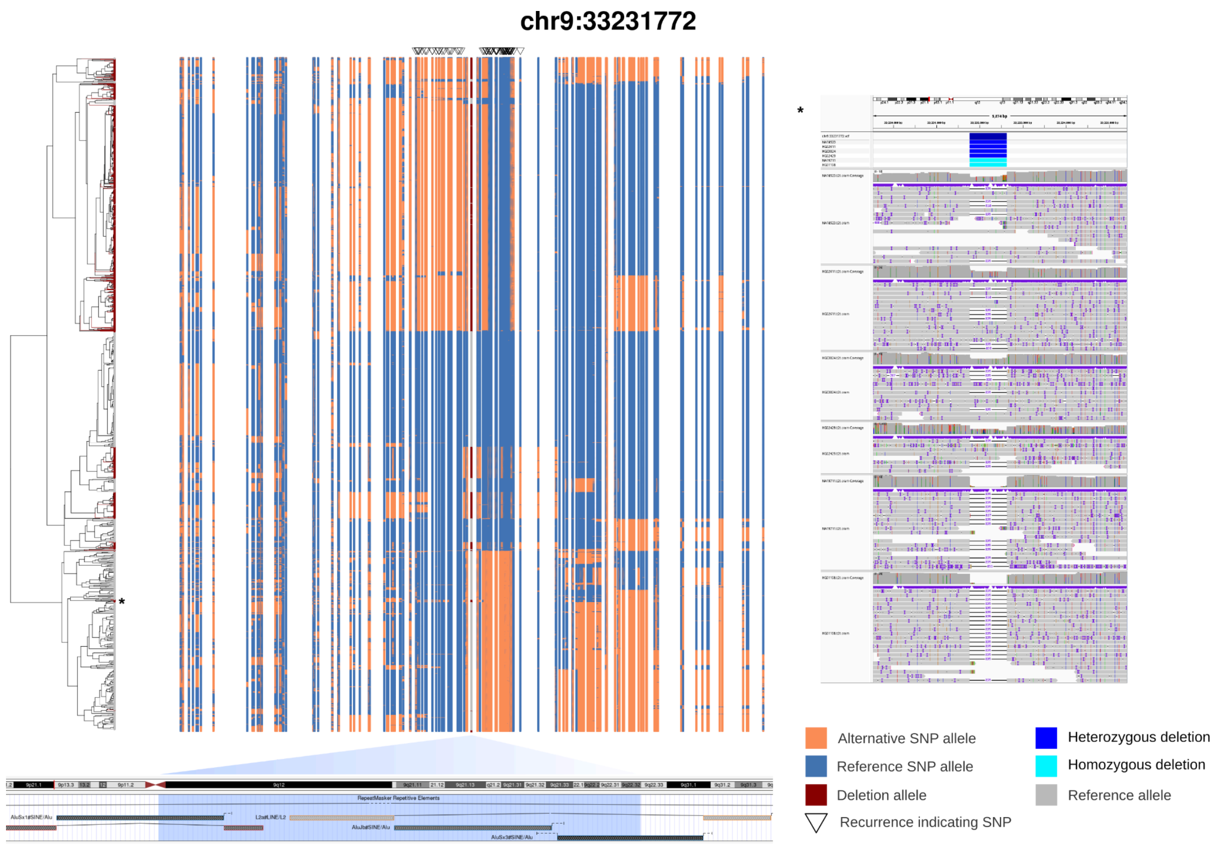

Supplementary Figure 43: A predicted recurrent deletion at 9p13.3 mediated by an AluSx-AluSx pair. The figure shows the variation of haplotypes in a 100 kb window centered around the deletion and the relationship between haplotypes with (red) and without the deletion (grey). Dendrograms of haplotypes are plotted using a centroid hierarchical clustering method. In each haplotype, reference and alternative alleles are shown in blue and orange, respectively. SNPs within 20 kb around the deletion showing evidence of deletion recurrence are marked by triangles at the top. A predicted independent occurrence of the deletion event is marked by *. The deletion genotypes of the samples involved in this event were verified by manual inspection of the aligned sequencing reads, shown on the right side.

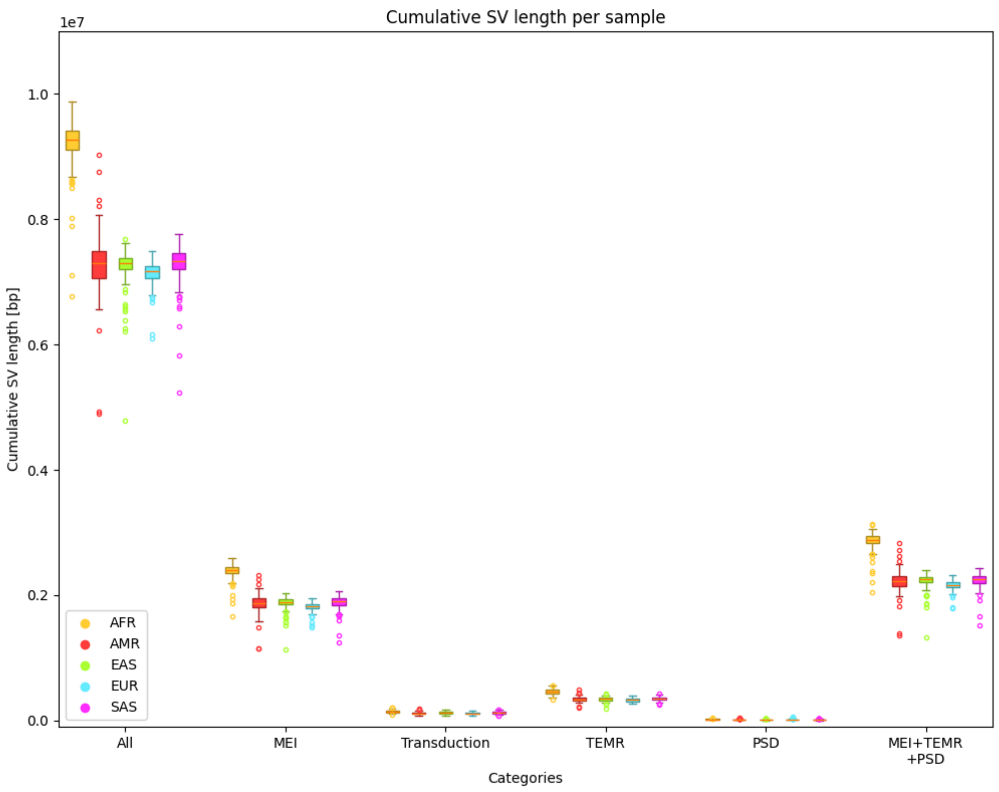

Supplementary Figure 44: Cumulative SV length per sample for subsets of mobile-element associated SVs stratified by superpopulation. From the callset, different SV subsets were extracted and the cumulative SV length per sample was determined by summing the length of each SV present in the sample. For the calculation of cumulative transduction size not the SV length but the transduction length determined by SVAN was summed. The results were stratified by superpopulation. ME: mobile elements, TD: transduction, PSD: pseudogene, TEMR: transposable element-mediated rearrangement.

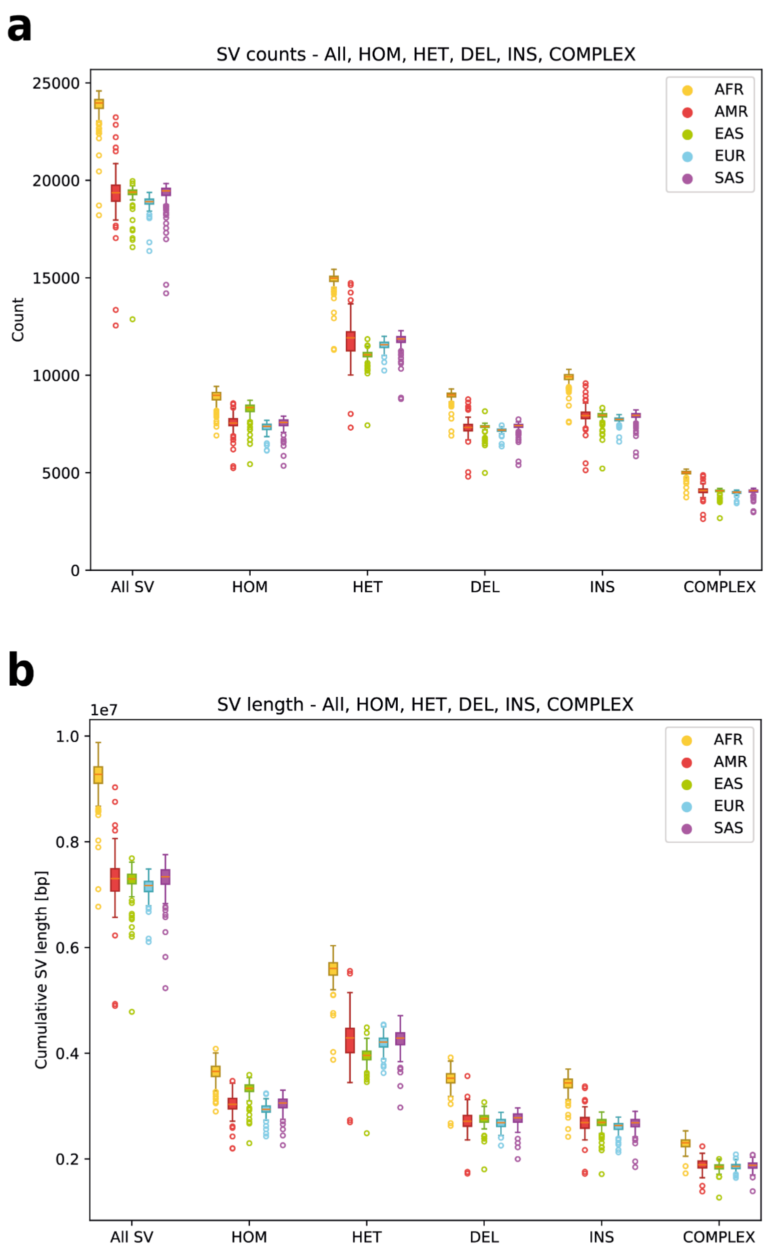

Supplementary Figure 45: SV count **(a)** and cumulative length **(b)** per sample for all SVs and SV subsets. From the callset, different SV subsets were extracted and the number of SV as well as the cumulative SV length per sample was determined. HET: heterozygous SV, HOM: homozygous SV, DEL: deletion, INS: insertion.

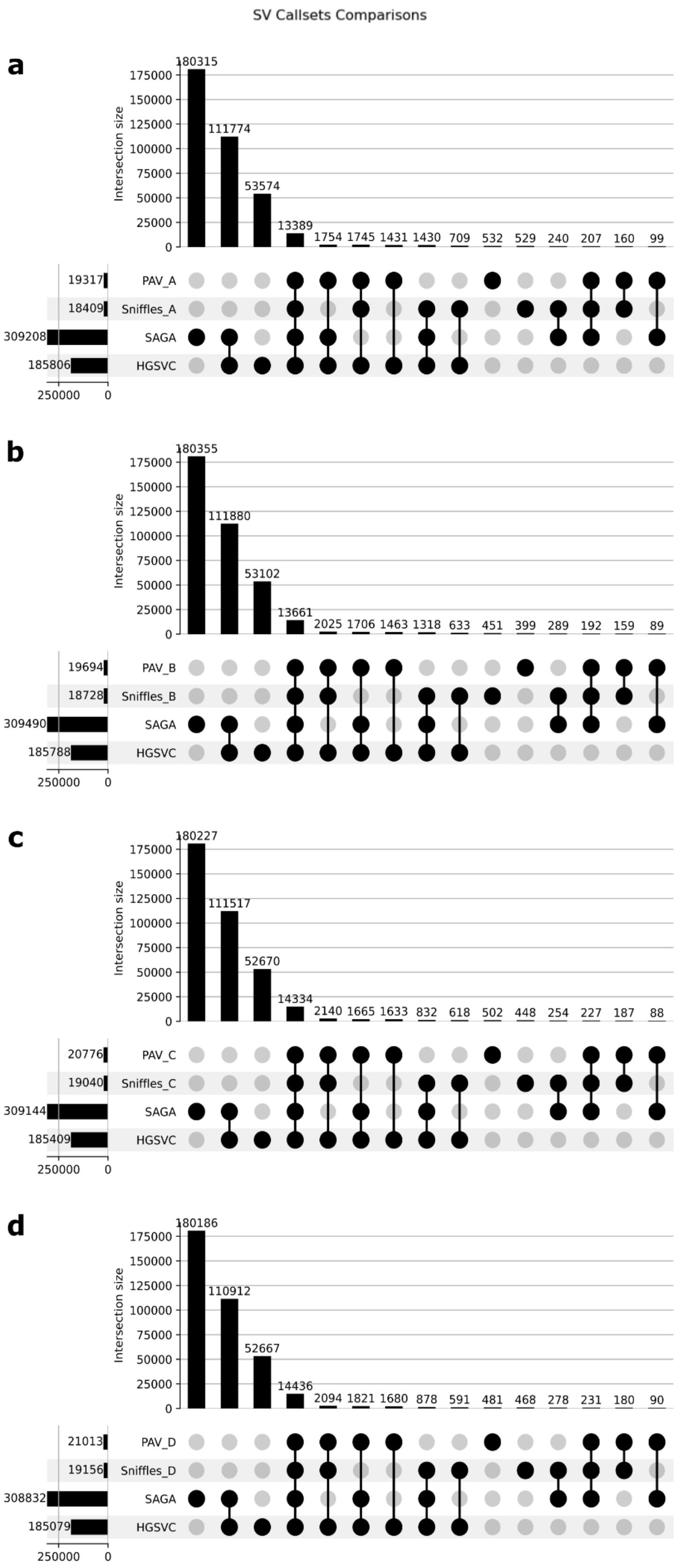

Supplementary Figure 46: Comparison between SV callsets from four rare disease patients (Patient A-D in panel a-d, respectively) generated by PAV and Sniffles, the phased VCF panel of HPRC_mg_44+966 given as input for genotyping and the Logsdon *et al.* (HGSVC3) SV callset[14] generated from multi-platform whole genome high quality assemblies. After filtering intersection, we found **a)** 160, **b)** 159, **c)** 187 and **d)** 180 SVs exclusive to each rare disease patient genome, respectively.

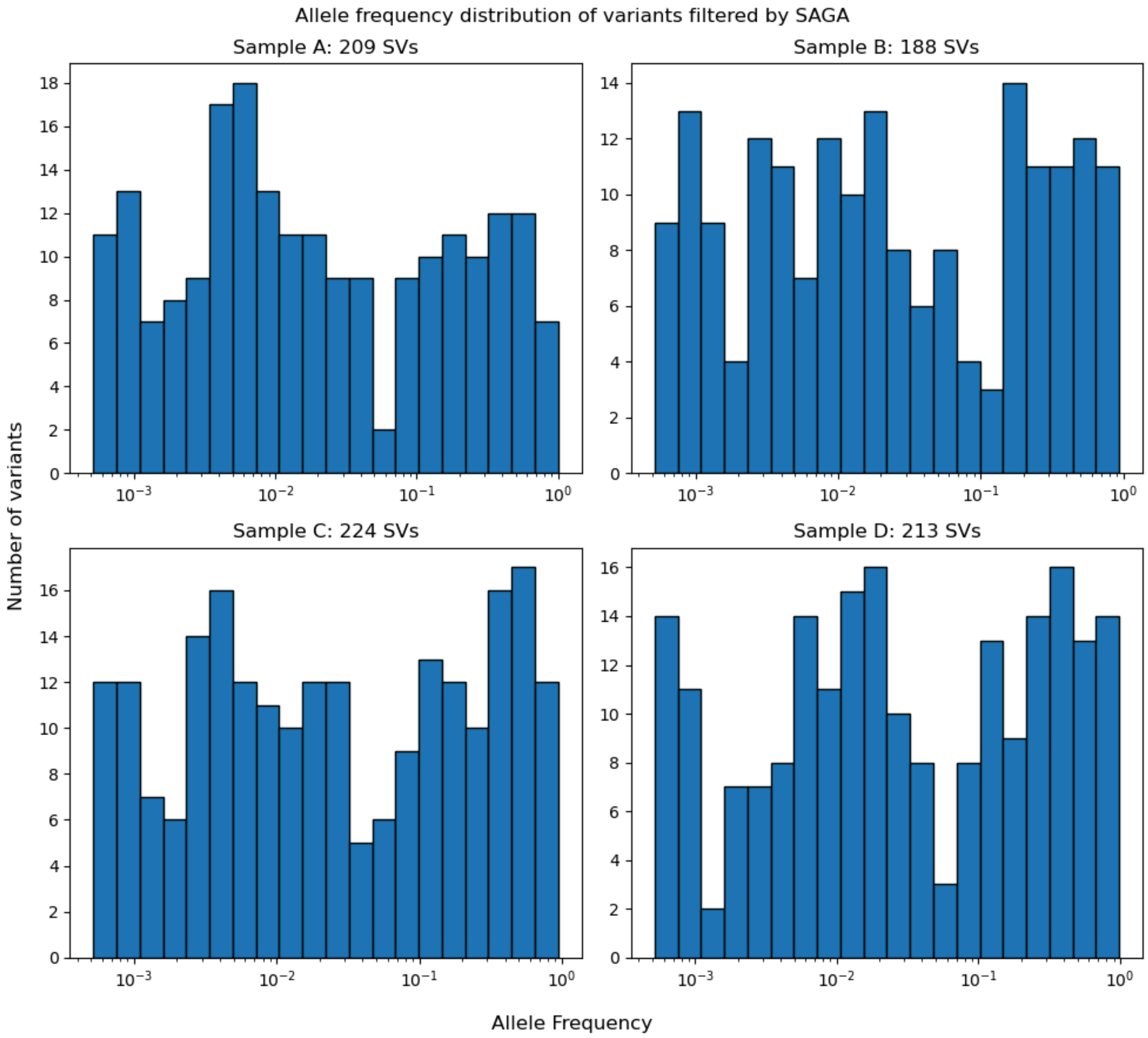

Supplementary Figure 47: Allele frequency distributions (log-scale) shown for SV alleles from our study matching those from rare disease patients A-D. These variants were successfully filtered using our SAGA-based SV resource but were missed in a recent study involving multi-platform whole-genome assembly[14]. Consistent with current multi-platform whole genome assembly efforts targeting smaller sample sizes[14], as shown here, these SV alleles are mostly rare in the population. SAGA's VCF filters out mostly rare SVs.

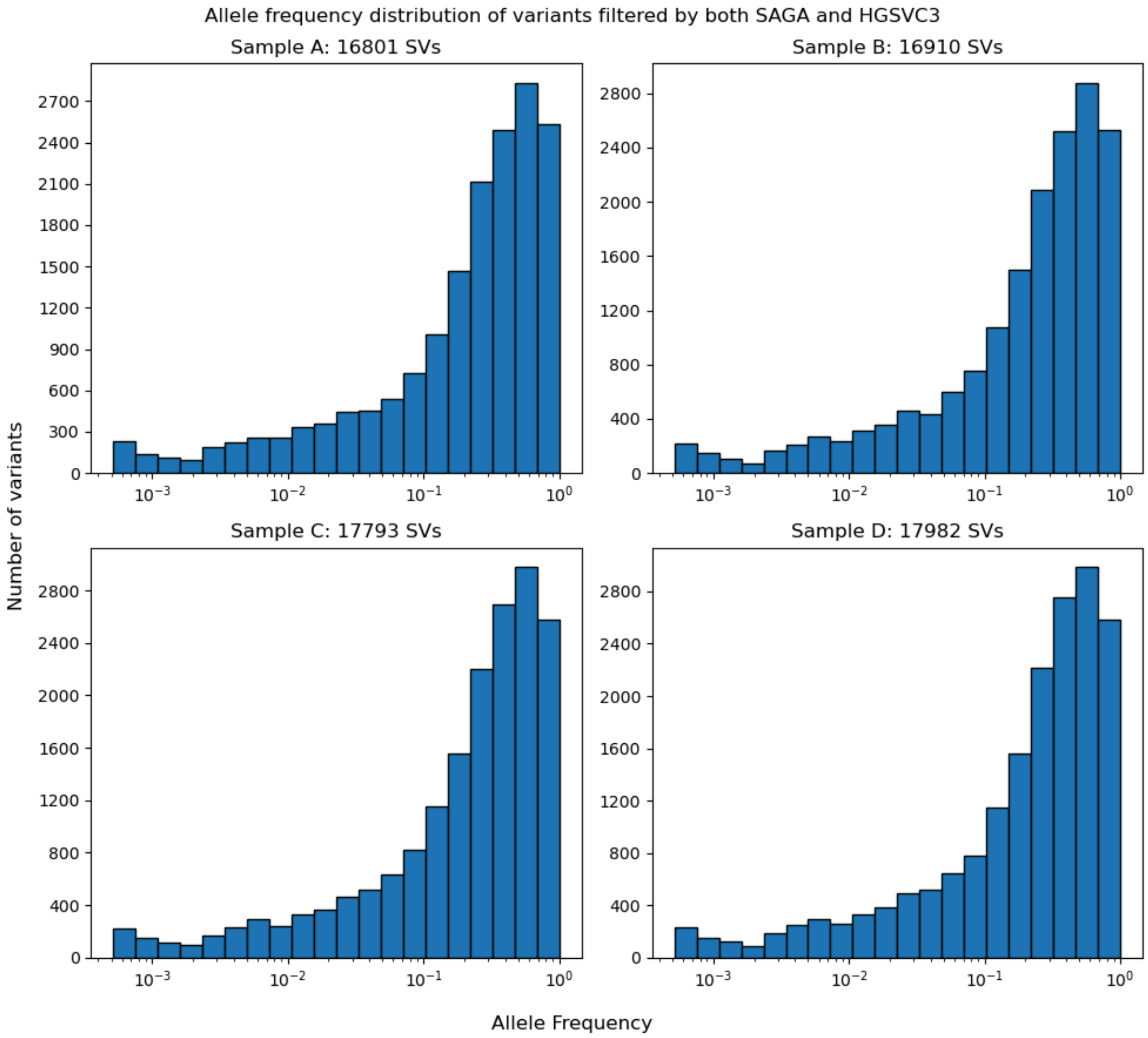

Supplementary Figure 48: Allele frequency distributions (log-scale) shown for SV alleles from our study matching those from rare disease patients A-D, here shown for SVs filtered out both by SAGA and the HGSVC multi-platform assembly based dataset[14]. The majority of these sites represent common SVs.

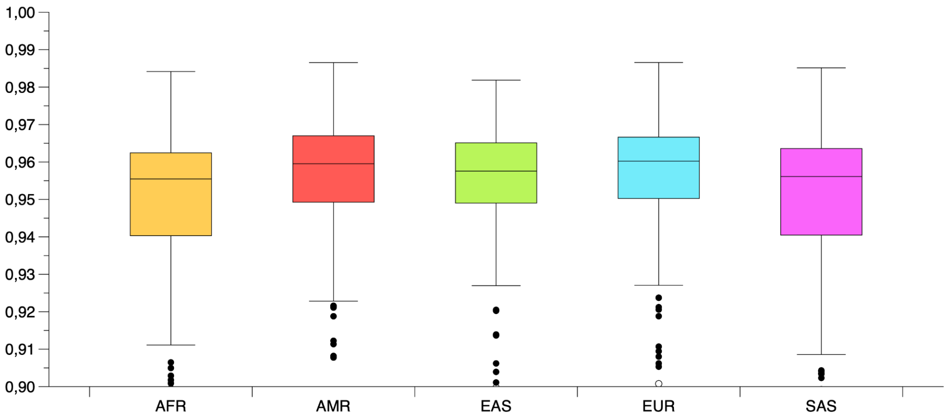

Supplementary Figure 49: Fraction of CHM13 bases covered at least five-fold. Y-axis truncated at 0.9.

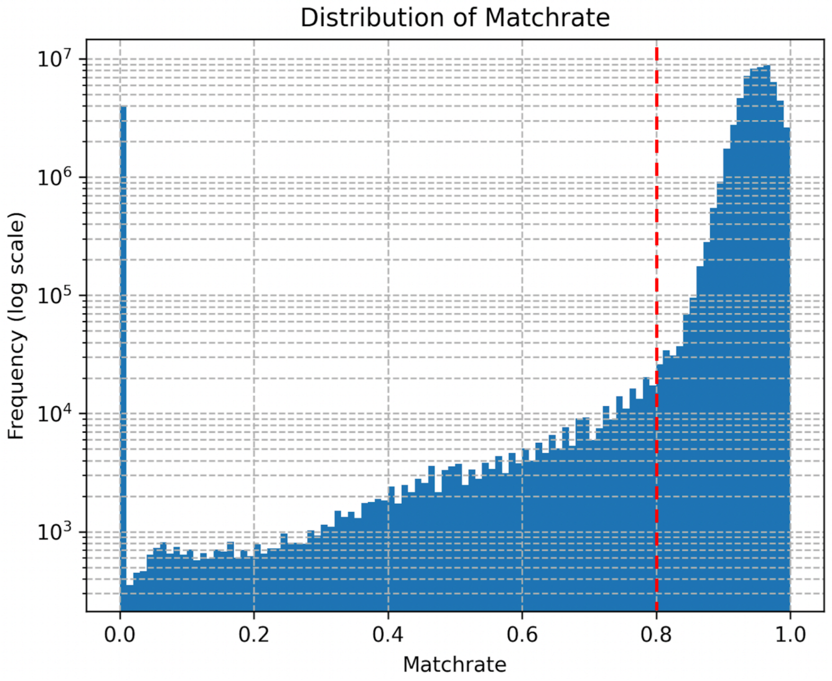

Supplementary Figure 50: Distribution of Matchrate Indicating Regions Selected for Realignment. The histogram displays the frequency distribution of match rates across genomic regions in NA12878, plotted on a logarithmic scale. A match rate threshold of 0.8 (delineated by the dashed red line) was established based on the observed distribution, where regions exhibiting a match rate below this value were identified as candidates for realignment. These regions, representing a significantly lower match rate compared to the majority, were subsequently realigned using NGMLR to enhance the accuracy of inversion detection. This thresholding approach ensures the prioritization of genomic regions most likely to benefit from the increased sensitivity and specificity of NGMLR in identifying small inversions.

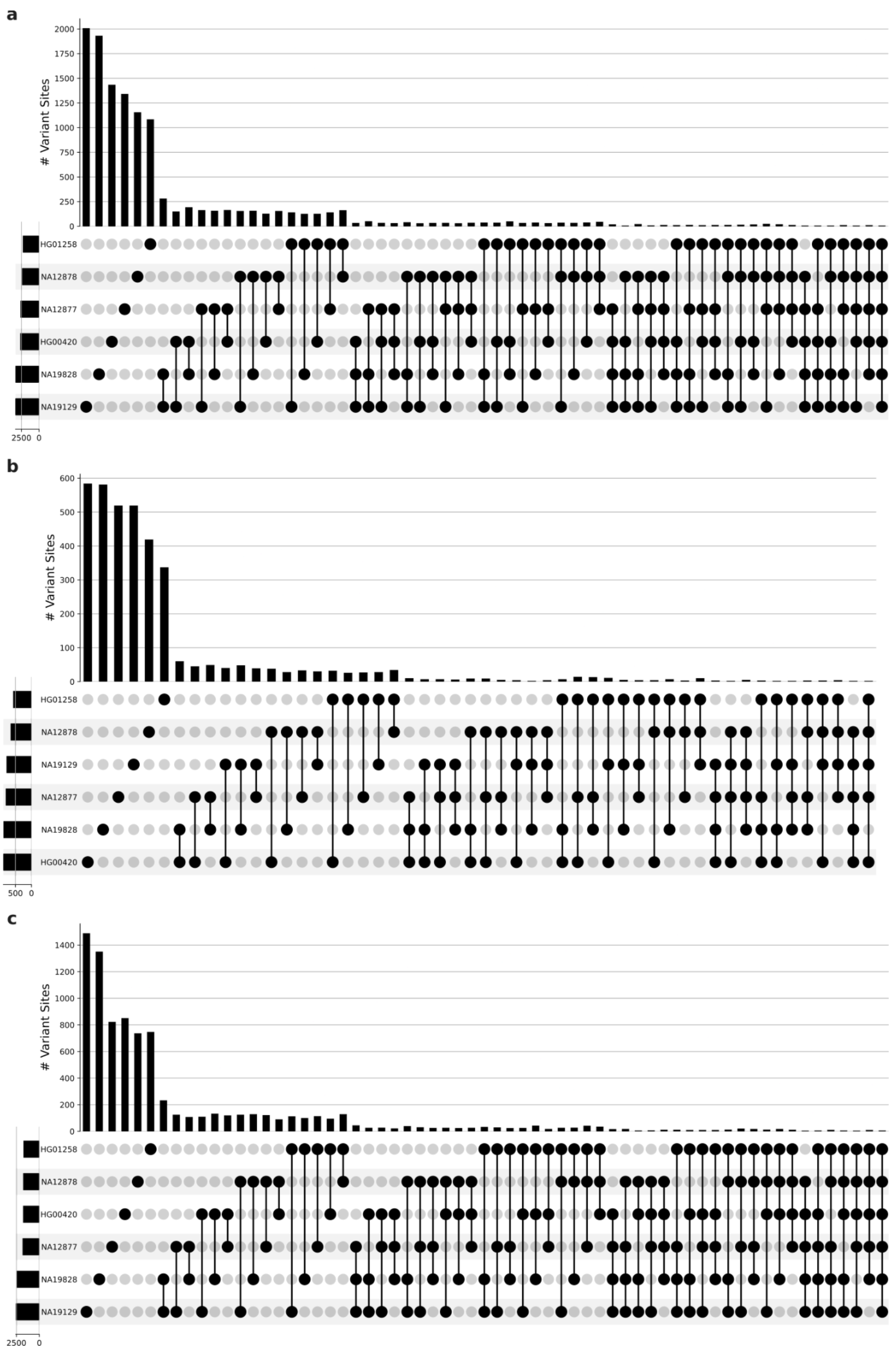

Supplementary Figure 51: The figure shows the intersection of the variants which failed Mendelian consistency as an upset plot. The genotypes for which the upset plot has been made come from Giggles genotyping on the HPRC_mg_44+966 graph which have been further filtered to give the strict set. **a)** shows the intersection of all variants, **b)** for variants from biallelic bubbles and **c)** for variants from multiallelic bubbles. The six families are represented by the child sample.

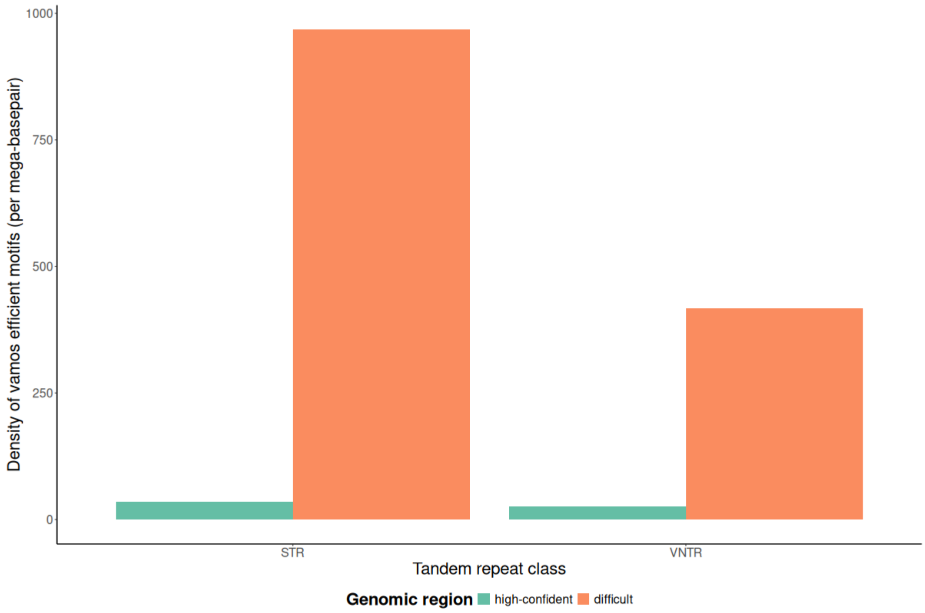

Supplementary Figure 52: VNTR density by GiaB difficult and high-confidence regions. Using the vamos efficient motif set for short (STR) and variable number of tandem repeat (VNTR) annotation, we computed the autosomal density of these motifs for high-confident and difficult genomic regions (based on GiaB genomic masks).

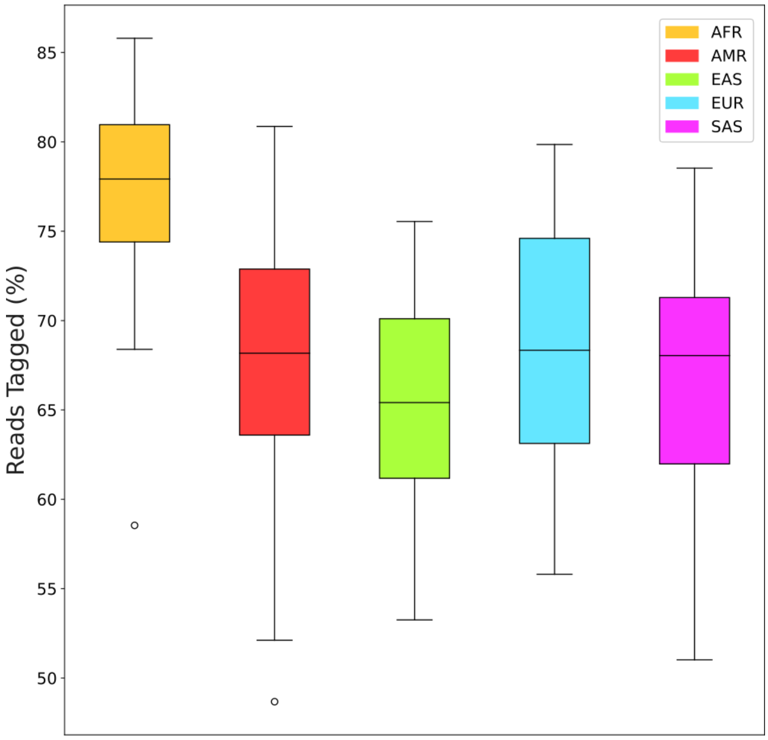

Supplementary Figure 53: The percentage of reads tagged by WhatsHap[1] haplotag command. The ONT reads for each sample are haplotype-tagged using the New York Genome Center (NYGC) statistical phased VCF[2].

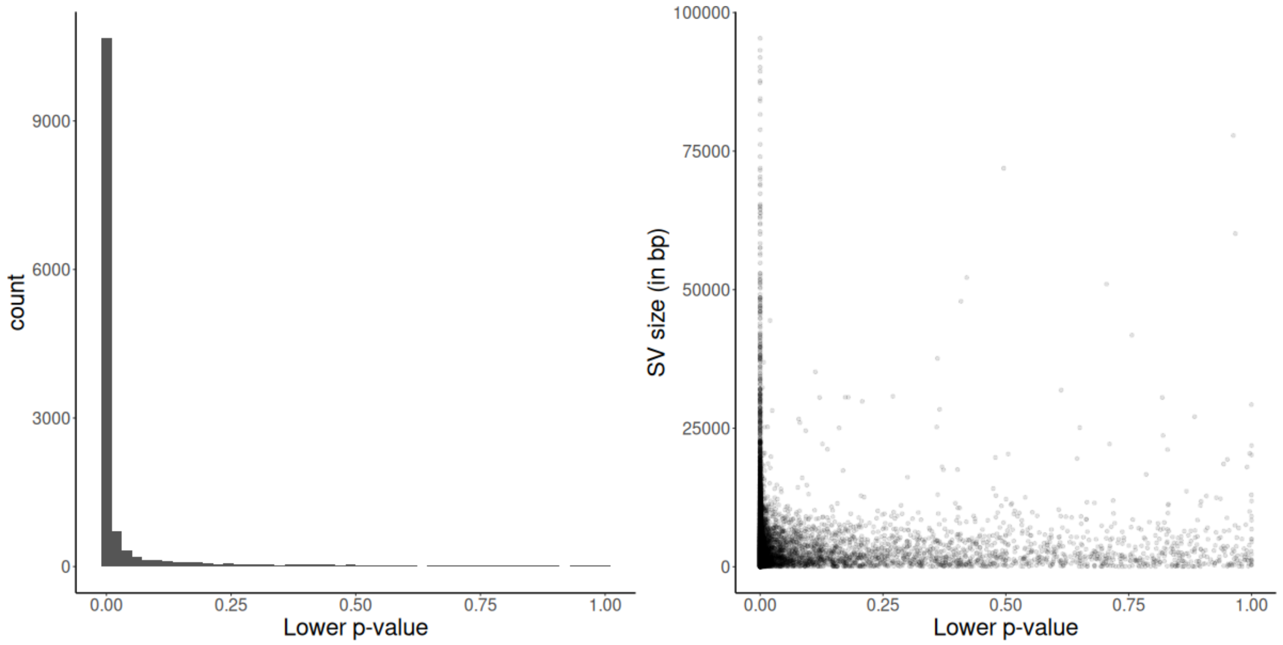

Supplementary Figure 54: Intensity rank sum (IRS) test[54] using SNP array probe intensity data. Left panel shows the p-value distribution for all deletions that can be assessed via IRS (13,788 deletions) and the right panel shows a scatter plot of p-value and SV size. Overall estimated FDR for deletions based on IRS is 8.06%.

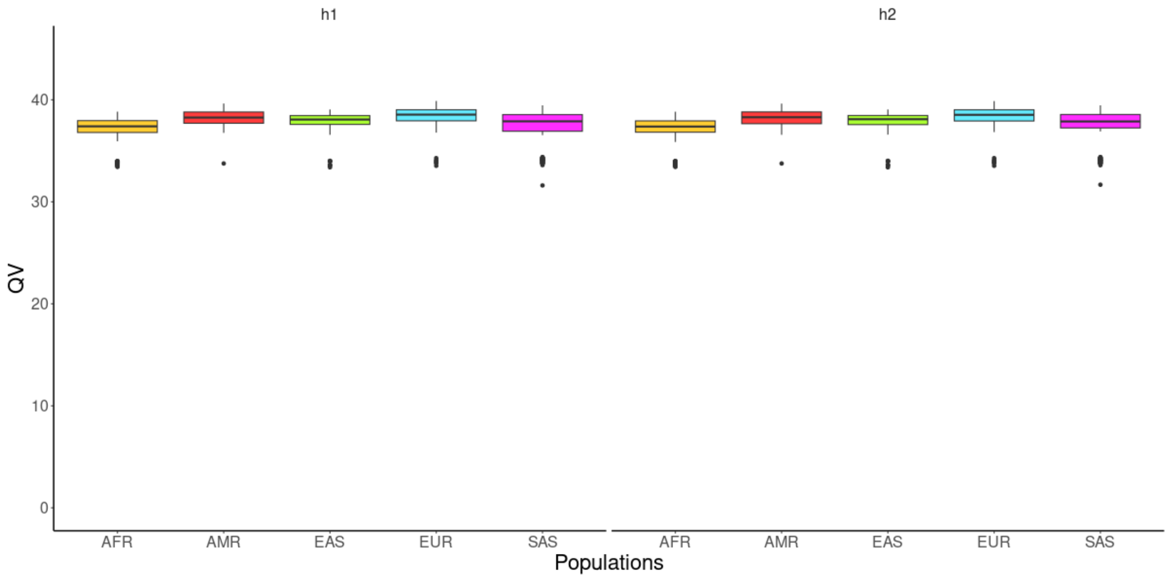

Supplementary Figure 55: Evaluation of variant completeness and phasing accuracy by integrating all variants (SNPs, InDels and SVs) into sample-specific haplotypes (h1 and h2) based on the CHM13 genome. Haplotype completeness and accuracy was evaluated using QV scores computed by yak[37] with k-mers of length 31 compared to the high-depth, short-read sequencing data. QV scores are lower compared to recently published high-quality HPRC assemblies[50] but in the range of high-depth *de novo* long-read assemblies[55].

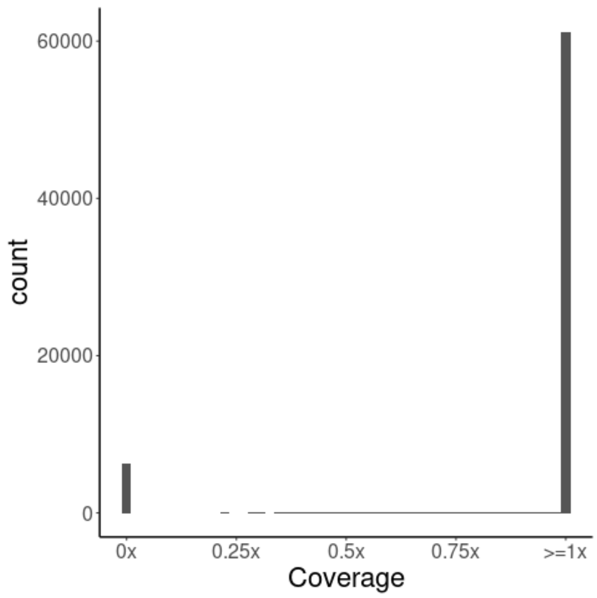

Supplementary Figure 56: Coverage analysis of the insertion sequences using the compacted de Bruijn graphs computed from prior short-read data[2]. For a singleton SV occurring in only one sample (allele count one) the expected coverage is 1x.

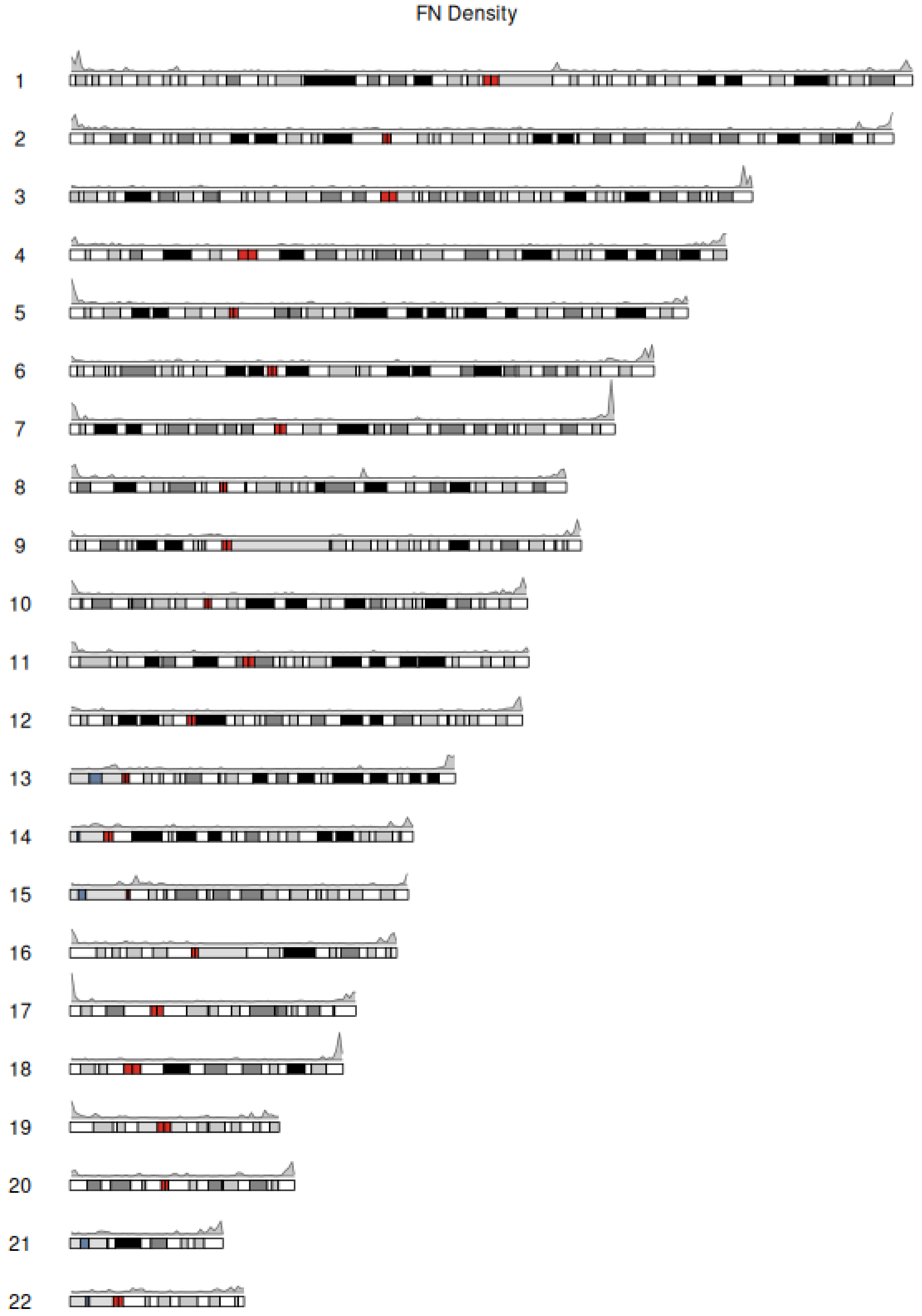

Supplementary Figure 57: Evaluation of the false negative (FN) density along autosomal chromosomes reveals increased numbers of false negatives near telomeres on the CHM13 (T2T) reference genome because the graph augmentation implemented in SAGA uses pseudo-haplotypes built using the GRCh38 reference genome where telomeres are often unresolved.

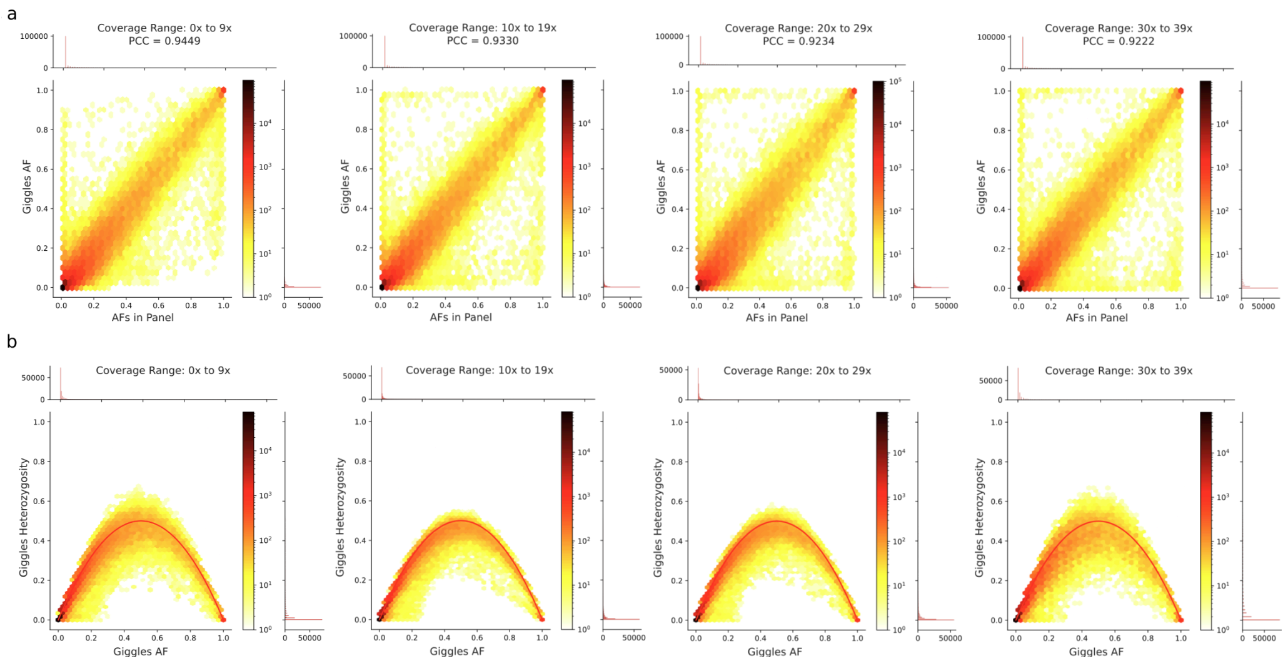

Supplementary Figure 58: Genotyping quality assessment when stratifying samples by coverage. The plot demonstrates the genotype quality of the genotypes by Giggles on the HPRC_mg_44+966 graph after filtering across four sequencing coverage ranges (<10×; ≥10× and <20×; ≥20× and <30×; ≥30x and <40×). A) shows genotyping quality using a comparison of the SV allele frequency of an allele in the VCF panel (created using the HPRC assemblies and the pseudo-haplotypes of the SAGA framework) with the allele frequency of the same allele genotyped by Giggles in the callset (using only the 908 unrelated samples from our callset). B) Genotyping quality using a Hardy-Weinberg Equilibrium (HWE) plot, with the SV allele frequency (AF) of the genotyped allele plotted versus the percentage of samples heterozygous for that allele (using only the 908 unrelated samples from our callset).

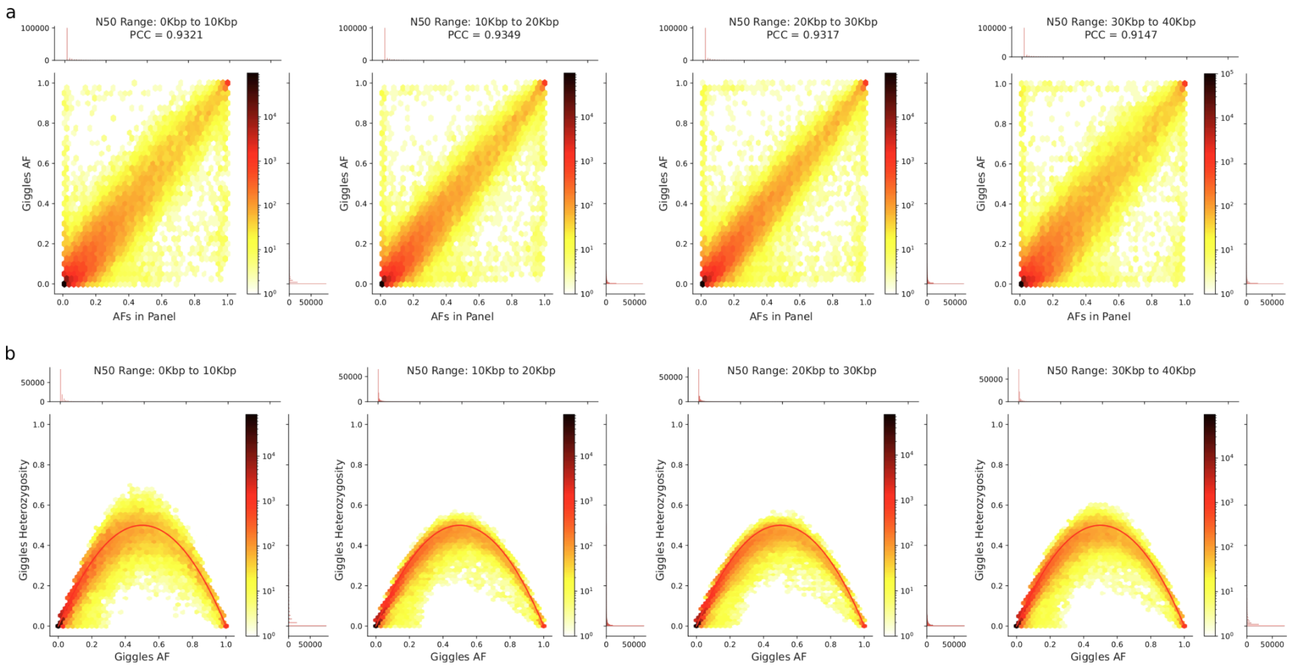

Supplementary Figure 59: Genotyping quality assessment when stratifying samples by N50. The plots demonstrate the genotype quality of the genotypes by Giggles on the HPRC_mg_44+966 graph after filtering across four sample N50 ranges (<10 kb; ≥10 kb and <20 kb; ≥20 kb and <30 kb; ≥30 kb and <40 kb). A) shows genotyping quality using a comparison of the SV allele frequency of an allele in the VCF panel (created using the HPRC assemblies and the pseudo-haplotypes of the SAGA framework) with the allele frequency of the same allele genotyped by Giggles in the callset (using only the 908 unrelated samples from our callset). B) Genotyping quality using a Hardy-Weinberg Equilibrium (HWE) plot, with the SV allele frequency (AF) of the genotyped allele plotted versus the percentage of samples heterozygous for that allele (using only the 908 unrelated samples from our callset).

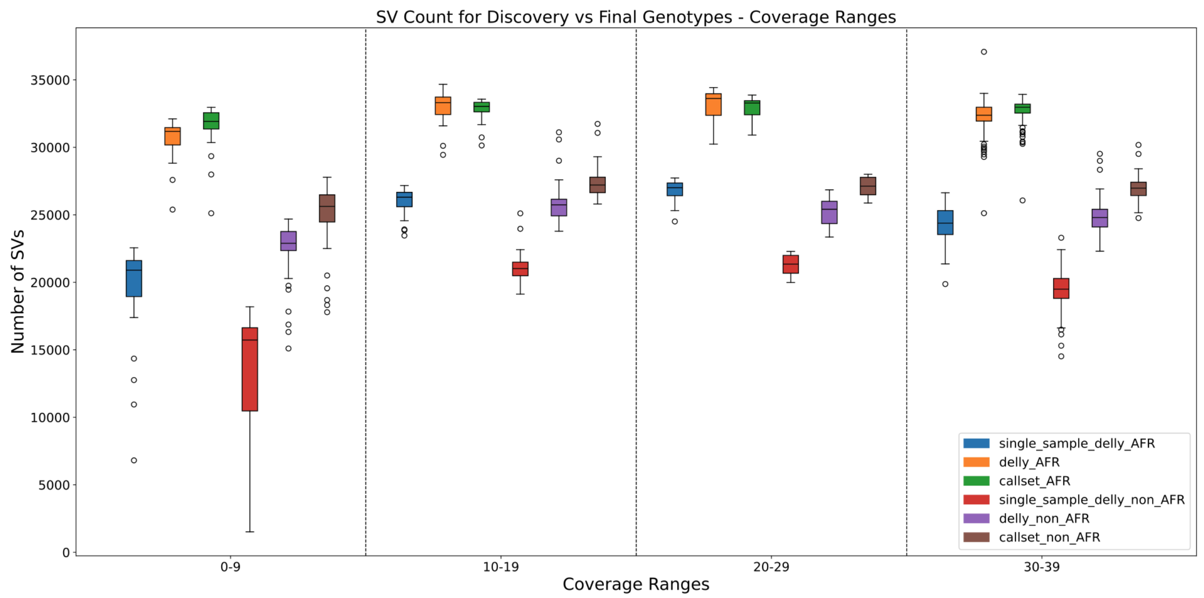

Supplementary Figure 60: Assessment of the effect of coverage on the number of SV sites per sample after integration with the SAGA framework. Differences in SV discovery between single-sample SV calling with DELLY (labelled 'single_sample_delly'), DELLY population-level SV calling (labelled 'delly')), and SV integration with SAGA followed by Giggles based genotyping ('callset') after stratifying samples into distinctive coverage bins (<10×; ≥10× and <20×; ≥20× and <30×; ≥30x and <40×), as well as into samples of African (AFR) and non-African (non_AFR) ancestry. Note that SV numbers plotted on the y-axis correspond to the number of heterozygous (het) SV sites in the dataset plus twice the number of homozygous (hom) SV sites.

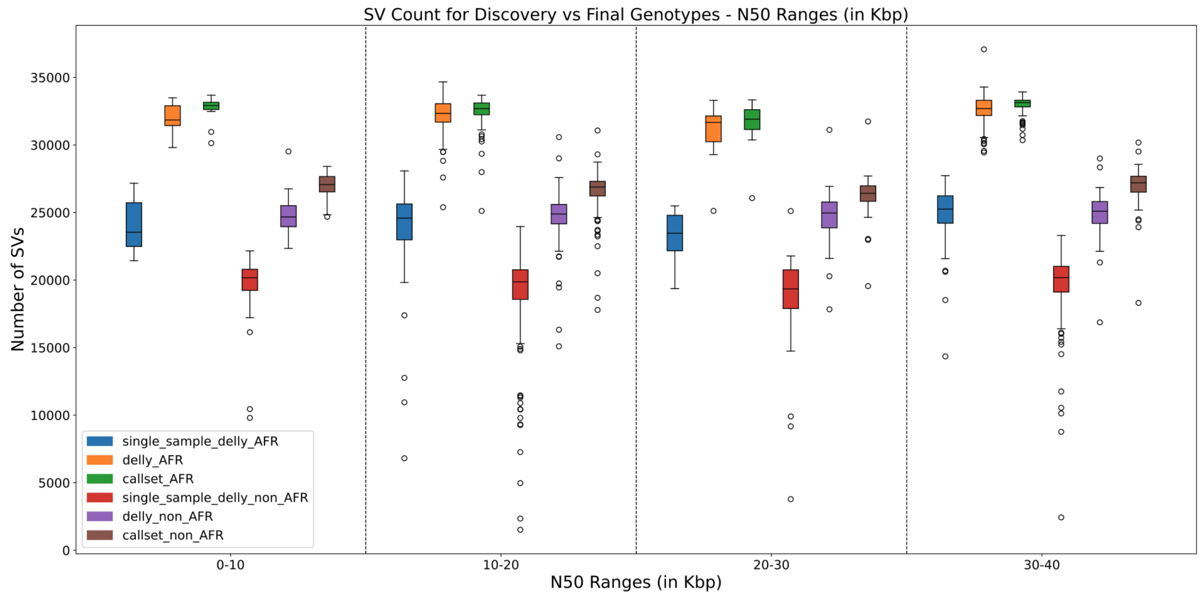

Supplementary Figure 61: Assessment of the effect of read N50 on the number of SV sites per sample after integration with the SAGA framework. Differences in SV discovery between single-sample SV calling with DELLY (labelled 'single_sample_delly'), DELLY population-level SV calling (labelled 'delly')), and SV integration with SAGA followed by Giggles based genotyping ('callset') after stratifying samples into distinctfive read N50 bins (<10 kb; ≥10 kb and <20 kb; ≥20 kb and <30 kb; ≥30 kb and <40 kb), and into samples of African (AFR) and non-African (non_AFR) ancestry. Note that SV numbers plotted on the y-axis

correspond to the number of heterozygous (het) SV sites in the dataset plus twice the number of homozygous (hom) SV sites.

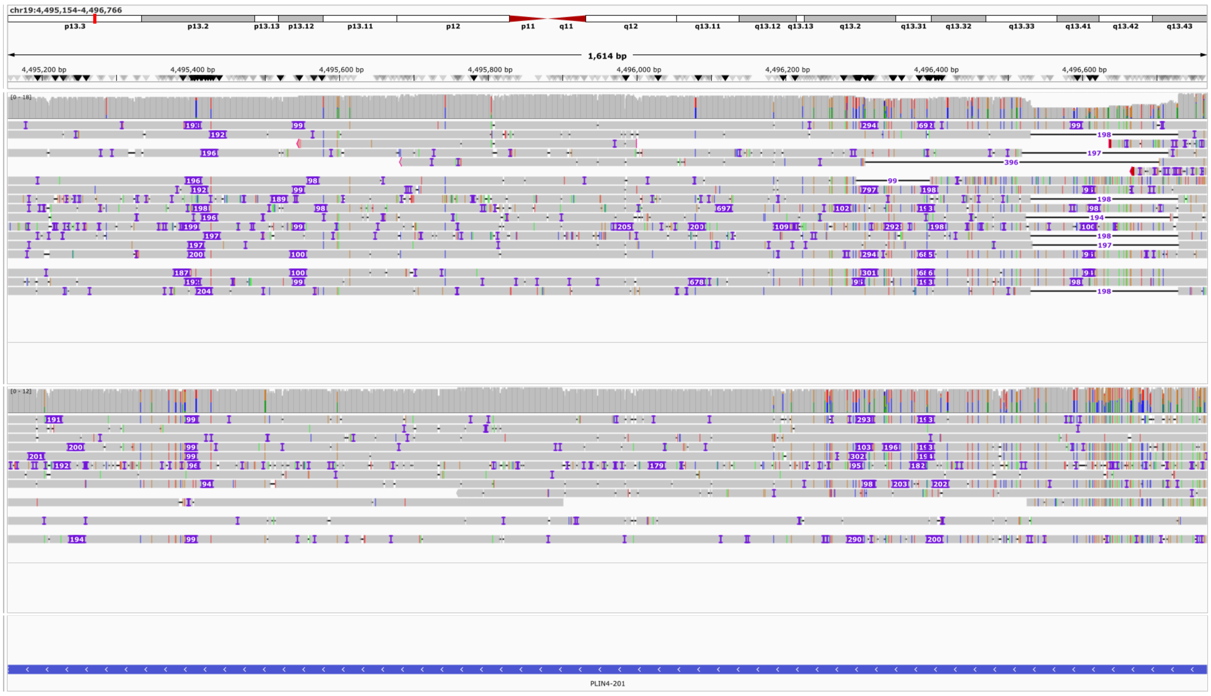

Supplementary Figure 62: IGV plot of the core region of the PLIN4 VNTR locus in NA20127 (upper panel) and HG03508 (lower panel). NA20127 was estimated to carry 43 repeat units and HG03508 37 repeat units using vamos. In support of the vamos based VNTR analysis, read alignments of NA20127 show for one haplotype newly inserted sequences of 678-697 bp in length, indicative of approximately 7 additional repeat units.

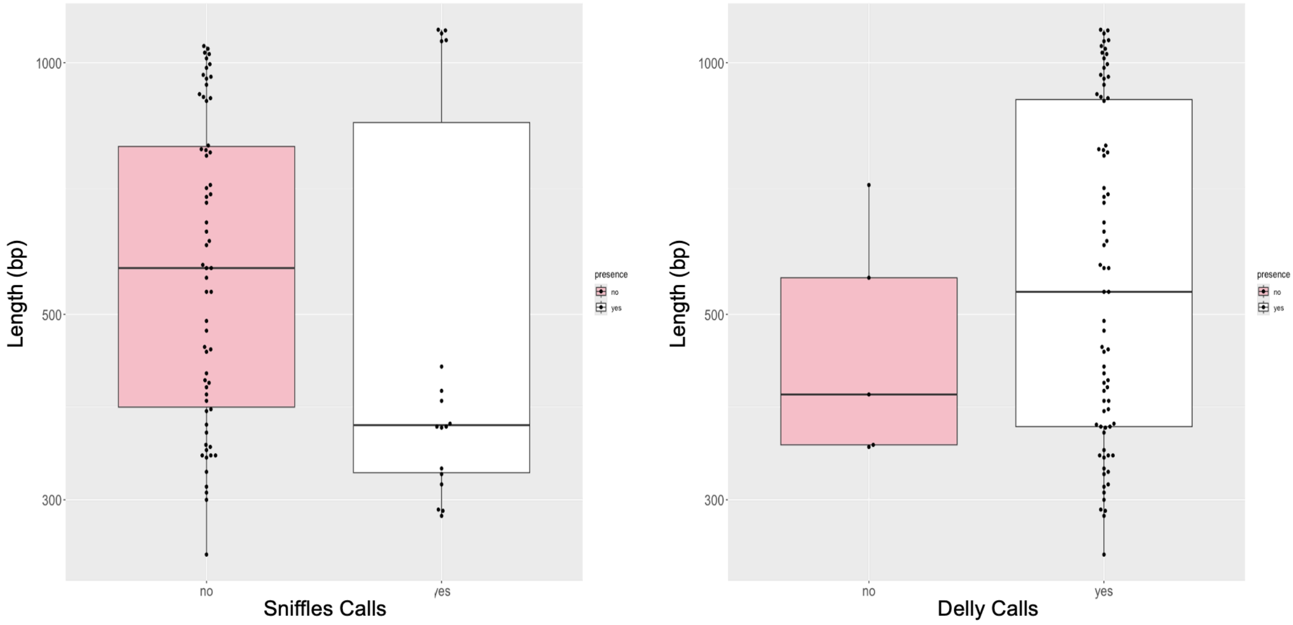

Supplementary Figure 63: Performance of DELLY and Sniffles in the detection of genomic inversions in simulated data. Boxplot comparison of the inversion detection accuracy of DELLY and Sniffles in simulated

data for inversions spanning from 250 bp to 1.1 kb in size. The y-axis indicates the inversion length. The color coding represents the presence (white) or absence (pink) of accurate inversion detection, based on simulated benchmarks. As illustrated in the graph, DELLY consistently identifies accurately inversions greater than 200 bp, whereas Sniffles only performs accurate inversion detection for inversions that exceed 1.1 kb.

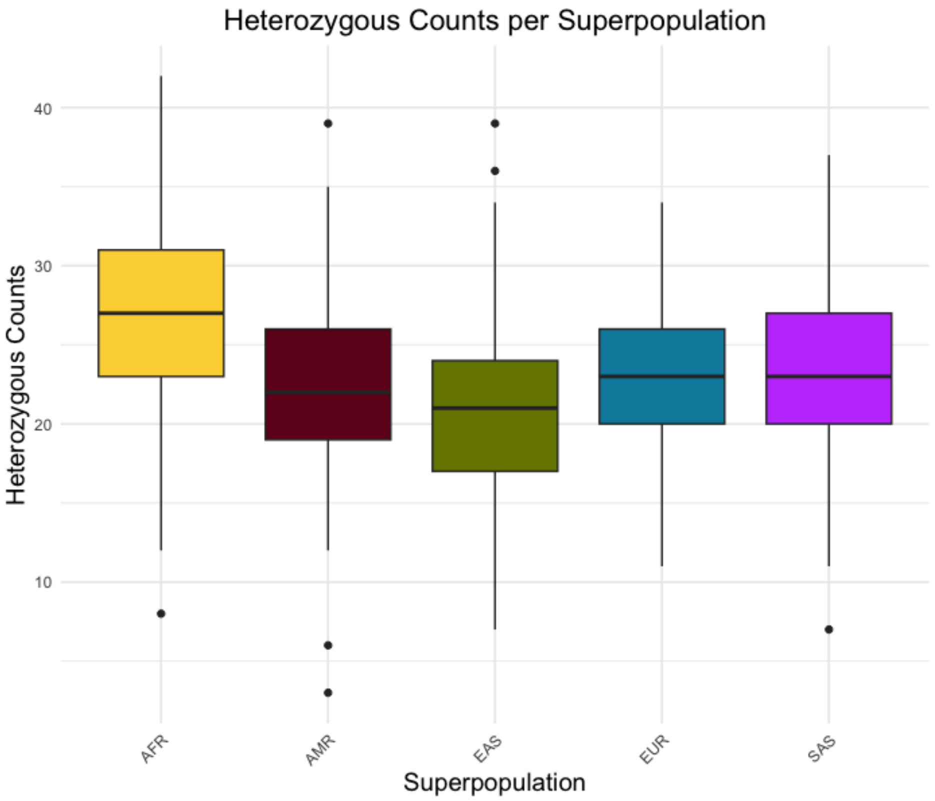

Supplementary Figure 64: The plot displays the distribution of heterozygous inversion counts for each sample, after inversion genotyping, categorized by superpopulations. Notably, the relative increase in heterozygous inversions in AFR samples compared to other ancestries is more pronounced, consistent with the higher heterozygosity typically observed in AFR populations. Conversely, EAS samples exhibit reduced heterozygosity compared to other superpopulations, aligning with the expected trends for other variant classes.

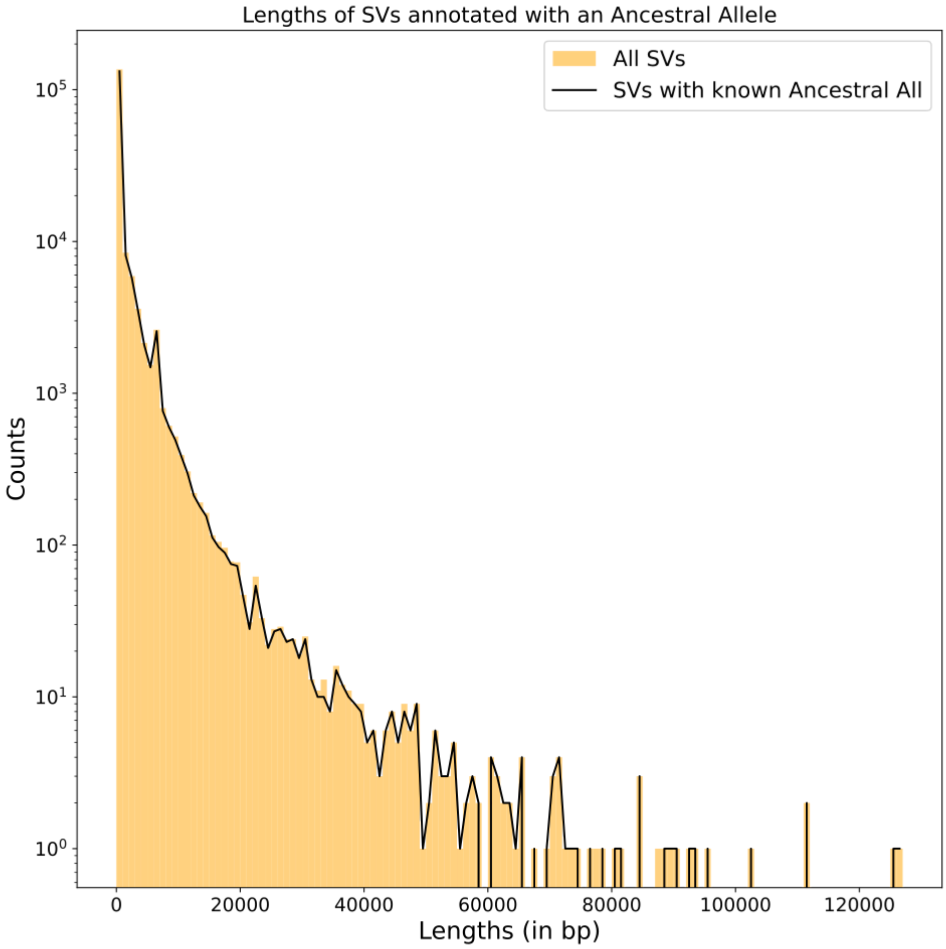

Supplementary Figure 65: Length distribution of the SVs where the ancestral allele could be inferred using the chimpanzee genome, in comparison to the full SV sites list – suggesting ancestral state polarisation is not noticeably affected by the size of the SV allele in our resource.

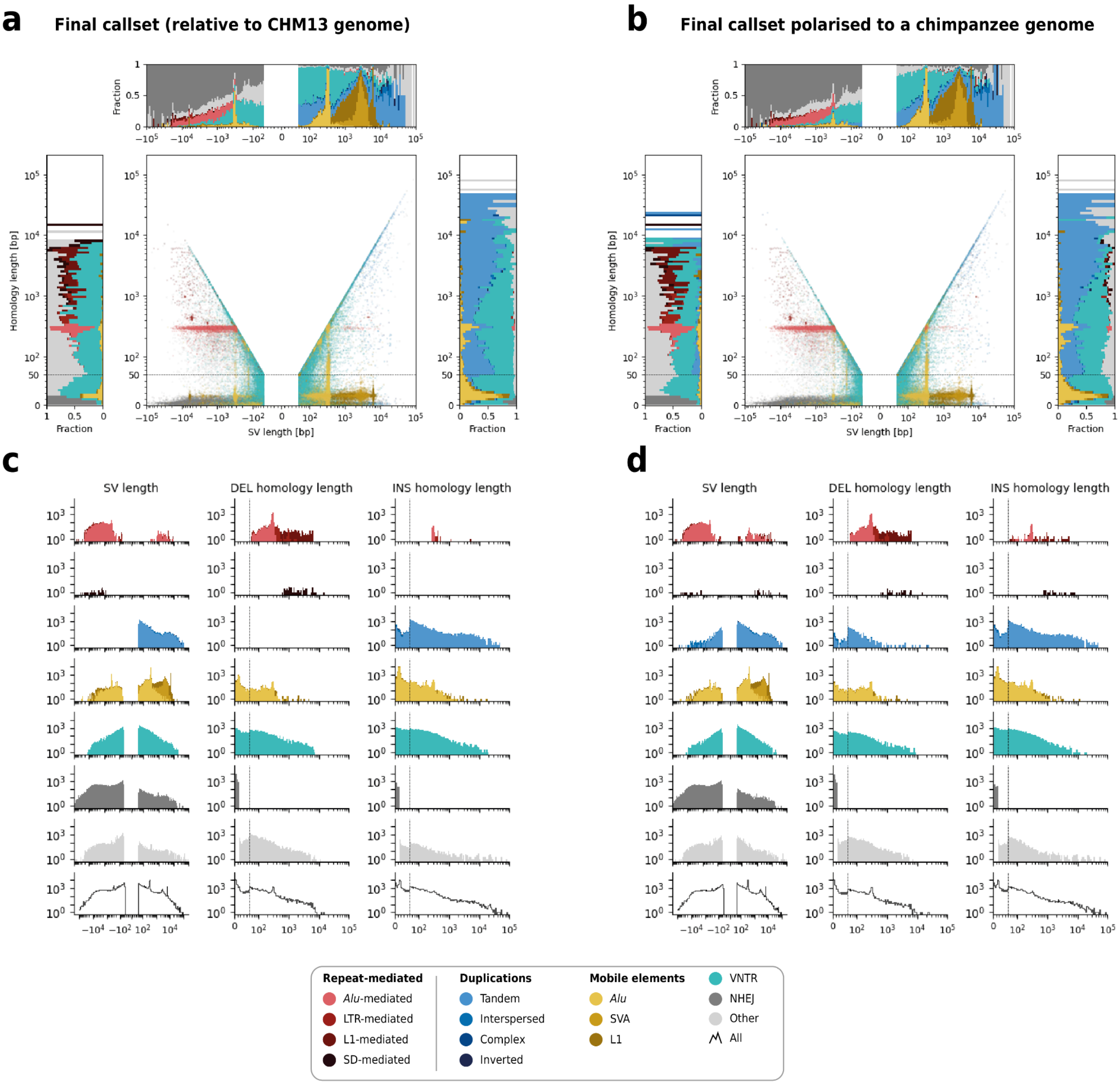

Supplementary Figure 66: Effect of callset polarisation to a chimpanzee genome on SV class distribution and homology landscape. The final callset was polarized into deletions, insertions and complex SVs based on ancestral alleles derived from a chimpanzee assembly. The final callset (a, relative to the CHM13 genome) and the callset after polarisation to the chimpanzee genome are depicted. In detail, the figure shows scatter plots of SV length versus homology length for all SVs coloured by the respective class (deletions depicted with negative length, and insertions with positive length). Marginal plots show the size-binned fraction of SV classes perpendicular to both axes, depicting deletions and insertions at the left and right, respectively. SVs directly flanked by paired repeats are shown in different shades of red, with SD-mediated SVs in black. SVs exhibiting microhomology ≤15 bp in length or blunt ended breakpoints are coloured in dark grey. Further colouring using SVAN annotations denotes the following SV classes: duplications (shades of blue), mobile elements (shades of yellow) and VNTRs (cyan). SVs not classified by SVAN are coloured in light grey. The panel **a)** is the same as in **Figure 5b** – shown here to ease visualisation. Histograms showing SV length and homology distribution are depicted for the callset relative to the CHM13 genome **c)** and the callset after polarisation **d)**.

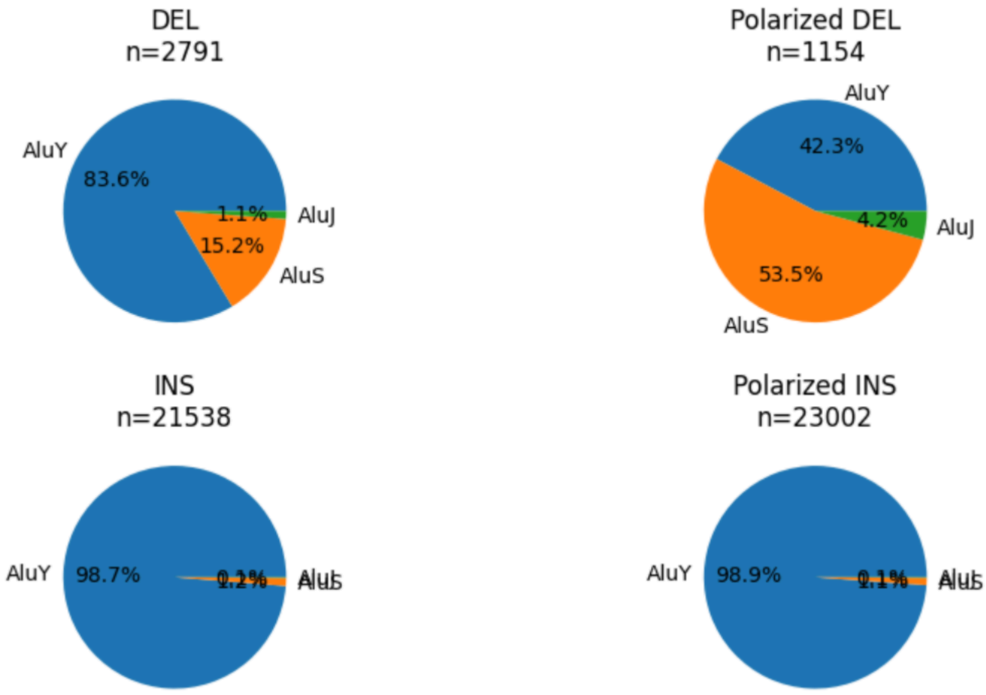

Supplementary Figure 67: Proportion of different Alu element families in SV classified as deletions or insertions in the final callset (left) and after polarisation utilising the chimpanzee genome (right).

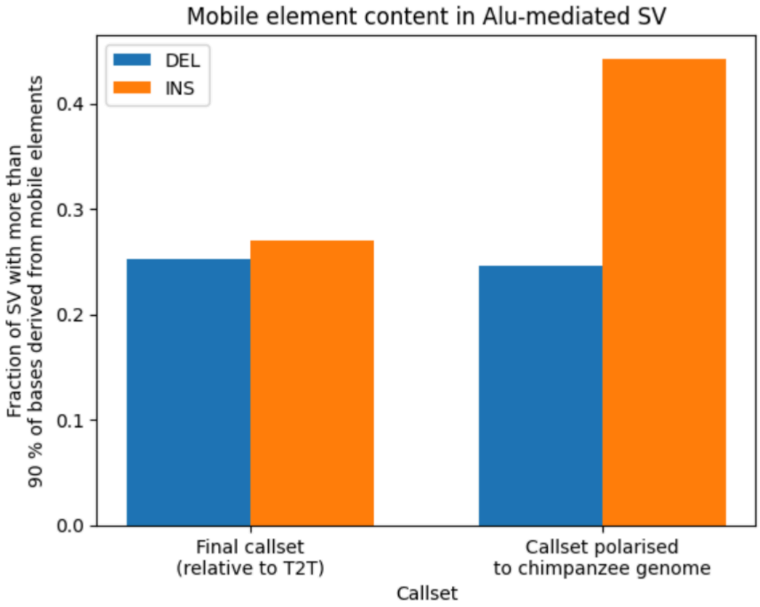

Supplementary Figure 68: Fraction of Alu-mediated SV with more than 90 % of bases derived from mobile elements. The sequence content of Alu-mediated SV was screened for bases derived from mobile elements and the combined contribution of SINE, LINE and LTR elements to the complete sequence was quantified. Shown here is the fraction of deletions (DEL) and insertions (INS) whose sequence consists of more than 90 % of mobile element annotated sequences. This information is shown for the final callset (callset relative to the CHM13/T2T genome) and for the callset after polarisation to the chimpanzee genome.

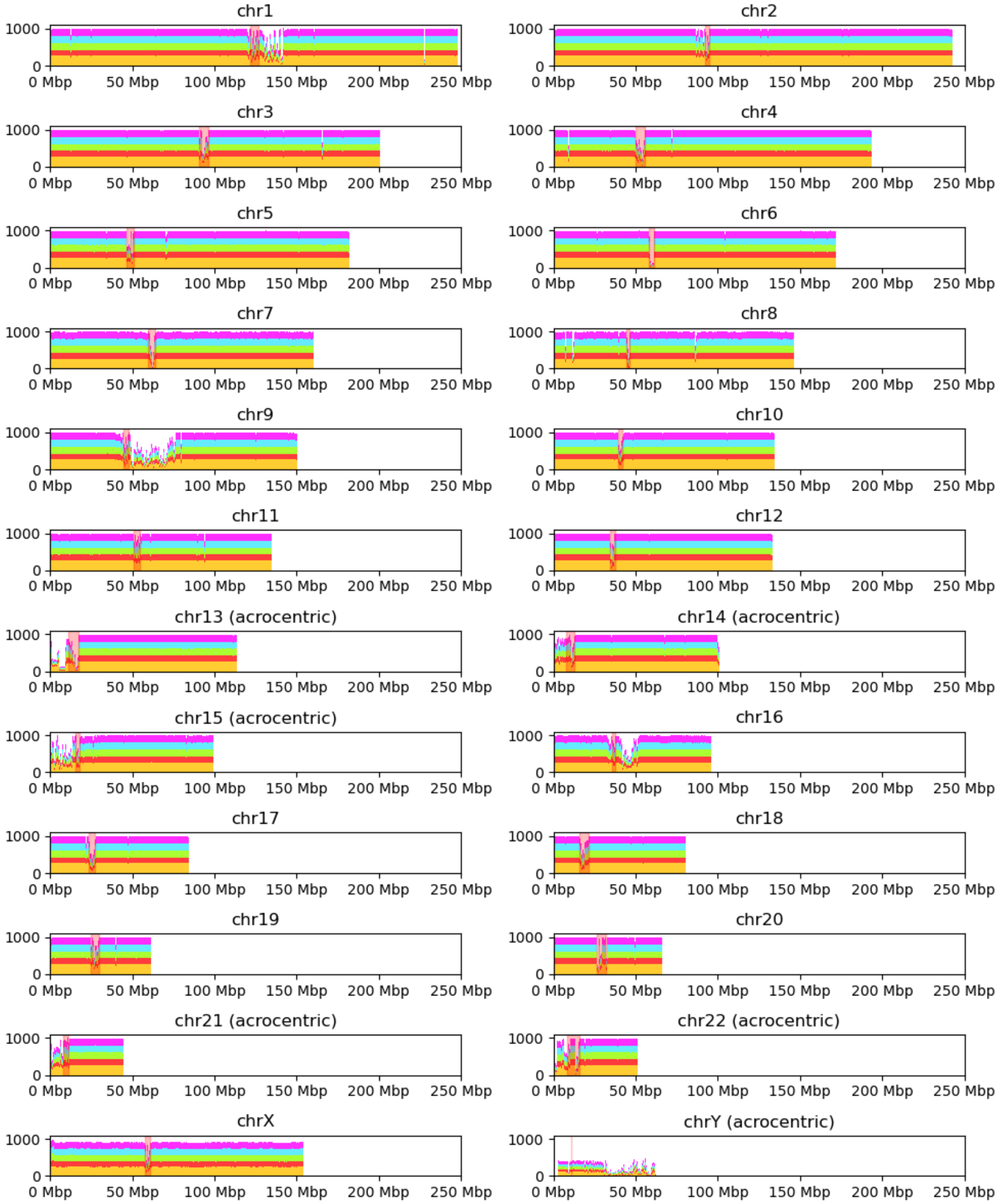

Supplementary Figure 69: Number of samples contributing at least 5x coverage by genomic region (mean across 100 kb bins). TPrimarily, these low-coverage areas correspond to the most complex regions of the genome, includingmainly peri/centromeric regions such as: (i) the alpha satellite (αSat) Higher Order Repeat (HOR) array (highlighted in red), (ii) the acrocentric arms of chromosomes 13, 14, 15, 21, 22, and Y, and (iii) the satellite-rich regions downstream of the HOR arrays on chromosomes 1, 9, and 16. These three regions jointly account for 58.2% of these lower coverage regions. Most of the remaining low-coverage regions are associated with SDs (8.4%) and other areas annotated by repeatmasker (18.7%).

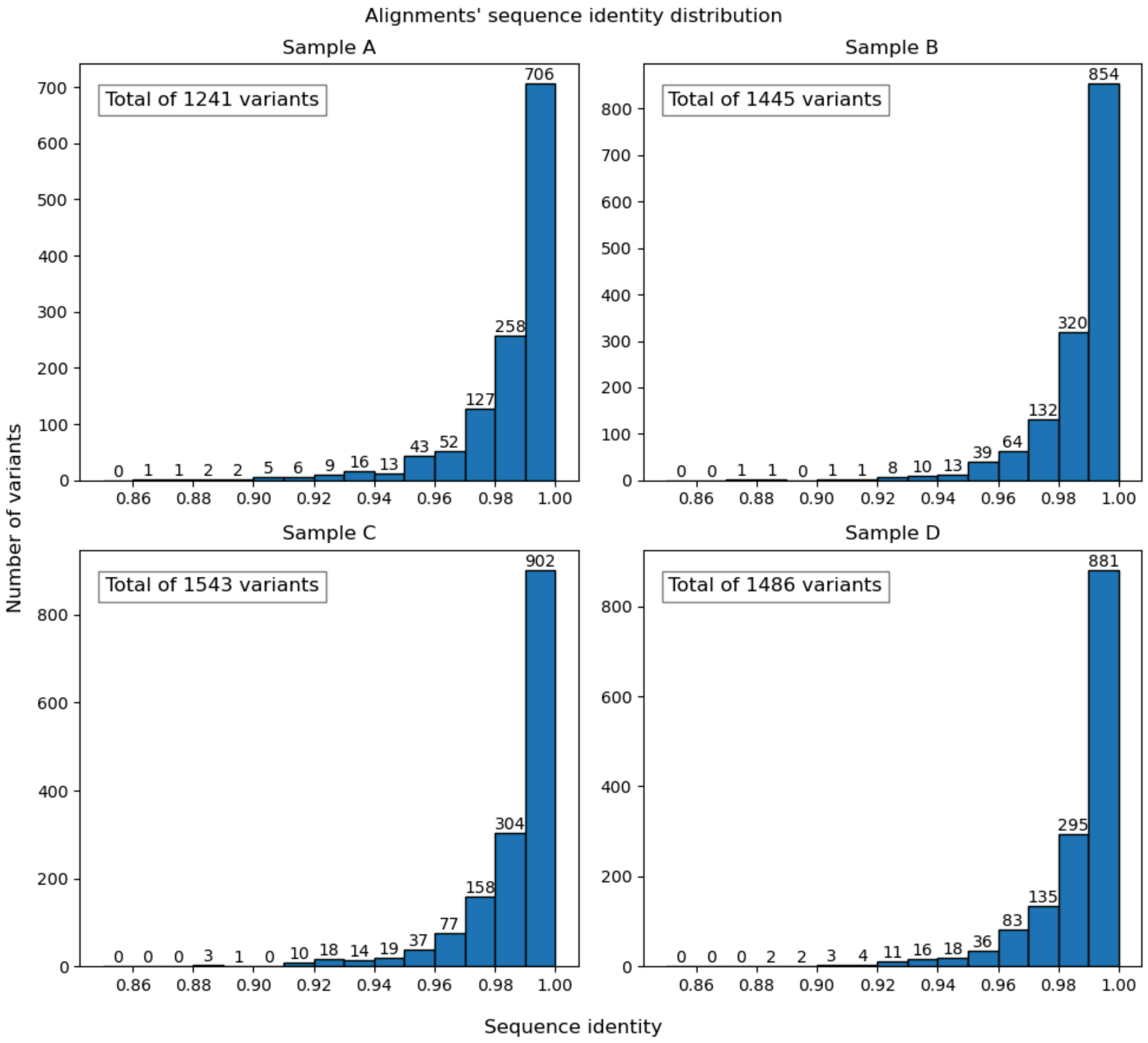

Supplementary Figure 70: Sequence identity distribution for the alignments of alleles intersecting with the Logsdon *et al.* SV callset[14] but not with the SAGA-derived SV callset, when using GraphAligner (**Note S8**). Shown here are only the alleles that had full length alignments to the graph, corresponding to 71.12%, 71.36%, 72.1% and 70.96% of the alleles intersecting with the Logsdon *et al.* callset but not with the SAGA-derived callset, for rare disease patients A, B, C and D, respectively. The majority of these variants map to known polymorphic VNTRs.

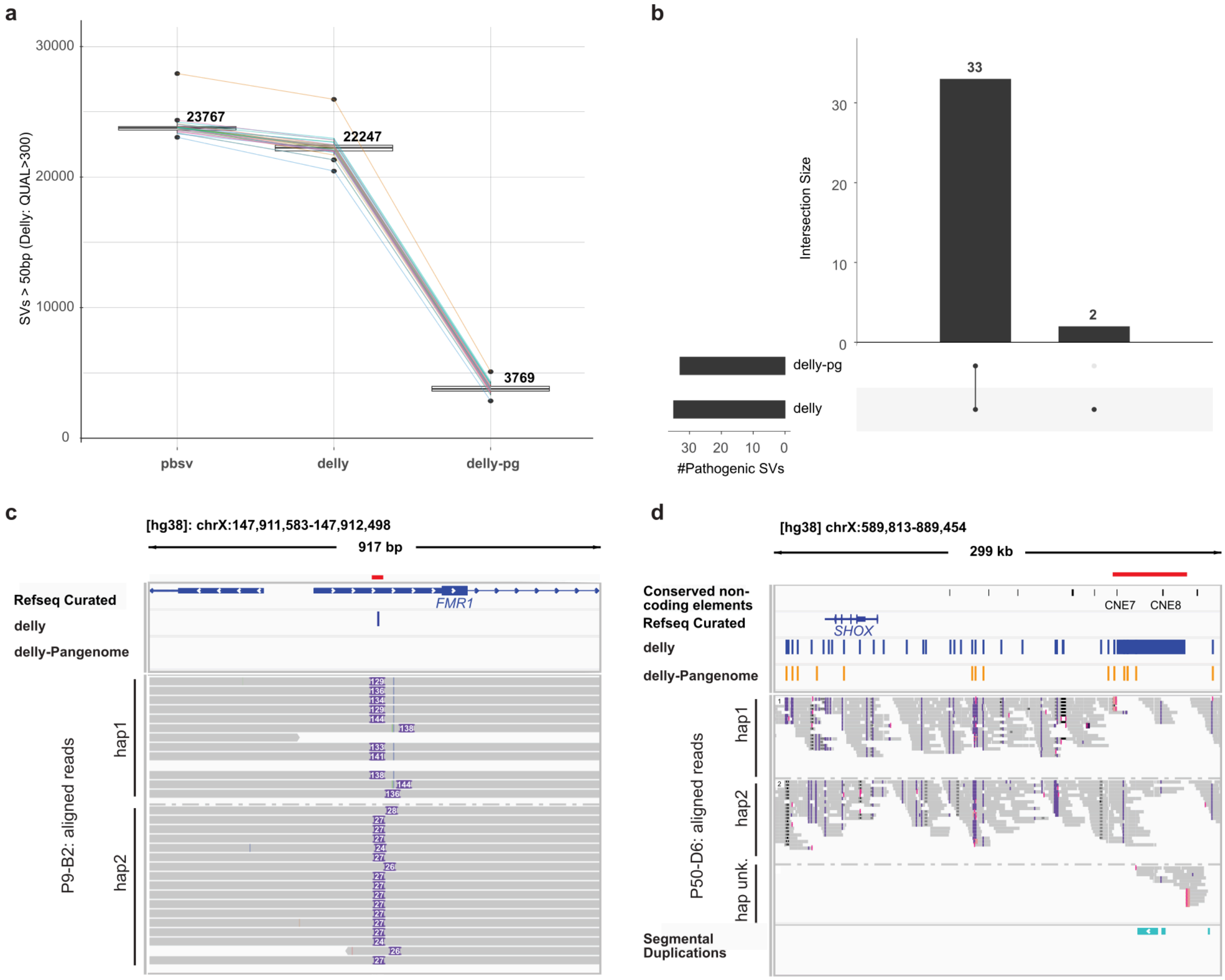

Supplementary Figure 71: Pangenome-based filtering of SVs to screen for pathogenic SVs in a previously published rare disease patient cohort with validated causal SVs. **a)** Comparison of the number of SVs reported (1) by the pbsv caller, (2) by DELLY with default settings, and by (3) by DELLY, when graph-based filtering is utilised. The median number of SVs detected in 31 rare disease patient genomes are indicated alongside the data points. The comparably high SV count in one patient sample (P1-D11; light orange) can likely be attributed to population-ancestry-related effects. **b)** An upset plot indicating the number of pathogenic SVs found by DELLY, along with the number of pathogenic SVs which are retained in graph-based filtering mode (‘delly-pg’). **c, d)** IGV views of the 2 validated pathogenic SVs filtered out in pangenome mode. **c.** A ~140 bp insertion in an STR in *FMR1* is called by DELLY (second row), but is not retained in the DELLY-pangenome mode (third row). The length of this multiallelic short tandem repeat (STR) varies in the population, with insert sizes beyond ~450 bp being drivers for the fragile X syndrome. **d.** A ~47 kbp deletion encompassing two regulatory conserved non-coding elements (CNEs) of *SHOX* is called by DELLY (second row), but not retained in DELLY-pangenome (third row). Variants in the *SHOX* CNEs exhibit recurrence and incomplete penetrance, consistent with the occasional presence of this SV in the normal population. Explaining why the variant may have been present in the reference cohort.

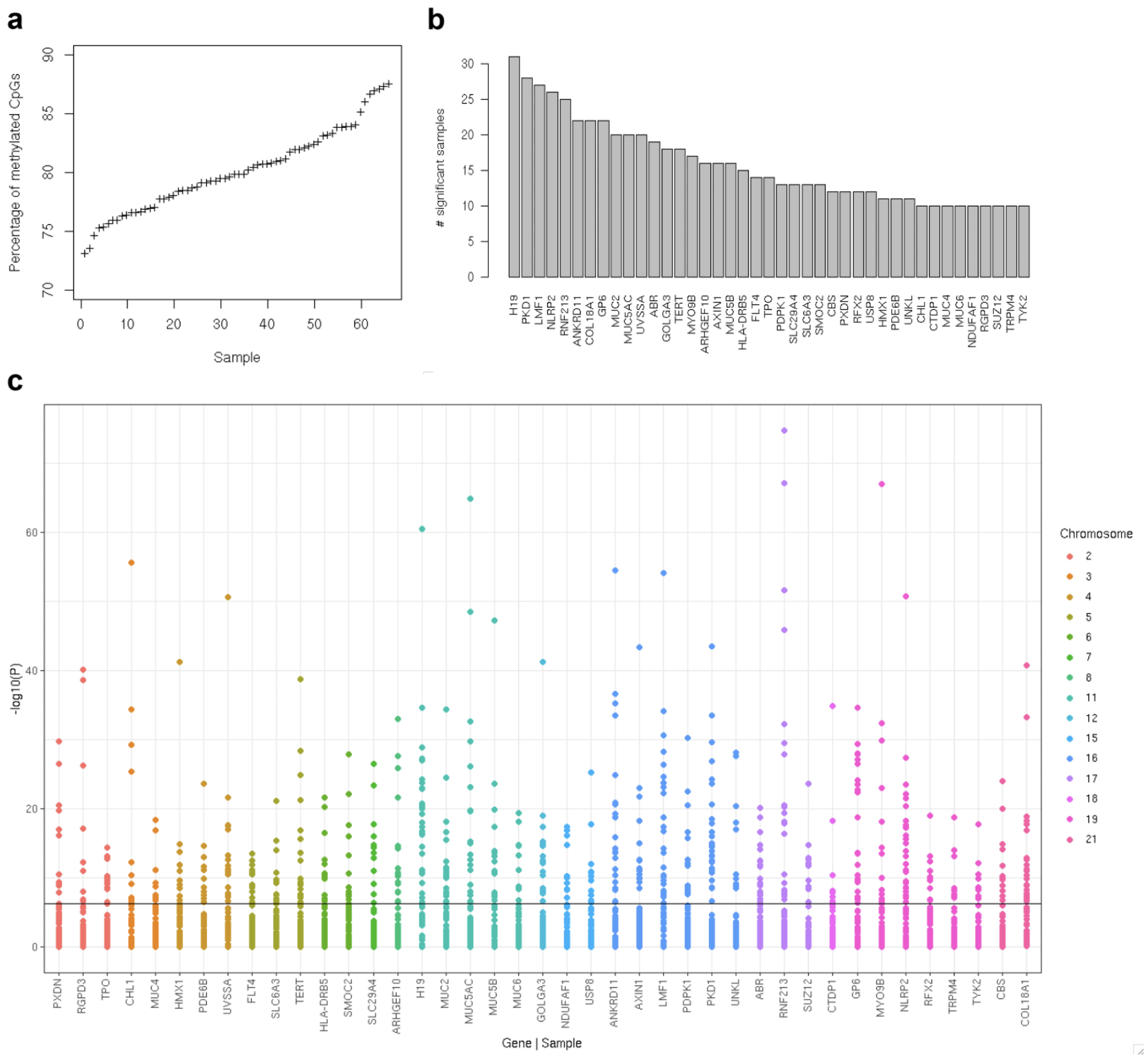

Supplementary Figure 72: Investigation of haplotype specific DNA methylation signals at 294 clinically important genes, called from ONT long-reads in a representative subset of the 1kGP samples. **a)** The percentage of all CpG sites across the 294 genes that are methylated per sample. **b)** Barplot showing the number of samples per gene for genes with 10 or more samples showing significant haplotype specific differential methylation signal. **c)** Manhattan plot showing the -log10(p) from a haplotype specific differential methylation test for the 41 genes that have 10 or more significant samples.

References

1. Martin, M. *et al.* WhatsHap: fast and accurate read-based phasing. *bioRxiv* 085050 (2016) doi:10.1101/085050.

2. Byrska-Bishop, M. *et al.* High-coverage whole-genome sequencing of the expanded 1000 Genomes Project cohort including 602 trios. *Cell* **185**, 3426–3440.e19 (2022).

3. Sudmant, P. H. *et al.* An integrated map of structural variation in 2,504 human genomes. *Nature* **526**, 75–81 (2015).
4. 1000 Genomes Project Consortium *et al.* A global reference for human genetic variation. *Nature* **526**, 68–74 (2015).
5. 1000 Genomes Project Consortium *et al.* An integrated map of genetic variation from 1,092 human genomes. *Nature* **491**, 56–65 (2012).
6. Kent, W. J. *et al.* The human genome browser at UCSC. *Genome Res.* **12**, 996–1006 (2002).
7. Handsaker, R. E. *et al.* Large multiallelic copy number variations in humans. *Nat. Genet.* **47**, 296–303 (2015).
8. Dwarshuis, N. *et al.* The GIAB genomic stratifications resource for human reference genomes. *bioRxiv* 2023.10.27.563846 (2023) doi:10.1101/2023.10.27.563846.
9. Song, L., Florea, L. & Langmead, B. Lighter: fast and memory-efficient sequencing error correction without counting. *Genome Biol.* **15**, 509 (2014).
10. Chikhi, R., Limasset, A. & Medvedev, P. Compacting de Bruijn graphs from sequencing data quickly and in low memory. *Bioinformatics* **32**, i201–i208 (2016).
11. Danecek, P. *et al.* Twelve years of SAMtools and BCFtools. *Gigascience* **10**, (2021).
12. Chaisson, M. J. P. *et al.* Multi-platform discovery of haplotype-resolved structural variation in human genomes. *Nat. Commun.* **10**, 1784 (2019).
13. Li, H. & Durbin, R. Fast and accurate short read alignment with Burrows-Wheeler transform. *Bioinformatics* **25**, 1754–1760 (2009).
14. Logsdon, G. A. *et al.* Complex genetic variation in nearly complete human genomes. *bioRxivorg* 2024.09.24.614721 (2024) doi:10.1101/2024.09.24.614721.
15. English, A. C., Menon, V. K., Gibbs, R. A., Metcalf, G. A. & Sedlazeck, F. J. Truvari: refined structural variant comparison preserves allelic diversity. *Genome Biol.* **23**, 271 (2022).
16. Benson, G. Tandem repeats finder: a program to analyze DNA sequences. *Nucleic Acids Res.* **27**, 573–580 (1999).
17. Li, H., Feng, X. & Chu, C. The design and construction of reference pangenome graphs with minigraph. *Genome Biol.* **21**, 265 (2020).
18. Li, H. Minimap2: pairwise alignment for nucleotide sequences. *Bioinformatics* **34**, 3094–3100 (2018).

19. Heller, D. & Vingron, M. SVIM-asm: structural variant detection from haploid and diploid genome assemblies. *Bioinformatics* **36**, 5519–5521 (2021).

20. Ren, J., Gu, B. & Chaisson, M. J. P. Vamos: Variable-number tandem repeats annotation using efficient motif sets. *Genome Biol.* **24**, 175 (2023).

21. Gu, B. & Chaisson, M. TRcompDB v1.0, a global reference of tandem repeat variation. Zenodo https://doi.org/10.5281/ZENODO.13263615 (2024).

22. Ruggieri, A. *et al.* Multiomic elucidation of a coding 99-mer repeat-expansion skeletal muscle disease. *Acta Neuropathol.* **140**, 231–235 (2020).

23. De Roeck, A. *et al.* An intronic VNTR affects splicing of ABCA7 and increases risk of Alzheimer's disease. *Acta Neuropathol.* **135**, 827–837 (2018).

24. Jeffares, D. C. *et al.* Transient structural variations have strong effects on quantitative traits and reproductive isolation in fission yeast. *Nat. Commun.* **8**, 14061 (2017).

25. Sedlazeck, F. J. *et al.* Accurate detection of complex structural variations using single-molecule sequencing. *Nat. Methods* **15**, 461–468 (2018).

26. Porubsky, D. *et al.* Recurrent inversion polymorphisms in humans associate with genetic instability and genomic disorders. *Cell* (2022) doi:10.1016/j.cell.2022.04.017.

27. Porubsky, D. *et al.* Inversion polymorphism in a complete human genome assembly. *Genome Biol.* **24**, 100 (2023).

28. Rautiainen, M. *et al.* Telomere-to-telomere assembly of diploid chromosomes with Verkko. *Nat. Biotechnol.* (2023) doi:10.1038/s41587-023-01662-6.

29. Sanders, A. D. *et al.* Characterizing polymorphic inversions in human genomes by single-cell sequencing. *Genome Res.* **26**, 1575–1587 (2016).

30. Yoo, D. *et al.* Complete sequencing of ape genomes. *Genomics* (2024).

31. Balachandran, P. *et al.* Transposable element-mediated rearrangements are prevalent in human genomes. *Nat. Commun.* **13**, 7115 (2022).

32. Karczewski, K. J. *et al.* The mutational constraint spectrum quantified from variation in 141,456 humans. *Nature* **581**, 434–443 (2020).

33. Ebert, P. *et al.* Haplotype-resolved diverse human genomes and integrated analysis of structural variation. *Science* **372**, (2021).

34. Smolka, M. *et al.* Detection of mosaic and population-level structural variants with Sniffles2. *Nat. Biotechnol.* **42**, 1571–1580 (2024).

35. Nurk, S. *et al.* The complete sequence of a human genome. *Science* **376**, 44–53 (2022).

36. Rautiainen, M. & Marschall, T. GraphAligner: rapid and versatile sequence-to-graph alignment. *Genome Biol.* **21**, 253 (2020).

37. Cheng, H., Concepcion, G. T., Feng, X., Zhang, H. & Li, H. Haplotype-resolved de novo assembly using phased assembly graphs with hifiasm. *Nat. Methods* **18**, 170–175 (2021).

38. Quinlan, A. R. & Hall, I. M. BEDTools: a flexible suite of utilities for comparing genomic features. *Bioinformatics* **26**, 841–842 (2010).

39. Höps, W. *et al.* HiFi long-read genomes for difficult-to-detect, clinically relevant variants. *Am. J. Hum. Genet.* **112**, 450–456 (2025).

40. *Pbsv: Pbsv - PacBio Structural Variant (SV) Calling and Analysis Tools*. (Github).

41. Benito-Sanz, S. *et al.* Identification of the first recurrent PAR1 deletion in Léri-Weill dyschondrosteosis and idiopathic short stature reveals the presence of a novelSHOXenhancer. *J. Med. Genet.* **49**, 442–450 (2012).

42. Bunyan, D. J., Baker, K. R., Harvey, J. F. & Thomas, N. S. Diagnostic screening identifies a wide range of mutations involving the SHOX gene, including a common 47.5 kb deletion 160 kb downstream with a variable phenotypic effect. *Am. J. Med. Genet. A* **161A**, 1329–1338 (2013).

43. Prodanov, T. *et al.* Locityper: targeted genotyping of complex polymorphic genes. *Bioinformatics* (2024).

44. Wagner, J. *et al.* Curated variation benchmarks for challenging medically relevant autosomal genes. *Nat. Biotechnol.* **40**, 672–680 (2022).

45. Ringel, J. & Löhr, M. The MUC gene family: their role in diagnosis and early detection of pancreatic cancer. *Mol. Cancer* **2**, 9 (2003).

46. Schmidt, K., Noureen, A., Kronenberg, F. & Utermann, G. Structure, function, and genetics of lipoprotein (a). *J. Lipid Res.* **57**, 1339–1359 (2016).

47. Baran, Y. *et al.* The landscape of genomic imprinting across diverse adult human tissues. *Genome Res.* **25**, 927–936 (2015).

48. Begemann, M. *et al.* Maternal variants in NLRP and other maternal effect proteins are associated with

multilocus imprinting disturbance in offspring. *J. Med. Genet.* **55**, 497–504 (2018).

49. Xu, J. *et al.* Epigenome-wide methylation haplotype association analysis identified HLA-DRB1, HLA-DRB5 and HLA-DQB1 as risk factors for rheumatoid arthritis. *Int. J. Immunogenet.* **50**, 291–298 (2023).
50. Liao, W.-W. *et al.* A draft human pangenome reference. *Nature* **617**, 312–324 (2023).
51. Hofmeister, R. J., Ribeiro, D. M., Rubinacci, S. & Delaneau, O. Accurate rare variant phasing of whole-genome and whole-exome sequencing data in the UK Biobank. *Nat. Genet.* **55**, 1243–1249 (2023).
52. Ebler, J. *et al.* Pangenome-based genome inference allows efficient and accurate genotyping across a wide spectrum of variant classes. *Nat. Genet.* **54**, 518–525 (2022).
53. Rausch, T. *et al.* Long-read sequencing of diagnosis and post-therapy medulloblastoma reveals complex rearrangement patterns and epigenetic signatures. *Cell Genom.* **3**, 100281 (2023).
54. Handsaker, R. E., Korn, J. M., Nemesh, J. & McCarroll, S. A. Discovery and genotyping of genome structural polymorphism by sequencing on a population scale. *Nat. Genet.* **43**, 269–276 (2011).
55. Gustafson, J. A. *et al.* High-coverage nanopore sequencing of samples from the 1000 Genomes Project to build a comprehensive catalog of human genetic variation. *Genome Res.* **34**, 2061–2073 (2024).
